# Accuracy and Reliability of Chatbot Responses to Physician Questions

**DOI:** 10.1001/jamanetworkopen.2023.36483

**Published:** 2023-10-02

**Authors:** Rachel S. Goodman, J. Randall Patrinely, Cosby A. Stone, Eli Zimmerman, Rebecca R. Donald, Sam S. Chang, Sean T. Berkowitz, Avni P. Finn, Eiman Jahangir, Elizabeth A. Scoville, Tyler S. Reese, Debra L. Friedman, Julie A. Bastarache, Yuri F. van der Heijden, Jordan J. Wright, Fei Ye, Nicholas Carter, Matthew R. Alexander, Jennifer H. Choe, Cody A. Chastain, John A. Zic, Sara N. Horst, Isik Turker, Rajiv Agarwal, Evan Osmundson, Kamran Idrees, Colleen M. Kiernan, Chandrasekhar Padmanabhan, Christina E. Bailey, Cameron E. Schlegel, Lola B. Chambless, Michael K. Gibson, Travis J. Osterman, Lee E. Wheless, Douglas B. Johnson

**Affiliations:** 1 Vanderbilt University School of Medicine, Nashville, Tennessee; 2 Department of Dermatology, Vanderbilt University Medical Center, Nashville, Tennessee; 3 Department of Allergy, Pulmonology, and Critical Care, Vanderbilt University Medical Center, Nashville, Tennessee; 4 Department of Neurology, Vanderbilt University Medical Center, Nashville, Tennessee; 5 Department of Anesthesiology, Vanderbilt University Medical Center, Nashville, Tennessee; 6 Department of Urology, Vanderbilt University Medical Center, Nashville, Tennessee; 7 Vanderbilt Eye Institute, Department of Ophthalmology, Vanderbilt University Medical, Nashville, Tennessee; 8 Department of Cardiovascular Medicine, Vanderbilt University Medical Center, Nashville, Tennessee; 9 Department of Gastroenterology, Hepatology, and Nutrition, Vanderbilt University Medical Center, Nashville, Tennessee; 10 Department of Rheumatology and Immunology, Vanderbilt University Medical Center, Nashville, Tennessee; 11 Department of Pediatric Hematology/Oncology, Vanderbilt University Medical Center, Nashville, Tennessee; 12 Department of Infectious Disease, Vanderbilt University Medical Center, Nashville, Tennessee; 13 Department of Diabetes, Endocrinology, and Metabolism, Vanderbilt University Medical Center, Nashville, Tennessee; 14 Department of Biostatistics, Vanderbilt University Medical Center, Nashville, Tennessee; 15 Division of Trauma and Surgical Critical Care, University of Miami Miller School of Medicine, Miami, Florida; 16 Department of Cardiovascular Medicine and Clinical Pharmacology, Vanderbilt University Medical Center, Nashville, Tennessee; 17 Department of Hematology/Oncology, Vanderbilt University Medical Center, Nashville, Tennessee; 18 Department of Cardiology, Washington University School of Medicine in St Louis, St Louis, Missouri; 19 Department of Radiation Oncology, Vanderbilt University Medical Center, Nashville, Tennessee; 20 Department of Surgical Oncology & Endocrine Surgery, Vanderbilt University Medical Center, Nashville, Tennessee; 21 Department of Neurological Surgery, Vanderbilt University Medical Center, Nashville, Tennessee; 22 Department of Biomedical Informatics, Vanderbilt University Medical Center, Nashville, Tennessee

## Abstract

**Importance:**

Natural language processing tools, such as ChatGPT (generative pretrained transformer, hereafter referred to as chatbot), have the potential to radically enhance the accessibility of medical information for health professionals and patients. Assessing the safety and efficacy of these tools in answering physician-generated questions is critical to determining their suitability in clinical settings, facilitating complex decision-making, and optimizing health care efficiency.

**Objective:**

To assess the accuracy and comprehensiveness of chatbot-generated responses to physician-developed medical queries, highlighting the reliability and limitations of artificial intelligence–generated medical information.

**Design, Setting, and Participants:**

Thirty-three physicians across 17 specialties generated 284 medical questions that they subjectively classified as easy, medium, or hard with either binary (yes or no) or descriptive answers. The physicians then graded the chatbot-generated answers to these questions for accuracy (6-point Likert scale with 1 being completely incorrect and 6 being completely correct) and completeness (3-point Likert scale, with 1 being incomplete and 3 being complete plus additional context). Scores were summarized with descriptive statistics and compared using the Mann-Whitney *U* test or the Kruskal-Wallis test. The study (including data analysis) was conducted from January to May 2023.

**Main Outcomes and Measures:**

Accuracy, completeness, and consistency over time and between 2 different versions (GPT-3.5 and GPT-4) of chatbot-generated medical responses.

**Results:**

Across all questions (n = 284) generated by 33 physicians (31 faculty members and 2 recent graduates from residency or fellowship programs) across 17 specialties, the median accuracy score was 5.5 (IQR, 4.0-6.0) (between almost completely and complete correct) with a mean (SD) score of 4.8 (1.6) (between mostly and almost completely correct). The median completeness score was 3.0 (IQR, 2.0-3.0) (complete and comprehensive) with a mean (SD) score of 2.5 (0.7). For questions rated easy, medium, and hard, the median accuracy scores were 6.0 (IQR, 5.0-6.0), 5.5 (IQR, 5.0-6.0), and 5.0 (IQR, 4.0-6.0), respectively (mean [SD] scores were 5.0 [1.5], 4.7 [1.7], and 4.6 [1.6], respectively; *P* = .05). Accuracy scores for binary and descriptive questions were similar (median score, 6.0 [IQR, 4.0-6.0] vs 5.0 [IQR, 3.4-6.0]; mean [SD] score, 4.9 [1.6] vs 4.7 [1.6]; *P* = .07). Of 36 questions with scores of 1.0 to 2.0, 34 were requeried or regraded 8 to 17 days later with substantial improvement (median score 2.0 [IQR, 1.0-3.0] vs 4.0 [IQR, 2.0-5.3]; *P* < .01). A subset of questions, regardless of initial scores (version 3.5), were regenerated and rescored using version 4 with improvement (mean accuracy [SD] score, 5.2 [1.5] vs 5.7 [0.8]; median score, 6.0 [IQR, 5.0-6.0] for original and 6.0 [IQR, 6.0-6.0] for rescored; *P* = .002).

**Conclusions and Relevance:**

In this cross-sectional study, chatbot generated largely accurate information to diverse medical queries as judged by academic physician specialists with improvement over time, although it had important limitations. Further research and model development are needed to correct inaccuracies and for validation.

## Key Points

**Question:**

What is the reliability of chatbot-generated responses to physician-generated medical queries?

**Findings:**

In this cross-sectional study of 33 physicians across 17 specialties who generated 284 medical questions, chatbot generated predominantly accurate information in response to these diverse medical queries as judged by these academic physician specialists. The median accuracy score was 5.5 (between almost completely and completely correct), and the median completeness score was 3.0 (complete and comprehensive).

**Meaning:**

Chatbot shows promise as a tool for providing accurate medical information in clinical settings.

## Teaser

This cross-sectional study assess the accuracy and comprehensiveness of chatbot-generated responses to physician-developed medical queries, highlighting the reliability and limitations of artificial intelligence–generated medical information.

## Introduction

The integration of natural language processing (NLP) models in health care may radically enhance the accessibility of medical information for health professionals and patients. Large language models (LLMs) are NLP tools that can understand and generate human-like text. Unlike traditional supervised deep learning models, LLMs efficiently learn from vast amounts of unannotated data through self-supervised learning and are fine-tuned on smaller annotated data sets to enhance performance on end-user–specified tasks.^1^

Chat-Generative Pre-Trained Transformer (ChatGPT), a conversational chatbot powered by GPT-3.5, an LLM with more than 175 billion parameters, has gained recent widespread popularity.^2^ ChatGPT (hereafter referred to as chatbot) is trained on a broad range of internet sources (eg, books and articles) and fine-tuned for conversational tasks using reinforcement learning from human feedback.^3^ This learning allows chatbot to incorporate the complexity of users’ intentions and proficiently respond to various end-user tasks, potentially including medical queries.

With the increasing amount of medical data and the complexity of clinical decision-making, NLP tools could assist physicians in making timely, informed decisions and improve the overall quality and efficiency of health care.^4^ Chatbot performed at or near the passing threshold for United States Medical Licensing Examination without any specialized training, suggesting its potential for medical education and clinical decision support.^5,6^ Furthermore, technology advancements have democratized medical knowledge, as patients increasingly rely on search engines and now artificial intelligence (AI) chatbots as convenient, accessible sources of medical information, reducing their reliance on health care professionals. While chatbot provides conversational, authoritative-sounding responses to complicated medical queries, these seemingly credible outputs are often incorrect, a phenomenon termed *hallucination*. This has warranted caution when considering its applications in medical practice and research.^1,7,8,9,10,11,12^ Furthermore, the reliability and accuracy of these engines are not thoroughly assessed for open-ended medical questions that physicians are likely to ask.

This study evaluates the accuracy and comprehensiveness of chatbot-generated responses to medical queries developed by physicians. Previous studies focused on closed-ended and multiple-choice questions, which do not reflect the nuances of medical decision-making. Our study provides insights into model performance in addressing medical questions developed by physicians from a diverse range of specialties; these questions are inherently subjective, open-ended, and reflect the challenges and ambiguities that physicians and, in turn, patients encounter clinically. By evaluating the model’s performance on questions developed and scored by a diverse range of physician subspecialists, this study provides an early evidence base on the reliability of chatbot for accurate and complete information in clinical settings. It will also highlight the limitations of AI-generated medical information.

## Methods

This cross-sectional study was exempt from approval by the institutional review board of Vanderbilt University because no patient-level data were used. Informed consent was not obtained by respondents but was implied by survey response. Respondents were not compensated. We followed the Strengthening the Reporting of Observational Studies in Epidemiology (STROBE) reporting guideline. The study (including data analysis) was conducted from January to May 2023. A data set of questions was generated by 33 physicians across 17 medical, surgical, and pediatric specialties (eTable 1 in Supplement 1). Fifty-nine physicians were invited (at least 1 from each major specialty; 56.0% response rate); all respondents were faculty (N = 31) or recent graduates from residency or fellowship programs (N = 2) at Vanderbilt University Medical Center. Physicians were instructed to provide questions with clear and uncontroversial answers from available medical guidelines and unchanged from the beginning of 2021 (accounting for the cutoff of the training set for chatbot based on version 3.5). All questions were subjectively chosen as representative of each physician’s subject matter expertise. To minimize bias, they were asked not to screen the questions themselves in chatbot. Each physician produced 6 questions, 3 of which had binary yes or no or right or wrong answers subjectively rated for difficulty (easy, medium, and hard). The other 3 questions were descriptive or a list of multiple correct answers, with the same difficulty ratings. An example of an easy-level difficulty descriptive question was, “What are the first-line treatments for Stage IA mycosis fungoides?” A medium-level difficulty descriptive question was, “Which patients with well-differentiated thyroid cancer should receive postoperative radioactive iodine ablation?” A hard-level difficulty binary question was, “Can we start angiotensin receptor-neprilysin inhibitors immediately after discontinuing an angiotensin-converting enzyme inhibitor?” For additional example questions and answers, see Table 1 and the eAppendix in Supplement 1. To assess physician agreement and generate additional data, the senior authors (L.E.W. and D.B.J.) provided and rated a data set of 44 melanoma- or immunotherapy-specific questions. Because most physicians were specialists, the senior authors produced and rated a third data set of 60 questions encompassing 10 common medical conditions (eTable 2 in Supplement 1). Six questions were generated for each common medical condition with the same question classification (binary vs descriptive and difficulty level).

**Table 1.** Examples of Chatbot-Generated Answers to Medical Questions From Physicians^a^

Question set, physician specialty, question, type, question difficulty	Physician-generated question	Chatbot-generated answer	Accuracy rating	Completeness rating	Rating explanation
Multispecialty, infectious disease, descriptive, easy	What oral antibiotics may be used for the treatment of MRSA infections?	Oral antibiotics that may be used for the treatment of MRSA infections include doxycycline, clindamycin, tetracycline, minocycline, linezolid, daptomycin, and telavancin. It is important to note that treatment of MRSA infections should be guided by susceptibility testing and the clinical scenario, and treatment should be initiated in consultation with an infectious disease specialist.	3.0	1.0	The answer included some oral options but also options that are not available orally. The answer also left out one of most important oral antibiotics for treatment, trimethoprim-sulfamethoxazole.
Multispecialty, surgical oncology, binary, hard	Is radioactive iodine ablation indicated for a patient with a 2-cm intrathyroidal classic type papillary thyroid carcinoma after total thyroidectomy with negative margins and no lymph node involvement?	No, radioactive iodine ablation is not typically indicated for a patient with a 2-cm intrathyroidal classic type papillary thyroid carcinoma after total thyroidectomy with negative margins and no lymph node involvement. The ATA and the ACS recommends that radioactive iodine ablation therapy should be offered to patients who had total thyroidectomy with a papillary thyroid carcinoma >1 cm or with extrathyroidal extension, and for patients with any T3 or T4 tumors, and for patients with lymph node involvement. In this specific case, the patient has a classic type papillary thyroid carcinoma, size of 2 cm, that is intrathyroidal, and has negative surgical margins and no lymph node involvement. Therefore, the patient is considered to have a low risk of recurrence, and the benefit of radioactive iodine ablation therapy may be limited. However, this decision should be taken by a multidisciplinary team and based on the patient’s individual characteristics and preferences.	6.0	3.0	NA
Melanoma, immunotherapy, dermatology or medical oncology, descriptive, medium	What stage would a melanoma with 1.2-mm depth, no ulceration, and no lymph node involvement be?	A melanoma with a depth of 1.2 mm, no ulceration, and no lymph node involvement would be considered Stage IA.	1.0	NA	It should be Stage IB T2aN0M0.
Common conditions: acute coronary syndrome, dermatology or medical oncology, binary, easy	Should patients with a history of acute myocardial infarction receive a statin?	Yes, patients with a history of AMI should generally be treated with a statin. The ACC and AHA guidelines recommend that all patients who have had an AMI should receive high-intensity statin therapy as soon as possible, ideally within 24 h of the event. The goal of statin therapy for these patients is to lower their LDL cholesterol levels, which can help prevent further cardiac events, such as another heart attack or stroke. In addition, the guidelines state that patients who have had an AMI should continue to receive high-intensity statin therapy indefinitely, regardless of their LDL cholesterol level, as long as there are no contraindications or significant adverse effects.	6.0	3.0	NA

Abbreviations: ACC, American College of Cardiology; ACS, American College of Surgeons; AHA, American Heart Association; AMI, acute myocardial infarction; ATA, American Thyroid Association; LDL, low-density lipoprotein; MRSA, methicillin-resistant *Staphylococcus aureus*; NA, not applicable.

^a^ The accuracy scale was a 6-point Likert scale (with 1 indicating completely incorrect; 2, more incorrect than correct; 3, approximately equal correct and incorrect; 4, more correct than incorrect; 5, nearly all correct; and 6, completely correct), and the completeness scale was a 3-point Likert scale (with 1 indicating incomplete [addresses some aspects of the question, but significant parts are missing or incomplete]; 2, adequate [addresses all aspects of the question and provides the minimum amount of information required to be considered complete]; and 3, comprehensive [addresses all aspects of the question and provides additional information or context beyond what was expected]). Answers that were completely incorrect on the accuracy scale (score of 1) were not graded on comprehensiveness.

To ensure consistency, all questions were entered into chatbot by 1 investigator (R.S.G.), who prompted the chatbot with the phrase “Be specific and incorporate any applicable medical guidelines” using unconditional prompts (new chats) for each question. Physicians who created the questions assessed the accuracy of the AI-generated answers based on their medical expertise, using 2 predefined scales of accuracy and completeness.

The accuracy scale was a 6-point Likert scale (with 1 indicating completely incorrect; 2, more incorrect than correct; 3, approximately equal correct and incorrect; 4, more correct than incorrect; 5, nearly all correct; and 6, completely correct). The completeness scale was a 3-point Likert scale (with 1 indicating incomplete [addresses some aspects of the question, but significant parts are missing or incomplete]; 2, adequate [addresses all aspects of the question and provides the minimum amount of information required to be considered complete]; and 3, comprehensive [addresses all aspects of the question and provides additional information or context beyond what was expected]). Completely incorrect answers (accuracy score of 1) were not graded on comprehensiveness.

**Figure 1.**
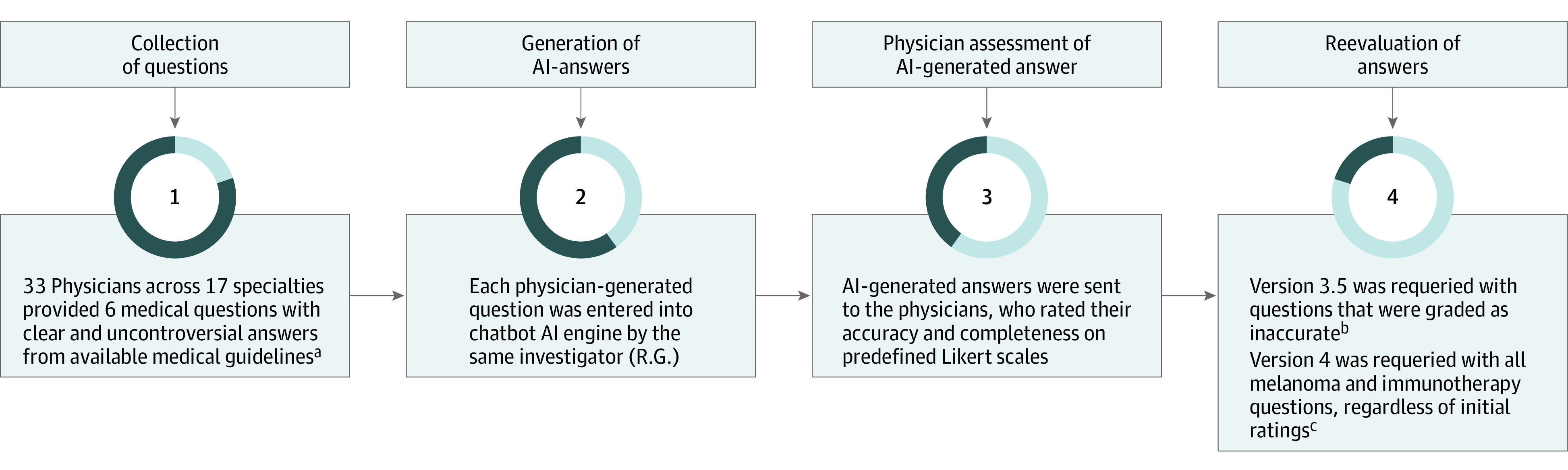
Methods AI indicates artificial intelligence. ^a^D.B.J. and L.E.W. scored 2 separate data sets of melanoma and immunotherapy and common conditions questions. ^b^Regenerated answers were created 8 to 17 days after initial answers. ^c^Regenerated answers were created 90 days after initial answers.

To assess accuracy and reproducibility over time, chatbot was requeried with questions that initially generated inaccurate answers (<3 on the accuracy scale) 8 to 17 days later. This timeline was dependent on the time physicians responded with their scores. Physicians rescored the updated AI answers. To comprehensively assess model performance and consistency using the latest, most advanced version, all melanoma and immunotherapy questions, regardless of initial scores, were regenerated and rescored using chatbot (most advanced version available, based on version 4) (Figure 1).

### Statistical Analysis

Score results were listed descriptively (median [IQR] values and mean [SD] vallues) and were compared between groups using the Mann-Whitney *U* test or the Kruskal-Wallis test (GraphPad Prism, version 9.5.1). Regraded questions were compared using the Wilcoxon signed rank test (GraphPad Prism, version 9.5.1). In the melanoma or immunotherapy and the common conditions data set, interrater agreement was graded using the weighted κ statistic across all scores (1-6 for accuracy and 1-3 for completeness) (R package “irr”; R, version 4.3.1 [The R Project for Statistical Computing]). A 2-sided *P* < .05 was considered statistically significant.

## Results

### Multispecialty Analysis

Chatbot-generated answers were evaluated using 180 questions provided by 33 physicians (31 faculty members and 2 recent graduates from residency or fellowship programs) across 17 specialties, including 3 descriptive and 3 binary questions at varying difficulty levels (easy, medium, and hard). One author provided 2 descriptive question sets (eAppendix in Supplement 1). Among 180 chatbot-generated answers, the median accuracy score was 5.0 (IQR, 1.0-6.0) (mean [SD] score, 4.4 [1.7]), and the median completeness score was 3.0 (IQR, 2.0-3.0) (mean [SD] score, 2.4 [0.7]) (Table 2; eTable 3 in Supplement 1). Seventy-one questions (39.4%) were scored at the highest level of accuracy (accuracy score of 6.0), and 33 questons (18.3%) were scored as nearly all correct (accuracy score of 5.0). Conversely, the answers to 15 questions (8.3%) were scored as completely incorrect (accuracy score of 1.0). Inaccurate answers, receiving accuracy scores of 2.0 or lower (n = 36), were most commonly in response to physician-rated hard questions with either binary answers (n = 8 [22.2%]) or descriptive answers (n = 7 [19.4%]), but they were distributed across all categories. The answers to 96 questions (53.3%) were scored as comprehensive, 47 (26.1%) as adequate, and 22 (12.2%) as incomplete. Accuracy and completeness were modestly correlated (Spearman *r* = 0.4 [95% CI, 0.3-0.5]; *P* < .01; α = .05) across all questions.

**Table 2.** Accuracy Scores for Artificial Intelligence–Generated Answers to Medical Questions^a^

Specialty	Overall	Question type			Question difficulty			
		Descriptive	Binary	*P* value	Easy	Medium	Hard	*P* value
Multispecialty (n = 180)								
Median (IQR)	5.0 (1.0-6.0)	5.0 (3.0-6.0)	5.0 (3.0-6.0)	.40	5.0 (3.0-6.0)	5.0 (3.0-6.0)	5.0 (2.3-6.0)	.30
Mean (SD)	4.4 (1.7)	4.3 (1.7)	4.5 (1.7)		4.6 (1.7)	4.3 (1.7)	4.2 (1.8)	
Melanoma and immunotherapy (n = 44)								
Median (IQR)	6.0 (5.0-6.0)	6.0 (5.0-6.0)	6.0 (5.0-6.0)	.70	6.0 (6.0-6.0)	5.5 (3.9-6.0)	5.8 (5.0-6.0)	.05
Mean (SD)	5.2 (1.3)	5.1 (1.5)	5.4 (1.2)		5.9 (0.3)	4.8 (1.7)	5.3 (1.1)	
Common conditions (n = 60)								
Median	6.0 (5.5-6.0)	6.0 (5.5-6.0)	6.0 (5.9-6.0)	.08	6.0 (6.0-6.0)	6.0 (5.5-6.0)	5.8 (5.5-6.0)	.07
Mean (SD)	5.7 (0.7)	5.6 (0.6)	5.8 (0.8)		5.9 (0.4)	5.6 (1.0)	5.6 (0.1)	
All (N = 284)								
Median (IQR)	5.5 (4.0-6.0)	5.0 (3.4-6.0)	6.0 (4.0-6.0)	.07	6.0 (5.0-6.0)	5.5 (3.4-6.0)	5.0 (4.0-6.0)	.05
Mean (SD)	4.8 (1.6)	4.7 (1.6)	4.9 (1.6)		5.0 (1.5)	4.7 (1.7)	4.6 (1.6)	

^a^ The accuracy scale was a 6-point Likert scale (with 1 indicating completely incorrect; 2, more incorrect than correct; 3, approximately equal correct and incorrect; 4, more correct than incorrect; 5, nearly all correct; and 6, completely correct).

### Question Type and Difficulty Level

Among both descriptive and binary questions, the median accuracy scores for easy, medium, and hard answers were 5.0 (IQR, 3.0-6.0; mean [SD] score, 4.6 [1.7]), 5.0 (IQR, 3.0-6.0; mean [SD] score, 4.3 [1.7]), and 5.0 (IQR, 2.3-6.0; mean [SD] score, 4.2 [1.8]), respectively, and were similar between groups (*P* = .40 determined by the Kruskal-Wallis test) (Table 2; eTable 3 in Supplement 1). The median completeness scores for all answers were 3.0 (IQR, 2.0-3.0; mean [SD] score, 2.6 [0.7]) for easy, 3.0 (IQR, 2.0-3.0; mean [SD] score, 2.4 [0.7]) for medium, and 2.5 (IQR, 2.0-3.0; mean [SD] score, 2.4 [0.7]) for hard answers, with no differences based on difficulty (*P* = .30 determined by the Kruskal-Wallis test).

**Figure 2.**
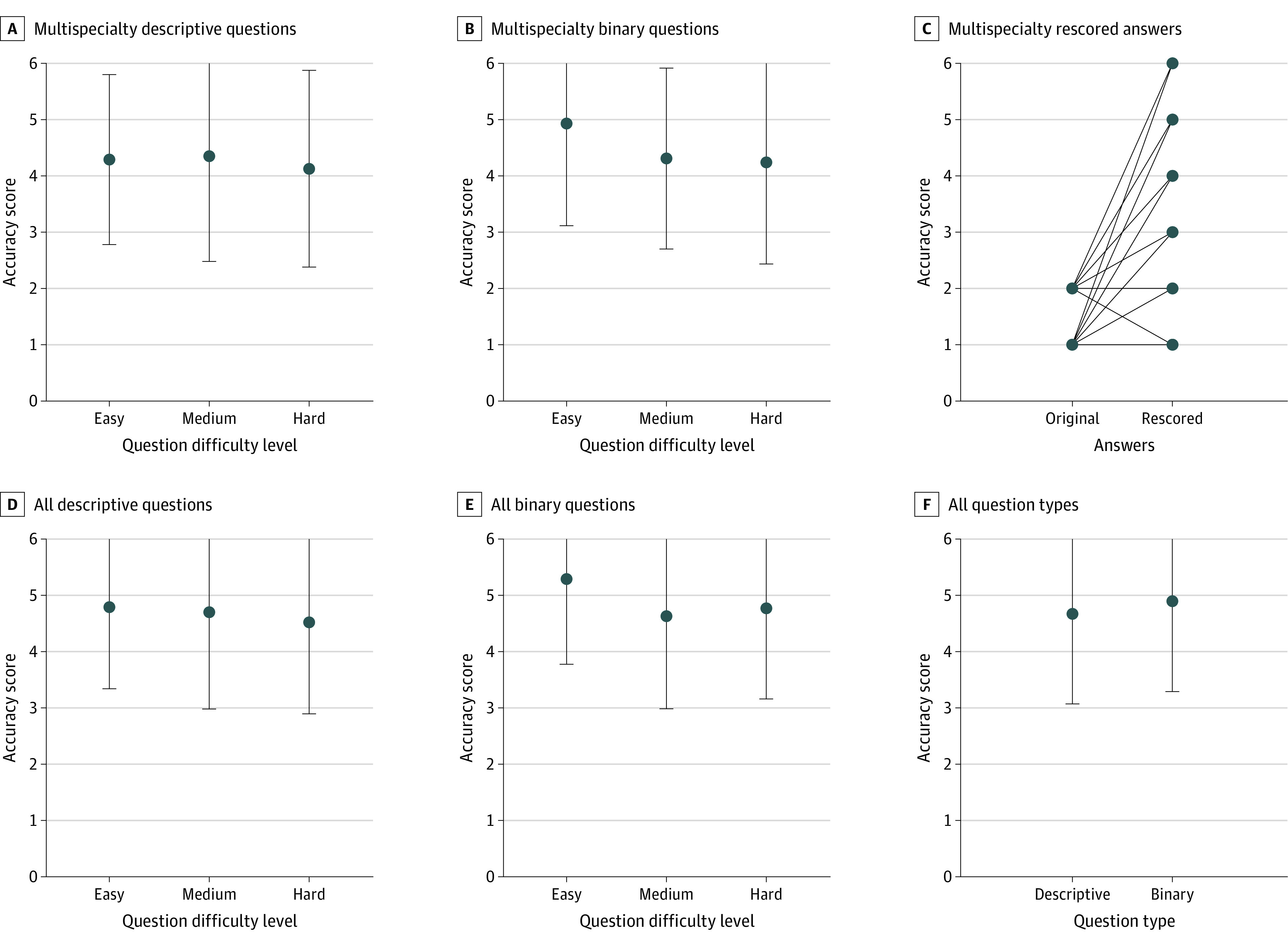
Accuracy of Chatbot-Generated Answers Accuracy of artificial intelligence answers from multispecialty questions (A-C [*P* < .01 for panel C]) or all questions (multispecialty, melanoma and immunotherapy, and common medical conditions; D-F [*P* = .03 for panel E]). A, Among all descriptive questions in the multispecialty analysis, median accuracy scores were 5.0 (IQR, 3.0-6.0) (mean [SD] score, 4.9 [1.5]) for easy, 5.0 (IQR, 3.0-6.0) (mean [SD] score, 4.4 [1.9]) for medium, and 5.0 (IQR, 3.0-6.0) (mean [SD] score, 4.1 [1.8]) for hard questions (*P* = .70 determined by the Kruskal-Wallis test). B, Among all binary questions in the multispecialty analysis, median accuracy scores were 6.0 (IQR, 5.0-6.0) (mean [SD] score, 4.9 [1.8]) for easy, 4.0 (IQR, 3.0-6.0) (mean [SD] score, 4.3 [1.6]) for medium, and 5.0 (IQR, 1.0-6.0) (mean [SD] score, 4.2 [1.8]) for hard answers (*P* = .10 determined by the Kruskal-Wallis test). C, Of 36 questions with accuracy scores of 2 or lower, 34 were requeried or regraded 8 to 17 days later. The median accuracy score for original questions was 2.0 (IQR, 1.0-2.0) (mean [SD] score, 1.6 [0.5]) compared with 4.0 (IQR, 2.0-5.3) (mean [SD] score, 3.9 [1.8]) for rescored answers (*P* < .01 determined by Wilcoxon signed rank test). D, Among all descriptive questions, median accuracy scores for easy, medium, and hard questions were 5.3 (IQR, 3.0-6.0) (mean [SD] score, 4.8 [1.5]) for easy, 5.5 (IQR, 3.3-6.0) (mean [SD] score, 4.7 [1.7]) for medium, and 5.0 (IQR, 3.6-6.0) (mean [SD] score, 4.5 [1.6]) for hard questions (*P* = .40 determined by the Kruskal-Wallis test). E, Among all binary questions, median accuracy scores were 6.0 (IQR, 5.0-6.0) (mean [SD] score, 5.3 [1.5]) for easy, 5.5 (IQR, 3.4-6.0) (mean [SD] score, 4.6 [1.6) for medium, and 5.5 (IQR, 4.0-6.0) (mean [SD] score, 4.8 [1.6]) for hard questions, which resulted in a significant difference among groups (*P* = .03 determined by the Kruskal-Wallis test). F, Median accuracy scores were 5.0 (IQR, 3.4-6.0) (mean [SD] score, 4.7 [1.6]) for all descriptive questions and 6.0 (IQR, 4.0-6.0) (mean [SD] score, 4.9 [1.6]) for binary questions (*P* = .07 determined by Mann-Whitney *U* test).

Both descriptive and binary questions were analyzed to assess chatbot’s performance on distinct categories. The median accuracy score of descriptive questions (n = 93) was 5.0 (IQR, 3.0-6.0; mean [SD] score, 4.3 [1.7]), and the median accuracy score of binary questions (n = 87) was also 5.0 (IQR, 3.0-6.0; mean [SD] score, 4.5 [1.7]), similar between groups (*P* = .30 determined by the Mann-Whitney *U* test) (Table 2). Among descriptive questions, the median accuracy scores for easy, medium, and hard questions were 5.0 (IQR, 3.0-6.0; mean [SD] score, 4.9 [1.5]), 5.0 (IQR, 3.0-6.0; mean [SD], 4.4 [1.9]), and 5.0 (IQR, 3.0-6.0; mean [SD] score, 4.1 [1.8]), respectively (*P* = .70 determined by the Kruskal-Wallis test) (Table 2; Figure 2A).

Among binary questions, the median accuracy scores for easy, medium, and hard answers were 6.0 (IQR, 5.0-6.0; mean [SD] score, 4.9 [1.8]), 4.0 (IQR, 3.0-6.0; mean [SD] score, 4.3 [1.6]), and 5.0 (IQR, 2.0-6.0; mean [SD] score, 4.2 [1.8]), respectively (*P* = .10 determined by the Kruskal-Wallis test) (Table 2; Figure 2B). Overall, the results suggested no major differences in the accuracy and completeness of chatbot-generated answers for descriptive or binary questions across levels of difficulty.

### Internal Validation: Rescored Analysis of Incorrect AI-Generated Answers

Of 36 inaccurate answers that received a score of 2.0 or lower on the accuracy scale, 34 were rescored by physicians to evaluate the reproducibility of answers over time (Table 3). Notably, scores generally improved with 26 questions improving, 7 remaining the same, and 1 decreasing in accuracy. The median accuracy score for original questions was 2.0 (IQR, 1.0-2.0; mean [SD] score, 1.6 [0.5]) compared with the median score of 4.0 (IQR, 2.0-5.3; mean [SD] score, 3.9 [1.8]) for rescored answers (*P* < .001 determined by the Wilcoxon signed rank test) (Figure 2C; eTable 4 in Supplement 1).

**Table 3.** Reevaluation of Inaccurate Chatbot-Generated Answer^a^

Specialty, question type, question difficulty, date of AI-generated answer	Physician-generated question	AI-generated answer	Accuracy rating	Completeness rating
Neurology, descriptive, hard, 1/9/23	For a patient with left hemispheric stroke due to high-grade atherosclerotic disease of the left MCA, what is the optimal duration of DAPT?	According to the AHA/ASA guidelines, the optimal duration of DAPT for a patient with a left hemispheric stroke due to high-grade atherosclerotic disease of the left MCA is generally considered to be at least 1 y. The AHA/ASA recommends continuing DAPT with aspirin and either clopidogrel or ticagrelor for at least 1 y after a stroke or TIA for patients with high-grade intracranial stenosis or occlusion of a major cerebral artery, unless there is a contraindication or the patient is at high risk of bleeding.	1.0	NA
Neurology, descriptive, hard, 1/20/23	For a patient with left hemispheric stroke due to high-grade atherosclerotic disease of the left MCA, what is the optimal duration of DAPT?	The optimal duration of DAPT for a patient with left hemispheric stroke due to high-grade atherosclerotic disease of the left MCA is not well established. However, guidelines from the AHA/ASA recommend DAPT for at least 90 d for patients who have had a stroke or high-risk TIA and who are able to tolerate the therapy. The guideline suggests that DAPT with aspirin and clopidogrel for 90 d after stroke or high-risk TIA is a Class I recommendation (level of evidence A). However, longer-term DAPT (6-12 mo) may be considered for certain patients at high risk of recurrent stroke, such as those with intracranial stenosis or prior stroke or TIA while receiving aspirin therapy. It is important to note that the duration of DAPT should be individualized based on the patient’s risk of bleeding, their compliance, and the possibility of interactions with other medications. Also, the use of aspirin and clopidogrel should be tailored based on the individual patient’s characteristics, with close monitoring and reevaluation of the need for therapy at regular intervals.	6.0	3.0

Abbreviations: AHA/ASA, American Heart Association/American Stroke Association; DAPT, dual antiplatelet therapy; MCA, middle cerebral artery; NA, not applicable, TIA, transient ischemic attack.

^a^ The accuracy scale was a 6-point Likert scale (with 1 indicating completely incorrect; 2, more incorrect than correct; 3, approximately equal correct and incorrect; 4, more correct than incorrect; 5, nearly all correct; and 6, completely correct), and the completeness scale was a 3-point Likert scale (with 1 indicating incomplete [addresses some aspects of the question, but significant parts are missing or incomplete]; 2, adequate [addresses all aspects of the question and provides the minimum amount of information required to be considered complete], and 3, comprehensive [addresses all aspects of the question and provides additional information or context beyond what was expected]). Answers that were completely incorrect on the accuracy scale (score of 1) were not graded on comprehensiveness.

### Melanoma and Immunotherapy Analysis

To further assess performance and judge interrater variability, 2 physicians (D.B.J. and L.E.W.) independently assessed additional questions on melanoma diagnosis and treatment as well as cancer immunotherapy use from existing guidelines before 2021. Among 44 AI-generated answers, the median accuracy score was 6.0 (IQR, 5.0-6.0; mean [SD] score, 5.2 [1.3]), and the median completeness score was 3.0 (IQR, 2.5-3.0; mean [SD] score, 2.6 [0.8]) (Table 2). The median accuracy scores of descriptive and binary questions were 6.0 (IQR, 5.0-6.0; mean [SD] score, 5.1 [1.5]) and 6.0 (IQR, 5.0-6.0; mean [SD] score, 5.4 [1.2]), respectively (*P* = .70 determined by the Mann-Whitney *U* test). Among both descriptive and binary questions, the median accuracy scores for easy, medium, and hard answers were 6.0 (IQR, 6.0-6.0; mean [SD] score, 5.9 [0.3]), 5.5 (IQR, 3.9-6.0; mean [SD] score, 4.8 [1.7]), and 5.8 (IQR, 5.0-6.0; mean [SD] score, 5.3 [1.1]), respectively, with a significant trend (*P* = .046 determined by the Kruskal-Wallis test). There was high interrater agreement (weighted κ = 0.7; *P* < .001) for accuracy and moderate agreement (weighted κ = 0.5; *P* < .001) for completeness (eTable 5 in Supplement 1).

### Internal Validation: Rescored Analysis With Version 4

To comprehensively evaluate model performance and consistency using the latest, most advanced version of chatbot (ie, version 4), all melanoma and immunotherapy questions (n = 44), regardless of initial scores (generated from chatbot version 3.5), were regenerated and rescored using version 4 of chatbot 90 days after initial answers were generated and scored by 2 authors (D.B.J. and L.E.W.). The mean (SD) accuracy scores improved from 5.2 (1.5) (median score, 6.0 [IQR, 5.0-6.0]) to 5.7 (0.8) (median score, 6.0 [IQR, 6.0-6.0]) for rescored answers (*P* = .003 determined by Wilcoxon signed rank test) (eTable 6 in Supplement 1). Among original answers that received an accuracy score of less than 6.0 (n = 31), 24 (77.4%) received improved accuracy scores, 1 (3.2%) remained the same, and 6 (19.3%) received worse scores. Completeness scores also improved (original mean [SD] score, 2.6 1.0]; median score, 3.0 [IQR, 3.0-3.0] vs rescored: mean [SD] score 2.8 [0.5]; median score, 3.0 [IQR, 3.0-3.0]) (*P* = .001 determined by Wilcoxon signed rank test). There was moderate interrater agreement (weighted κ = 0.4; *P* = .006) for accuracy and poor agreement (weighted κ = 0.01; *P* = .93) for completeness.

### Common Conditions Analysis

To assess performance further in general questions widely pertinent across practitioners, 2 physicians (L.E.W. and D.B.J.) generated and graded questions related to 10 common medical conditions (eTable 2 in Supplement 1). Among 60 AI-generated answers, the median accuracy score was 6.0 (IQR, 5.5-6.0; mean [SD] score, 5.7 [0.7]), and the median completeness score was 3.0 (IQR, 3.0-3.0; mean [SD] score, 2.8 [0.5]) (Table 2; eTable 3 in Supplement 1). The median accuracy score was 6.0 (IQR, 5.5-6.0; mean [SD] score, 5.6 [0.6]) for descriptive questions and 6.0 (IQR, 5.9-6.0; mean [SD] score, 5.8 [0.8]) for binary questions (*P* = .10 determined by Mann-Whitney *U* test). Among both descriptive and binary questions, the median accuracy scores for easy, medium, and hard answers were 6.0 (IQR, 6.0-6.0; mean [SD] score, 5.9 [0.4]), 6.0 (IQR, 5.5-6.0; mean [SD] score, 5.6 [1.0]), and 6.0 (IQR, 5.5-6.0; mean [SD] score, 5.6 [0.1]), respectively (*P* = .07 determined by Kruskal-Wallis test). There was good interrater agreement (weighted κ = 0.6; *P* < .001) for accuracy and moderate agreement (weighted κ = 0.5; *P* < .001) for completeness (eTable 7 in Supplement 1).

### Total Analysis

Among all AI-generated answers (n = 284) from all 3 data sets (not including regraded answers), the median accuracy score was 5.5 (IQR, 4.0-6.0; mean [SD] score, 4.8 [1.6]), and the median completeness score was 3.0 (IQR, 2.0-3.0; mean [SD] score, 2.5 [0.7]) (Table 2; eTable 3 in Supplement 1). The median accuracy score was 5.0 (IQR, 3.4-6.0; mean [SD] score, 4.7 [1.6]) for all descriptive questions and 6.0 (IQR, 4.0-6.0; mean [SD] score, 4.9 [1.6]) for binary questions (*P* = .07 determined by Mann-Whitney *U* test). Among descriptive questions, the median accuracy scores for easy, medium, and hard questions were 5.2 (IQR, 3.0-6.0; mean [SD] score, 4.8 [1.5]), 5.5 (IQR, 3.3-6.0; mean [SD] score, 4.7 [1.7]), and 5.0 (IQR, 3.6-6.0; mean [SD] score, 4.5 [1.6,]), respectively (*P* = .40 determined by Kruskal-Wallis test) (Figure 2D). Among binary questions, the median accuracy scores for easy, medium, and hard questions were 6.0 (IQR, 5.0-6.0; mean [SD] score, 5.3 [1.5]), 5.5 (IQR, 3.4-6.0; mean [SD] score, 4.6 [1.6]), and 5.5 (IQR, 4.0-6.0; mean [SD] score, 4.8 [1.6]), respectively, which resulted in a significant difference among groups (*P* = .03 determined by Kruskal-Wallis test) (Figure 2E).

## Discussion

This study indicates that 3 months into its existence, chatbot has promise for providing accurate and comprehensive medical information. However, it remains well short of being completely reliable. The large multispecialty analyses (consisting of 180 questions) provided by more than 30 physicians across diverse specialties revealed that more than 50% were rated as “nearly all correct” or “completely correct.” Most answers were also rated as comprehensive as well. The median accuracy scores were generally higher than the mean scores, reflecting the multiple instances in which the chatbot was spectacularly and surprisingly wrong. Thus, using the current version of the chatbot for medical knowledge dissemination is not advisable and requires considering its potential to hallucinate by confidently delivering completely mistaken conclusions. However, these highly erroneous answers appeared to dramatically diminish over time.

Overall, accuracy was fairly high across question types and difficulty. Subjectively more difficult questions seemed to have slightly less accurate scores (mean score, 4.2) than easier questions (mean score, 4.6), suggesting a potential limitation in handling complex medical queries, but this did not reach statistical significance. The results for type of question (descriptive or binary) or difficulty level were broadly similar, implying that chatbot could have promise for open-ended question types with varying levels of difficulty, providing broad applicability.

Chatbot showed significant improvement over a short period of time (8-17 days). Compared with the median accuracy score of 2.0 (IQR, 1.0-2.0; mean [SD] score, 1.6 [0.5]) for the original low-quality answers, the median accuracy score improved to 4.0 (*P* < .001) (eTable 3 in Supplement 1). The common condition and melanoma or immunotherapy data sets, which were scored later than the multispecialty data sets, also had higher median scores. This clear trend for improvement could be attributed to the continuous update and refinement of algorithms and repetitive user feedback through reinforcement learning. To assess performance and consistency with more updated models in this rapidly moving field, the follow-up analysis of version 4 showed significant improvement compared with version 3.5 approximately 90 days prior. Twenty-four original answers (77.4%) that received accuracy scores lower than 6.0 improved. This may be attributed to the larger and more diverse training data set enabling it to better capture the nuances and complexities of medical terminology and concepts. These findings highlight the importance of regularly updating and refining AI models to stay current with the latest advances in NLP, and they highlight the quickly changing nature of this field.

This study demonstrates the potential of AI systems in answering nonmultiple choice clinical questions. However, unlike many other fields, the practice of medicine cannot rely on a tool that occasionally provides incorrect answers, even if such instances are rare. There are several challenges to overcoming the inaccuracies of chatbot-provided medical information. The model lacks a definitive source of truth. It is unable to grade the reliability of the sources of its training data, such as choosing established guidelines or PubMed-indexed articles over a social media blog discussing the same medical concept. Supervised training itself may mislead the model depending on the human supervisor’s knowledge limitations or biases. Slight variations in input phrasing can impact accuracy, leading to disparate responses. The model does not ask clarifying questions for ambiguous queries, instead relying on guessing user intentions. There are also transparency concerns because chatbot provides inaccurate citations on requests for sources.

With further validation, these tools could become valuable resources for rapid medical information retrieval in fast-paced clinical settings to enhance health care efficiency and complex decision-making. They can also be useful in creating personalized patient education information tailored to a given language and health literacy level. Health care professionals should also consider how patients may use these tools and how chatbot provides appropriate referrals to qualified health professionals. Training on proper use is imperative. Medical education should include training on the benefits and risks to ensure that both health care professionals and patients make informed decisions about when or how to use AI. At the same time, relying on current, publicly available versions of AI engines as the sole source of medical information is not advisable. If trained by reliable experts and with vetted medical information (eg, medical literature, pharmacology databases, and electronic medical records), LLMs have the potential to rapidly improve and transform the dissemination of medical knowledge. A recently released LLM trained exclusively on biomedical literature shows promise of domain-specific LLMs in health care.^13^

Further research is needed to validate the reliability of AI-provided medical information with large groups of health care professionals and diverse question types, assess its evolution over time, and address ethical, transparency, data security and privacy, and medicolegal concerns. Efforts should be made to incorporate reliable medical information sources, ensure comprehensive data inclusion (more up-to-date training data sets, figures, or tabular information), and establish nimble though robust standards and regulations for safe and effective implementation in health care.

### Limitations

Despite promising results, the scope of our conclusions is limited due to the modest sample size, single-center analysis, and the data set of 284 questions generated by 33 physicians, which may not be representative of all medical specialties and the many questions posed within them. The selection bias of physicians in academic practice and respondent bias were present. We also acknowledge the limitations of a scale that grades the degree of accuracy, which can introduce ambiguity within a medical context that necessitates strictly accurate information. Other limitations include the subjective choice of questions and the self-reported ratings, which may have introduced bias; similar judgements may also vary by physician (as the difference between more correct than incorrect vs nearly all correct [4.0 vs 5.0] may be small). Physicians chose questions with clear, uncontroversial answers based on current guidelines, which may not represent queries made by patients and the general public. Limited to 1 AI model, these findings may not apply to others, particularly with medical-specific training.

## Conclusion

While the chatbot-generated answers displayed high accuracy and completeness scores across various specialties, question types, and difficulty levels in this cross-sectional study, further development is needed to improve the reliability and robustness of these tools before clinical integration. Medical professionals and patients should recognize the limitations, should use extreme caution, and should actively verify AI-generated information with trusted sources. This study establishes an evidence base for using LLM in health care and highlights the importance of ongoing evaluation and regulation.

## Notes

Supplement 1. **eTable 1.** Physician Specialists Included in the Multispecialty Analysis **eTable 2.** Common Medical Conditions **eTable 3.** Accuracy and Completeness Scores for AI-Generated Answers to Medical Questions **eTable 4.** Reevaluation of Incorrect AI-Generated Answers **eTable 5.** Interrater Agreement Analysis–Accuracy of Melanoma and Immunotherapy Data Set **eTable 6.** Reevaluation of AI-Generated Answers With GPT-4: Melanoma/Immunotherapy Question Set **eTable 7.** Interrater Agreement Analysis–Accuracy of Common Medical Conditions Data Set
eAppendix.

Supplement 2.
Data Sharing Statement
