# Supplementary material for: Accuracy and Reliability of Chatbot Responses to Physician Questions
Source: JAMA Netw Open. 2023 Oct 2;6(10):e2336483. doi: 10.1001/jamanetworkopen.2023.36483 (PMC10546234; doi:10.1001/jamanetworkopen.2023.36483)
Supplement: Supplement 1. — eTable 1. Physician Specialists Included in the Multispecialty Analysis eTable 2. Common Medical Conditions eTable 3. Accuracy and Completeness Scores for AI-Generated Answers to Medical Questions eTable 4. Reevaluation of Incorrect AI-Generated Answers eTable 5. Interrater Agreement Analysis–Accuracy of Melanoma and Immunotherapy Data Set eTable 6. Reevaluation of AI-Generated Answers With GPT-4: Melanoma/Immunotherapy Question Set eTable 7. Interrater Agreement Analysis–Accuracy of Common Medical Conditions Data Set eAppendix. [file jamanetwopen-e2336483-s001.pdf]

# Supplemental Online Content

Goodman RS, Patrinely JR, Stone CA Jr. Accuracy and reliability of chatbot responses to physician questions. *JAMA Netw Open*. 2023;6(10):e2336483. doi:10.1001/jamanetworkopen.2023.36483

**eTable 1.** Physician Specialists Included in the Multispecialty Analysis
**eTable 2.** Common Medical Conditions
**eTable 3.** Accuracy and Completeness Scores for AI-Generated Answers to Medical Questions
**eTable 4.** Reevaluation of Incorrect AI-Generated Answers
**eTable 5.** Interrater Agreement Analysis–Accuracy of Melanoma and Immunotherapy Data Set
**eTable 6.** Reevaluation of AI-Generated Answers With GPT-4: Melanoma/Immunotherapy Question Set
**eTable 7.** Interrater Agreement Analysis–Accuracy of Common Medical Conditions Data Set
**eAppendix.**

This supplemental material has been provided by the authors to give readers additional information about their work.

© 2023 Goodman RS et al. *JAMA Network Open.*

**eTable 1**. Physician Specialists Included in the Multispecialty Analysis

| Specialty | Physician Count |
|---|---|
| Anesthesiology | 1 |
| Cardiology | 3 |
| Dermatology | 2 |
| Endocrinology | 1 |
| Gastroenterology | 2 |
| Hematology/Oncology | 5 |
| Infectious Disease | 2 |
| Neurological Surgery | 1 |
| Neurology | 1 |
| Ophthalmology | 2 |
| Pediatric Oncology | 1 |
| Pulmonary and Critical Care | 2 |
| Radiation Oncology | 1 |
| Rheumatology | 1 |
| Surgical Oncology | 6 |
| Trauma Surgery | 1 |
| Urology | 1 |
| **Total** | **33** |

© 2023 Goodman RS et al. *JAMA Network Open.*

**eTable 2**. Common Medical Conditions

| **Common Medical Conditions** |
|---|
| Diabetes |
| Hypertension |
| Depression |
| Urinary tract infection |
| Gastroesophageal reflux disease |
| Cough |
| Community acquired pneumonia |
| Acute coronary syndrome |
| Chronic obstructive pulmonary disease |
| Sepsis |

© 2023 Goodman RS et al. *JAMA Network Open.*

**eTable 3**. Accuracy and Completeness Scores for AI-Generated Answers to Medical Questions

| | | | | Question Type | | | | Question Difficulty | | | | | |
|---|---|---|---|---|---|---|---|---|---|---|---|---|---|
| | | Overall | | Accuracy | | Completeness | | Easy | | Medium | | Hard | |
| | | Accuracy | Completeness | Descriptive | Binary | Descriptive | Binary | Accuracy | Completeness | Accuracy | Completeness | Accuracy | Completeness |
| Multispecialty, n=180 | Median | 5.0 | 3.0 | 5.0 | 5.0 | 2.0 | 3.0 | 5.0 | 3.0 | 5.0 | 3.0 | 5.0 | 2.5 |
| | Mean | 4.4 | 2.4 | 4.3 | 4.5 | 2.3 | 2.6 | 4.6 | 2.6 | 4.3 | 2.4 | 4.2 | 2.4 |
| | SD | 1.7 | 0.7 | 1.7 | 1.7 | 0.8 | 0.6 | 1.7 | 0.7 | 1.7 | 0.7 | 1.8 | 0.7 |
| | IQR | 5.0 | 1.0 | 3.0 | 3.0 | 1.0 | 1.0 | 3.0 | 1.0 | 3.0 | 1.0 | 3.8 | 1.0 |
| Oncology, n=44 | Median | 6.0 | 3.0 | 6.0 | 6.0 | 3.0 | 3.0 | 6.0 | 3.0 | 5.5 | 2.8 | 5.8 | 3.0 |
| | Mean | 5.2 | 2.6 | 5.1 | 5.4 | 2.6 | 3.0 | 5.9 | 3.0 | 4.8 | 2.2 | 5.3 | 2.8 |
| | SD | 1.3 | 0.8 | 1.5 | 1.2 | 0.7 | 0.0 | 0.3 | 0.1 | 1.7 | 0.2 | 1.1 | 0.5 |
| | IQR | 1.0 | 0.5 | 1.0 | 1.0 | 1.0 | 0.0 | 0.0 | 0.0 | 2.1 | 1.6 | 1.0 | 0.1 |
| Common Conditions, n=60 | Median | 6.0 | 3.0 | 6.0 | 6.0 | 3.0 | 3.0 | 6.0 | 3.0 | 6.0 | 3.0 | 5.8 | 3.0 |
| | Mean | 5.7 | 2.8 | 5.6 | 5.8 | 2,7 | 2.8 | 5.9 | 2.9 | 5.6 | 2.7 | 5.6 | 2.8 |
| | SD | 0.7 | 0.5 | 0.6 | 0.8 | 0.6 | 0.5 | 0.4 | 0.2 | 1.0 | 0.6 | 0.1 | 0.5 |
| | IQR | 0.5 | 0.0 | 0.5 | 0.1 | 1.0 | 0.0 | 0.0 | 0.0 | 0.5 | 0.5 | 0.5 | 0.4 |
| All, n=284 | Median | 5.5 | 3.0 | 5.0 | 6.0 | 3.0 | 3.0 | 6.0 | 3.0 | 5.5 | 3.0 | 5.0 | 3.0 |
| | Mean | 4.8 | 2.5 | 4.7 | 4.9 | 2.4 | 2.7 | 5.0 | 2.7 | 4.7 | 2.4 | 4.6 | 2.5 |
| | SD | 1.6 | 0.7 | 1.6 | 1.6 | 0.7 | 0.5 | 1.5 | 0.1 | 1.7 | 0.8 | 1.6 | 0.1 |
| | IQR | 2.0 | 1.0 | 2.6 | 2.0 | 1.0 | 0.0 | 1.0 | 0.5 | 2.6 | 1.0 | 2.0 | 1.0 |

Abbreviations: SD, Standard Deviation; IQR: Interquartile Range

© 2023 Goodman RS et al. *JAMA Network Open.*

The accuracy scale was a six-point Likert scale (1 – completely incorrect, 2 – more incorrect than correct, 3 – Approximately equal correct and incorrect, 4 – more correct than incorrect, 5 – nearly all correct, 6 – completely correct), and the completeness scale was a three-point Likert scale (1 – incomplete, addresses some aspects of the question, but significant parts are missing or incomplete, 2 – adequate, addresses all aspects of the question and provides the minimum amount of information required to be considered complete, 3 – comprehensive, addresses all aspects of the question and provides additional information or context beyond what was expected). Answers that were completely incorrect on the accuracy scale (score of 1) were not graded on comprehensiveness.

© 2023 Goodman RS et al. *JAMA Network Open.*

**eTable 4**. Reevaluation of Incorrect AI-Generated Answers

| | | Accuracy | |
|---|---|---|---|
| | | **Original** | **Rescored** |
| **Rescored**, n=8 | Median | 2.0 | 4.0 |
| | Mean | 1.6 | 3.9 |
| | SD | 0.5 | 1.8 |
| | IQR | 1.0 | 3.3 |

Abbreviations: SD, Standard Deviation; IQR: Interquartile Range
*Wilcoxon p<0.0001

© 2023 Goodman RS et al. *JAMA Network Open.*

**eTable 5**. Interrater Agreement Analysis–Accuracy of Melanoma and Immunotherapy Data Set

| **Accuracy Scores** | **1** | **2** | **3** | **4** | **5** | **6** | **Total** |
|---|---|---|---|---|---|---|---|
| **1** | 2 | 0 | 1 | 0 | 0 | 1 | 4 |
| **2** | 0 | 1 | 0 | 0 | 0 | 0 | 1 |
| **3** | 0 | 0 | 0 | 0 | 0 | 0 | 0 |
| **4** | 0 | 0 | 0 | 1 | 1 | 2 | 4 |
| **5** | 0 | 0 | 0 | 0 | 1 | 2 | 3 |
| **6** | 0 | 0 | 1 | 2 | 5 | 24 | 32 |
| **Total** | 2 | 1 | 2 | 3 | 7 | 29 | 44 |

*weighted kappa, quadratic weighting = 0.7, p=<0.01

© 2023 Goodman RS et al. *JAMA Network Open.*

**eTable 6**. Reevaluation of AI-Generated Answers With GPT-4: Melanoma/Immunotherapy Question Set

| | | Accuracy | |
|---|---|---|---|
| | | Original | Rescored |
| **Rescored**, n=44 | Median | 6.0 | 6.0 |
| | Mean | 5.2 | 5.7 |
| | SD | 1.5 | 0.8 |
| | IQR | 1.0 | 0.0 |

Abbreviations: SD, Standard Deviation; IQR: Interquartile Range
*Wilcoxon p=0.003
Original: 2/1/23, rescored: 5/2/23

© 2023 Goodman RS et al. *JAMA Network Open.*

**eTable 7**. Interrater Agreement Analysis–Accuracy of Common Medical Conditions Data Set

| **Accuracy Scores** | **1** | **2** | **3** | **4** | **5** | **6** | **Total** |
|---|---|---|---|---|---|---|---|
| **1** | 0 | 1 | 0 | 0 | 0 | 0 | 1 |
| **2** | 0 | 0 | 0 | 0 | 0 | 0 | 0 |
| **3** | 0 | 0 | 0 | 1 | 0 | 0 | 1 |
| **4** | 0 | 0 | 0 | 0 | 1 | 2 | 3 |
| **5** | 0 | 0 | 0 | 1 | 0 | 6 | 7 |
| **6** | 0 | 0 | 0 | 0 | 7 | 41 | 48 |
| **Total** | 0 | 1 | 0 | 2 | 8 | 49 | 60 |

*weighted kappa, quadratic weighting = 0.7, p<0.01

© 2023 Goodman RS et al. *JAMA Network Open.*

**eAppendix.**

| Question Set - Physician Specialty - Question Type - Question Difficulty | Physician-generated Question | AI-generated Answer | Accuracy Rating | Completeness Rating |
|---|---|---|---|---|
| Multispecialty - Hematology/Oncology - Descriptive - Easy | What are the first line treatments for metastatic melanoma? | The first-line treatment options for metastatic melanoma typically include targeted therapies, immunotherapies, and chemotherapy. These treatments are often used in combination.<br>1. Targeted therapies: These medications target specific molecules involved in the growth and spread of cancer cells. Examples include vemurafenib (Zelboraf), dabrafenib (Tafinlar), and trametinib (Mekinist).<br>2. Immunotherapies: These treatments help to boost the immune system's ability to fight cancer cells. Examples include nivolumab (Opdivo), pembrolizumab (Keytruda), and ipilimumab (Yervoy).<br>3. Chemotherapy: This treatment uses drugs to kill cancer cells. Examples of chemotherapy drugs that may be used to treat metastatic melanoma include dacarbazine (DTIC) and temozolomide (Temodar).<br>It is important to note that the most appropriate treatment for an individual with metastatic melanoma will depend on a variety of factors, including the location and size of the cancer, the patient's overall health, and their preference. A healthcare provider can provide more specific recommendations based on the individual's situation. | 4 | 2 |
| Multispecialty - Hematology/Oncology - Descriptive - Medium | What patients are eligible for adjuvant therapy for high-risk resected melanoma? | Adjuvant therapy is treatment given to patients who have undergone surgery to remove cancerous tumors, in order to reduce the risk of cancer recurrence. It is typically used for patients with high-risk resected melanoma, which is melanoma that has been surgically removed, but is at high risk of returning or spreading.<br>Eligibility for adjuvant therapy for high-risk resected melanoma generally depends on the stage of the cancer at the time of surgery and other prognostic factors. Some specific factors that may be considered when determining eligibility for adjuvant | 5 | 3 |

© 2023 Goodman RS et al. *JAMA Network Open.*

| | | | | |
|---|---|---|---|---|
| | | therapy include:<br>1. Tumor thickness: Patients with melanoma that is greater than 1 mm in thickness at the time of surgery are generally considered to be at high risk for recurrence and may be eligible for adjuvant therapy.<br>2. Ulceration: Patients with ulcerated melanoma at the time of surgery are generally considered to be at high risk for recurrence and may be eligible for adjuvant therapy.<br>3. Lymph node involvement: Patients with melanoma that has spread to the lymph nodes at the time of surgery are generally considered to be at high risk for recurrence and may be eligible for adjuvant therapy.<br>4. Distant metastasis: Patients with melanoma that has spread to distant organs or tissues at the time of surgery are generally considered to be at high risk for recurrence and may be eligible for adjuvant therapy.<br>5. Age: Older patients (typically over the age of 70) may be less likely to benefit from adjuvant therapy due to other health issues and may not be eligible for this treatment.<br>In general, patients with high-risk resected melanoma are more likely to be eligible for adjuvant therapy if they have any of the above prognostic factors, as these factors increase the risk of cancer recurrence. A healthcare provider can provide more specific recommendations based on the individual's situation. | | |
| Multispecialty - Hematology/Oncology - Descriptive - Hard | What are possible treatment options for patients who have failed anti-PD-1 therapy for metastatic melanoma without BRAF mutations? | Anti-PD-1 therapy is a type of immunotherapy that targets a protein called PD-1 on immune cells. It is used to treat melanoma and other types of cancer. If a patient with metastatic melanoma without BRAF mutations has failed anti-PD-1 therapy, there are several treatment options that may be considered:<br>1. Targeted therapies: These medications target specific molecules involved in the growth and spread of cancer cells. Examples include MEK inhibitors and PI3K inhibitors.<br>2. Immunotherapies: Other immunotherapies that target different pathways may be effective in patients who have failed anti-PD-1 therapy. Examples include CTLA-4 inhibitors and IDO inhibitors.<br>3. Chemotherapy: This treatment uses drugs to kill cancer cells. Examples of chemotherapy drugs that may be used to treat melanoma include dacarbazine (DTIC) and temozolomide (Temodar).<br>4. Radiotherapy: This treatment uses high-energy beams, such as | 5 | 3 |

© 2023 Goodman RS et al. *JAMA Network Open.*

| | | | | |
|---|---|---|---|---|
| | | X-rays, to kill cancer cells. It is often used to treat cancer that has spread to the brain or spine.<br>5. Clinical trials: There may be clinical trials evaluating new treatments for melanoma that are currently recruiting patients. Enrolling in a clinical trial may provide access to treatments that are not yet widely available.<br>It is important to note that the most appropriate treatment for an individual with metastatic melanoma will depend on a variety of factors, including the location and size of the cancer, the patient's overall health, and their preference. A healthcare provider can provide more specific recommendations based on the individual's situation. | | |
| Multispecialty - Hematology/Oncology - Binary - Easy | Is ulceration a negative prognostic indicator in melanoma? | Ulceration is considered a negative prognostic indicator in melanoma. The presence of ulceration on a primary melanoma lesion is associated with a higher risk of metastasis and poorer survival outcomes. According to the American Joint Committee on Cancer (AJCC) melanoma staging system, the presence of ulceration on a primary melanoma lesion is a significant prognostic factor and is used to classify the tumor as thicker (T2a) regardless of its Breslow depth.<br>Several studies have shown that the presence of ulceration on a primary melanoma lesion is associated with a higher risk of regional and distant metastases, as well as poor overall survival. One study by (A.J. Stratigos, et al. Journal of the American Academy of Dermatology, 2003) found that the presence of ulceration was associated with a 2.5-fold increased risk of death from melanoma, independent of other known prognostic factors such as thickness and lymph node involvement.<br>Another study by (K.A. Marzuka, et al. Cancer, 2010) found that the presence of ulceration on a primary melanoma lesion was associated with a higher risk of distant metastasis and poor survival outcomes, even in patients with thin primary melanoma (less than 1mm in thickness).<br>It's important to note that other factors such as thickness, mitotic rate, lymph node involvement, and subtype of melanoma also play a role in determining prognosis and treatment plan. | 6 | 3 |

| | | | | |
|---|---|---|---|---|
| Multispecialty - Hematology/Oncology - Binary - Medium | Should a patient with a 2.1mm ulcerated melanoma have a sentinel lymph node biopsy? | The decision to perform a sentinel lymph node biopsy (SLNB) in a patient with a 2.1mm ulcerated melanoma should be made on an individualized basis and should take into account the patient's overall health and the specific characteristics of their melanoma.<br>The National Comprehensive Cancer Network (NCCN) guidelines for Melanoma recommend SLNB for patients with primary melanoma that is greater than 1mm in thickness, regardless of the presence of ulceration. The American Joint Committee on Cancer (AJCC) melanoma staging system also recommends SLNB for patients with primary melanoma that is greater than 1mm in thickness, regardless of the presence of ulceration.<br>That being said, the presence of ulceration on the primary melanoma lesion is considered a negative prognostic factor, and is associated with a higher risk of regional and distant metastasis, as well as poor overall survival. Therefore, some physicians may consider SLNB for a 2.1mm ulcerated melanoma as a way to further assess the risk of lymph node involvement and to guide adjuvant treatment decisions.<br>It's important to note that the decision to perform SLNB should be made by a multidisciplinary team of professionals including an oncologist, a surgical oncologist, and a pathologist, after careful consideration of all the patient's clinical, pathological and imaging data. | 6 | 3 |
| Multispecialty - Hematology/Oncology - Binary - Hard | Is adjuvant radiation indicated for a 3.5 mm non-ulcerated melanoma that is removed with negative margins? | Adjuvant radiation therapy is not typically indicated for a 3.5 mm non-ulcerated melanoma that is removed with negative margins.<br>The National Comprehensive Cancer Network (NCCN) guidelines for Melanoma do not recommend the use of adjuvant radiation therapy for patients with primary melanoma that is 3.5 mm or less in thickness and has negative margins. This is because the risk of recurrence is considered low in these patients and the benefits of radiation therapy may not outweigh the potential risks and side effects.<br>It is worth mentioning that the American College of Radiology (ACR) and the American Society for Radiation Oncology (ASTRO) have released guidelines for the management of cutaneous melanoma, which states that "Radiation therapy is not recommended for the treatment of melanoma at any stage"<br>It's important to note that these are general guidelines, and the treatment plan for each patient should be individualized based on their overall health and the specific characteristics of their | 6 | 3 |

© 2023 Goodman RS et al. *JAMA Network Open.*

| | | | | |
|---|---|---|---|---|
| | | melanoma. Medical professionals should consult the most recent version of the guidelines for the most up-to-date information. | | |
| Multispecialty - Hematology/Oncology - Descriptive - Easy | What surgery is done for esophageal cancer: | There are several surgical options for treating esophageal cancer, including: Esophagectomy: This is the most common surgery for esophageal cancer. It involves removing the cancerous portion of the esophagus, as well as some surrounding healthy tissue and lymph nodes. Depending on the location and extent of the cancer, the surgery may be performed through an open incision or using minimally invasive techniques. Endoscopic mucosal resection (EMR): EMR is a type of endoscopic surgery that removes the cancerous tissue by cutting it away. This procedure is typically used for early-stage esophageal cancers that are confined to the mucosa (inner lining) of the esophagus. Stent placement: A stent is a small, metal mesh tube that is placed in the esophagus to help keep it open. This procedure is typically used for patients who have a tumor that is blocking the esophagus and making it difficult to swallow. Photodynamic therapy: Photodynamic therapy is a procedure that uses a special light and medication to kill cancer cells. This procedure is typically used for early-stage esophageal cancers that are confined to the mucosa. It is important to note that the specific surgical procedure that is best for a particular patient will depend on many factors, including the stage and location of the cancer, as well as the patient's overall health and preferences. | 5 | 3 |
| Multispecialty - Hematology/Oncology - Descriptive - Medium | What is the cure rate for stage 4 esophageal cancer? | The cure rate for stage 4 esophageal cancer is generally considered to be low. The 5-year survival rate for stage 4 esophageal cancer is estimated to be between 10% and 20% but it can vary from study to study, and even within the same study, depending on patient's characteristics, treatment received, and the type of esophageal cancer. It is important to note that survival rate statistics are based on data from the past and may not reflect the most current treatment options, so it's always best to consult with your oncologist and medical team to have a clear understanding of the survival rate and treatment options in your specific case. | 5 | 3 |

© 2023 Goodman RS et al. *JAMA Network Open.*

| | | | | |
|---|---|---|---|---|
| Multispecialty - Hematology/Oncology - Descriptive - Hard | Are there any clinical trials for treatment of advanced esophageal cancer? | Yes, there are clinical trials for the treatment of advanced esophageal cancer. These trials are designed to test new treatments, such as new drugs or new combinations of existing drugs, to see if they are more effective than the current standard of care. Some examples of clinical trials for advanced esophageal cancer include: Immunotherapy: Immunotherapy trials are testing drugs that help the immune system recognize and attack cancer cells. For example, a trial is testing the combination of the immunotherapy drug nivolumab and the chemotherapy drug cisplatin in patients with advanced esophageal cancer. Targeted therapy: Targeted therapy trials are testing drugs that target specific molecules that help cancer cells grow and spread. For example, a trial is testing the drug Lenvatinib in combination with the chemotherapy drug cisplatin in patients with advanced esophageal cancer. Chemotherapy: Some trials are testing new combinations of chemotherapy drugs to see if they are more effective than existing treatment options. For example, a trial is testing the combination of the drugs Paclitaxel, Cisplatin and 5-Fluorouracil in patients with advanced esophageal cancer. Radiotherapy: Some trials are testing new techniques in radiotherapy such as proton therapy and intensity-modulated radiation therapy (IMRT) to see if they are more effective than existing treatment options. As a language model, I do not have access to a live database of current clinical trials. However, there are some clinical trials for esophageal cancer that are or have been conducted in the past, such as: CheckMate-577: This is a phase III clinical trial that is testing the combination of the immunotherapy drug nivolumab and the chemotherapy drug cisplatin in patients with advanced esophageal cancer. KEYNOTE-181: This is a phase III clinical trial that is testing the use of the immunotherapy drug pembrolizumab as a first-line treatment for patients with advanced esophageal cancer. IMpower131: This is a phase III clinical trial that is testing the combination of the immunotherapy drug atezolizumab and the chemotherapy drug cisplatin in patients with advanced esophageal cancer. REVEL: This is a phase III clinical trial that is testing the use of the targeted therapy drug Lenvatinib in combination with the chemotherapy drug cisplatin in patients with advanced esophageal cancer. OPTIMIZE: This is a phase II/III trial that is testing a new combination of Paclitaxel, Cisplatin and 5- | 5 | 3 |

© 2023 Goodman RS et al. *JAMA Network Open.*

| | | | | |
|---|---|---|---|---|
| | | Fluorouracil in patients with advanced esophageal cancer. Proton therapy: This trial is testing the effectiveness of proton therapy as a treatment option for esophageal cancer. Intensity-modulated radiation therapy (IMRT): This trial is testing the effectiveness of IMRT as a treatment option for esophageal cancer. It's important to note that these trials may not be currently open for recruitment and that new trials may have been initiated after my knowledge cut-off. | | |
| Multispecialty - Hematology/Oncology - Binary - Easy | Can you do surgery to treat esophageal cancer? | Yes, surgery can be used to treat esophageal cancer. The type of surgery used will depend on the stage and location of the cancer. Options may include esophagectomy (removal of part or all of the esophagus), lymph node removal, and reconstruction of the esophagus. Surgery may be performed alone or in combination with other treatments such as chemotherapy or radiation therapy. | 5 | 3 |
| Multispecialty - Hematology/Oncology - Binary - Medium | Should a patient with stage 4 esophageal cancer get surgery? | No, typically surgery is not recommended for stage 4 esophageal cancer because the cancer has spread to other organs and surgery alone is unlikely to cure the cancer. Patients with stage 4 esophageal cancer may receive palliative care to improve symptoms and quality of life, rather than curative treatment. However, it is important to note that each patient's case is different and that a patient's treatment plan should be discussed with a multidisciplinary team of oncologists and surgeons who can evaluate the patient's specific case and provide personalized recommendations. | 5 | 3 |
| Multispecialty - Hematology/Oncology - Binary - Hard | Do you give chemoradiotherapy to a patient with squamous cell cancer of the esophagus who has metastatic disease? | Yes, chemoradiotherapy is one of the treatment options that can be considered for a patient with squamous cell cancer of the esophagus who has metastatic disease. This combination of chemotherapy and radiation therapy may be used to shrink the tumor and improve symptoms, but it is not typically curative. Other palliative care options can also be considered, such as supportive care, which is designed to improve the patient's quality of life, and/or radiation therapy. It's important to note that different patients respond differently to treatment, and the best treatment option will depend on the patient's individual situation, including the extent of the cancer, the patient's overall health, and the patient's preferences. | 5 | 3 |

© 2023 Goodman RS et al. *JAMA Network Open.*

| | | | | |
|---|---|---|---|---|
| Multispecialty - Opthalmology - Descriptive - Easy | What is the first line of treatment for patients with neovascular age-related macular degeneration? | The first line of treatment for neovascular age-related macular degeneration (AMD) is typically anti-vascular endothelial growth factor (VEGF) therapy, which targets the abnormal blood vessels that form in the retina. This can include intravitreal injections of medications such as ranibizumab, aflibercept, or bevacizumab. These medications are typically administered on a regular schedule, such as every 4-8 weeks, to maintain the effects of the treatment. According to the American Academy of Ophthalmology, treatment should be initiated as soon as possible after diagnosis to prevent vision loss. | 6 | 3 |
| Multispecialty - Opthalmology - Descriptive - Medium | What are established risk factors for primary open angle glaucoma? | Primary open angle glaucoma (POAG) is a chronic and progressive optic neuropathy characterized by the gradual loss of retinal ganglion cells and their axons, which leads to visual field loss and blindness. Some established risk factors for POAG include: Age: POAG is more common in older adults, especially those over 60 years old. Family history: POAG tends to run in families, and having a family history of the condition increases the risk. Ethnicity: POAG is more common in people of African descent, and they also tend to develop the condition at a younger age. High intraocular pressure (IOP): Elevated IOP is a major risk factor for POAG. Optic disc characteristics: Some people are more likely to develop POAG if they have certain characteristics of the optic disc, such as a thinner or more cupped optic disc. Systemic hypertension: High blood pressure is a risk factor for POAG. Hypermetropia: Long-sighted people are more susceptible to POAG. Cardiovascular disease: POAG might be related to cardiovascular disease. The American Academy of Ophthalmology recommends regular eye exams for people at increased risk of POAG, including African Americans over 40, and everyone over 60, regardless of race or family history. Regular check-ups are crucial for early detection and management of POAG. | 4 | 2 |
| Multispecialty - Opthalmology - Descriptive - Hard | What are the risks and benefits of potential treatment options for a pseudophakic patient with a superior macula-on retinal | A pseudophakic patient with a superior macula-on retinal detachment with one retinal break and a posterior vitreous detachment is a serious condition that requires prompt treatment to prevent vision loss. The two main treatment options for this condition are pneumatic retinopexy and scleral buckle surgery. Pneumatic retinopexy is a less invasive procedure that involves injecting a gas bubble into the eye to push the detached retina back into place. The benefits of this treatment include less surgical | 4 | 1 |

© 2023 Goodman RS et al. *JAMA Network Open.*

| | | | | |
|---|---|---|---|---|
| | detachment with one retinal break and a posterior vitreous detachment? | trauma, a quicker recovery, and the ability to perform the procedure on an outpatient basis. However, there is a risk of the gas bubble not being able to hold the retina in place, and additional surgery may be needed. Scleral buckle surgery involves placing a silicone band around the eye to push the detached retina back into place. The benefits of this treatment include a higher success rate in reattaching the retina, and a lower risk of redetachment. However, the procedure requires a general anesthetic, a longer recovery time, and a higher risk of complications such as infection, cataract formation and retinal detachment in the other eye. The American Academy of Ophthalmology recommends considering the patient's condition, preferences, and the surgeon's experience when selecting the appropriate treatment option. A consultation with an ophthalmologist who is an expert in retina surgery is recommended to help make the best decision for the individual case. | | |
| Multispecialty - Opthalmology - Binary - Easy | Is patching an appropriate initial treatment for amblyopia? | Patching is an appropriate initial treatment for amblyopia, also known as "lazy eye". Amblyopia is a vision disorder in which the eye and brain do not work together properly, leading to poor vision in one eye. Patching involves covering the "good" eye with a patch or glasses with a frosted lens to force the brain to use the "lazy" eye. This helps to strengthen the visual connections in the brain and improve vision in the affected eye. The American Academy of Ophthalmology recommends that children with amblyopia should be treated as early as possible, ideally before the age of 6. The recommended duration of patching is usually 2-6 hours per day, depending on the severity of the amblyopia, and the child's age. The treatment may need to be continued for several months or even years. It's important to note that patching alone is not always effective and sometimes other treatments such as corrective lenses, eye drops or even surgery are needed in combination with patching. An ophthalmologist should be consulted for the best treatment plan. | 6 | 3 |

© 2023 Goodman RS et al. *JAMA Network Open.*

| | | | | |
|---|---|---|---|---|
| Multispecialty - Opthalmology - Binary - Medium | Is vitrectomy indicated for a patient with post-cataract endophthalmitis and light-perception visual acuity? | The American Academy of Ophthalmology recommends that vitrectomy is the first-line surgical treatment for post-cataract endophthalmitis and LPVA, as it can prevent further vision loss, and provide the best chance of visual recovery. Vitrectomy should be performed as soon as possible after the diagnosis of endophthalmitis, ideally within 24-48 hours. | 6 | 2 |
| Multispecialty - Opthalmology - Binary - Hard | Is pan-retinal photocoagulation indicated in a patient with proliferative diabetic retinopathy and neovascularization of the optic disc and vitreous hemorrhage? | Pan-retinal photocoagulation (PRP) is indicated in a patient with proliferative diabetic retinopathy (PDR) and neovascularization of the optic disc and vitreous hemorrhage. PDR is a complication of diabetes that affects the retina and is characterized by the growth of new, fragile blood vessels that can lead to severe vision loss. PRP is a laser treatment that aims to destroy these new blood vessels and prevent further vision loss. PRP involves the use of a laser to create small burns in the peripheral retina. This causes the abnormal blood vessels to be blocked, and reduces the risk of vitreous hemorrhage. Neovascularization of the optic disc is one of the most severe forms of PDR and it is associated with poor visual outcomes, thus, PRP is considered an important treatment in this scenario. The American Academy of Ophthalmology recommends PRP as a primary treatment for PDR with neovascularization of the optic disc and vitreous hemorrhage. The treatment should be initiated as soon as possible, as the earlier it's performed the greater the chances of success. The goal of PRP is to preserve or improve visual function and to reduce the risk of severe visual loss. | 5 | 2 |
| Multispecialty - Cardiology - Descriptive - Easy | What are all the possible oral anticoagulants for a patient with atrial fibrillation? | There are several oral anticoagulants that can be used in patients with atrial fibrillation, including: Warfarin: This is a vitamin K antagonist that has been the traditional treatment for atrial fibrillation, but it requires frequent monitoring of the international normalized ratio (INR) and dietary restrictions. Dabigatran: This is a direct thrombin inhibitor that does not require monitoring of the INR or dietary restrictions. However, it has a specific dosing guideline and it should be avoided in case of renal impairment. Rivaroxaban: This is a direct factor Xa inhibitor that does not require monitoring of the INR or dietary restrictions. However, it should be avoided in case of renal impairment. Apixaban: This is a direct factor Xa inhibitor that does not require monitoring of the INR or dietary restrictions. However, it should be avoided in case | 5 | 3 |

© 2023 Goodman RS et al. *JAMA Network Open.*

| | | | | |
|---|---|---|---|---|
| | | of renal impairment. Edoxaban: This is a direct factor Xa inhibitor that does not require monitoring of the INR or dietary restrictions. However, it should be avoided in case of renal impairment. It is important to note that guidelines regarding the use of these anticoagulants may vary depending on the country and the specific population of patients. | | |
| Multispecialty - Cardiology - Descriptive - Medium | What patients with atrial fibrillation are not eligible to use novel oral anticoagulants for stroke prevention? | Patients with atrial fibrillation who are not eligible to use novel oral anticoagulants (NOACs) for stroke prevention include those with: Severe renal impairment (CrCl < 30 mL/min) as the use of NOACs may increase the risk of bleeding in these patients. Active bleeding or a high risk of bleeding as NOACs can increase the risk of bleeding. Recent major surgery or trauma as NOACs can increase the risk of bleeding. Concomitant use of drugs that interact with NOACs and increase the risk of bleeding. Uncontrolled hypertension, as it can increase the risk of bleeding. It is important to note that these criteria may vary depending on the country and the specific population of patients. | 6 | 1 |
| Multispecialty - Cardiology - Descriptive - Hard | What are the possible anticoagulant and antiplatelet options in patients who have atrial fibrillation and undergone percutaneous coronary intervention with stenting for acute coronary syndrome? | In patients with atrial fibrillation who have undergone percutaneous coronary intervention (PCI) with stenting for acute coronary syndrome, the following anticoagulant and antiplatelet options are possible: Dual antiplatelet therapy (DAPT) with aspirin and a P2Y12 inhibitor (such as clopidogrel, prasugrel, or ticagrelor) for at least 12 months following the stenting procedure. Triple therapy with a vitamin K antagonist (such as warfarin), aspirin and a P2Y12 inhibitor (such as clopidogrel, prasugrel, or ticagrelor) for at least 3-6 months following the stenting procedure. Oral anticoagulant and antiplatelet therapy (NOACs) such as Dabigatran, Rivaroxaban, Apixaban, Edoxaban in combination with aspirin for at least 12 months following the stenting procedure. | 6 | 2 |
| Multispecialty - Cardiology - Binary - Easy | Should patients with atrial fibrillation who have a $CHAD_2S_2$-VASc score of 4 be placed on anti-coagulants? | Patients with atrial fibrillation who have a CHA2DS2-VASc score of 4 are considered to be at high risk of stroke and should be placed on anticoagulants. The CHA2DS2-VASc score is a tool used to assess the risk of stroke in patients with atrial fibrillation. It assigns 1 point for each of the following risk factors: congestive heart failure, hypertension, age 75 or older, diabetes, stroke or TIA (transient ischemic attack) history, and vascular disease (such as peripheral artery disease or aortic plaque). A score of 2 or higher | 6 | 3 |

| | | | | |
|---|---|---|---|---|
| | | is considered to be associated with an increased risk of stroke. In the United States, the American College of Cardiology/American Heart Association/Heart Rhythm Society (ACC/AHA/HRS) recommends that oral anticoagulants be considered for stroke prevention in patients with atrial fibrillation and a CHA2DS2-VASc score of 2 or higher. In patients with a CHA2DS2-VASc score of 4, the risk of stroke is considered to be high and therefore, oral anticoagulants are strongly recommended. | | |
| Multispecialty - Cardiology - Binary - Medium | Should repeated pulmonary vein isolation/atrial fibrillation catheter-ablation procedures be performed in a patient with atrial fibrillation recurrence if symptoms were improved after the initial pulmonary vein isolation/ atrial fibrillation catheter-ablation procedure? | Yes, repeated pulmonary vein isolation/atrial fibrillation catheter-ablation procedures can be performed in a patient with atrial fibrillation recurrence if symptoms were improved after the initial pulmonary vein isolation/ atrial fibrillation catheter-ablation procedure, but it will depend on the patient's symptoms and the potential risks and benefits of the procedure. The decision to perform repeated pulmonary vein isolation/atrial fibrillation (PVI/AF) catheter-ablation procedures in a patient with atrial fibrillation recurrence depends on the patient's symptoms and the potential risks and benefits of the procedure. PVI/AF catheter-ablation is a procedure that uses radiofrequency energy to create small lesions in the heart tissue around the pulmonary veins in order to isolate them and prevent the initiation and maintenance of atrial fibrillation. The procedure is considered to be safe and effective in many patients with AF, but it is not without risks. Repeat ablation procedure might be considered if the recurrence of atrial fibrillation is having a significant negative impact on the patient's quality of life or if the patient is intolerant to or contraindicated for oral anticoagulation therapy. In the United States, the American College of Cardiology/American Heart Association (ACC/AHA) have guidelines stating that repeat PVI/AF catheter ablation procedure can be considered in patients with atrial fibrillation recurrence who have symptoms that are not well controlled with medications and if the recurrence of atrial fibrillation is having a significant negative impact on the patient's quality of life. | 6 | 3 |

© 2023 Goodman RS et al. *JAMA Network Open.*

| | | | | |
|---|---|---|---|---|
| Multispecialty - Cardiology - Binary - Hard | Should atrial fibrillation catheter- ablation procedures be used as first line therapy in patients with tachycardia-induced cardiomyopathy? | No, Atrial fibrillation catheter ablation procedures should not be used as first line therapy in patients with tachycardia-induced cardiomyopathy. Tachycardia-induced cardiomyopathy is a condition where prolonged rapid heart rate causes left ventricular dysfunction and heart failure symptoms. The primary management of tachycardia-induced cardiomyopathy is the control of the underlying tachycardia. This could involve drugs, ablation, or a combination of both. The American College of Cardiology/American Heart Association (ACC/AHA) guidelines on the management of tachycardia-induced cardiomyopathy recommend that the first step in the management of tachycardia-induced cardiomyopathy should be rate control, either with medications or a pacemaker, and that ablation should be considered as a second-line therapy after rate control has failed or is not feasible. | 3 | 2 |
| Multispecialty - Rheumatology - Descriptive - Easy | What is the preferred urate lower therapy for the treatment of gout? | The preferred urate-lowering therapy for the treatment of gout is the use of xanthine oxidase inhibitors (such as allopurinol) or uricosuric agents (such as probenecid). These medications work by decreasing the production of uric acid or increasing its excretion, respectively, in order to lower the levels of uric acid in the blood and prevent gout attacks. Other options for urate-lowering therapy include febuxostat (a non-purine selective inhibitor of xanthine oxidase) and pegloticase (a recombinant enzyme that breaks down uric acid). It is important to note that the choice of medication and dosage will depend on the individual patient's medical history and other factors, and should be determined by a healthcare professional. | 5 | 3 |
| Multispecialty - Rheumatology - Descriptive - Medium | What is the first line immunosuppressive therapy for patients with rheumatoid arthritis and high disease activity? | The first line immunosuppressive therapy for patients with rheumatoid arthritis (RA) and high disease activity is typically a disease-modifying antirheumatic drug (DMARD) such as methotrexate. Methotrexate is a widely-used DMARD that has been shown to be effective in reducing inflammation and improving symptoms in patients with RA. Other first line DMARDs that may be used to treat RA include leflunomide, sulfasalazine, and hydroxychloroquine. It is important to note that the choice of medication and dosage will depend on the individual patient's medical history and other factors, and should be determined by a healthcare professional. In some cases, a combination of DMARDs may be used to achieve the best possible results. | 6 | 3 |

© 2023 Goodman RS et al. *JAMA Network Open.*

| | | | | |
|---|---|---|---|---|
| Multispecialty - Rheumatology - Descriptive - Hard | What genetic test should be performed prior to starting an African American patient on allopurinol for gout management? | It is generally recommended that patients undergo genetic testing for the presence of the HLA-B*58:01 allele prior to starting treatment with allopurinol for gout management. This is because individuals who carry the HLA-B*58:01 allele are at increased risk of developing severe cutaneous adverse reactions, such as Stevens-Johnson syndrome and toxic epidermal necrolysis, in response to allopurinol. Testing for the presence of this allele can help healthcare providers determine the appropriate dosage and monitor for potential adverse reactions in individuals who are African American or of African descent, as they are more likely to carry the allele than individuals of other racial or ethnic groups. | 5 | 3 |
| Multispecialty - Rheumatology - Binary - Easy | Is methotrexate contraindicated for rheumatoid arthritis in patients with end stage renal disease? | Yes, methotrexate is generally contraindicated in patients with end stage renal disease (ESRD). This is because methotrexate is eliminated from the body primarily through the kidneys, and patients with ESRD may not be able to adequately clear the drug from their system. In addition, methotrexate can accumulate in the body of patients with ESRD, leading to an increased risk of toxic effects such as bone marrow suppression and liver damage. For these reasons, it is generally not recommended to use methotrexate in patients with ESRD or other severe kidney dysfunction. | 6 | 3 |
| Multispecialty - Rheumatology - Binary - Medium | Should a patient with rheumatoid arthritis and a positive hepatitis C antibody receive antiviral prophylaxis prior to receiving rituximab? | Yes, it is generally recommended that a patient with rheumatoid arthritis who has a positive hepatitis C antibody receive antiviral prophylaxis prior to receiving rituximab. This is because rituximab can reactivate latent hepatitis C virus (HCV) infection and cause a flare of hepatitis. Antiviral prophylaxis with a regimen such as pegylated interferon and ribavirin can help reduce the risk of HCV reactivation and prevent the potential for severe liver damage. | 4 | 2 |
| Multispecialty - Rheumatology - Binary - Hard | Should a patient with high disease activity from rheumatoid arthritis and a recent history nontuberculous mycobacterial lung infection receive a tumor necrosis | No, a patient with high disease activity from rheumatoid arthritis and a recent history of nontuberculous mycobacterial lung infection should not receive a tumor necrosis factor (TNF) inhibitor as first line biologic DMARD therapy. TNF inhibitors, such as etanercept, infliximab, and adalimumab, are commonly used to treat patients with RA who have not responded to other DMARDs. However, these medications can increase the risk of infections, including serious and potentially life-threatening infections caused by nontuberculous mycobacteria. For this reason, TNF inhibitors are generally not recommended for patients who have a recent | 4 | 2 |

© 2023 Goodman RS et al. *JAMA Network Open.*

| | | | | |
|---|---|---|---|---|
| | factor inhibitor as first line biologic DMARD therapy? | history of nontuberculous mycobacterial infection or who are at high risk of developing such infections. Other biologic DMARDs, such as rituximab or tocilizumab, may be safer options in these cases and should be considered as first line therapy. | | |
| Multispecialty - Pediatric Oncology - Descriptive - Easy | What is the NCI definition for high risk B cell pediatric acute lymphoblastic leukemia? | The National Cancer Institute (NCI) defines high-risk B-cell pediatric acute lymphoblastic leukemia (ALL) as a subtype of ALL that is characterized by specific genetic or molecular features that increase the risk of treatment failure or relapse. These features include the presence of the Philadelphia chromosome (t(9;22) or the BCR-ABL1 fusion gene), high white blood cell count at diagnosis, and poor response to initial treatment. High-risk B-cell ALL is considered to have a higher chance of recurring or relapsing than other subtypes of ALL, and requires more aggressive treatment. | 3 | 1 |
| Multispecialty - Pediatric Oncology - Descriptive - Medium | What are some first line treatment options for B cell pediatric acute lymphoblastic leukemia? | First-line treatment options for B-cell pediatric acute lymphoblastic leukemia (ALL) typically involve a combination of chemotherapy and targeted therapy, and may include radiation therapy and immunotherapy. The standard regimen for B-cell ALL is called the "Berlin-Frankfurt-Munster (BFM) protocol" which includes multi-agent chemotherapy, including vincristine, daunorubicin, and prednisone. The therapy also involves L-asparaginase and intrathecal therapy (delivering chemotherapy directly into the cerebrospinal fluid) to prevent central nervous system (CNS) involvement. In addition to chemotherapy, targeted therapy such as tyrosine kinase inhibitors (TKI) may be administered in certain cases. Radiation therapy is also a key component of the treatment plan for high-risk ALL, it may be delivered to the brain and spinal cord to prevent or treat central nervous system (CNS) involvement. Some of the other treatment options includes monoclonal antibodies such as blinatumomab and inotuzumab ozogamicin that target the cancer cells specifically. | 5 | 2 |

© 2023 Goodman RS et al. *JAMA Network Open.*

| | | | | |
|---|---|---|---|---|
| Multispecialty - Pediatric Oncology - Descriptive - Hard | What are some treatment options for relapsed B cell pediatric acute lymphoblastic leukemia? | Treatment options 5for relapsed B-cell pediatric acute lymphoblastic leukemia (ALL) may include: 1. Chemotherapy: High-dose chemotherapy with or without stem cell transplantation may be used to treat relapsed ALL.1. Chemotherapy: High-dose chemotherapy with or without stem cell transplantation may be used to treat relapsed ALL. 2. Targeted therapy: Monoclonal antibodies such as blinatumomab and inotuzumab ozogamicin that target the cancer cells specifically may be used in relapsed ALL. 3. Immunotherapy: CAR-T cell therapy is another option used to treat relapsed B-cell ALL. 4. Clinical trials: Participating in clinical trials for new treatments may be an option for relapsed B-cell ALL. It is important to note that the treatment options for relapsed B-cell ALL are complex and the decision about the best treatment will depend on the specific subtype of ALL, the patient's age, underlying health conditions and other factors. | 6 | 3 |
| Multispecialty - Pediatric Oncology - Binary - Easy | Is minimal residual disease at end of induction a defined prognostic factor for B cell pediatric acute lymphoblastic leukemia? | Minimal residual disease (MRD) at the end of induction therapy is considered a prognostic factor for B-cell pediatric acute lymphoblastic leukemia (ALL). MRD refers to the presence of cancer cells that remain in the body after initial treatment. The level of MRD at the end of induction therapy has been found to be a strong predictor of relapse in B-cell ALL. Children with a high level of MRD at the end of induction therapy have a higher risk of relapse compared to those with a low level of MRD. MRD assessment is routinely used in the management of children with B-cell ALL and it is used in determining the duration of maintenance therapy and risk-adapted therapy. It is important to note that different institutions may have different cut-offs for MRD positivity and that treatment protocols may vary accordingly. | 5 | 2 |
| Multispecialty - Pediatric Oncology - Binary - Medium | Is central nervous system radiation a component of all upfront therapy for B cell pediatric acute lymphoblastic leukemia? | Central nervous system (CNS) radiation is not a component of all upfront therapy for B-cell pediatric acute lymphoblastic leukemia (ALL). CNS radiation is typically only used in cases where the cancer has spread to the brain and spinal cord. The standard approach for preventing CNS relapse in B-cell ALL is intrathecal chemotherapy, which is the delivery of chemotherapy directly into the cerebrospinal fluid (CSF) through a lumbar puncture. This is done in order to prevent leukemia cells from hiding in the brain and spinal cord, which is a common site of relapse in ALL. However, some protocols may include radiotherapy as a part of the upfront treatment if the patient has a high risk of CNS relapse. The decision to include radiotherapy in the upfront treatment will | 6 | 3 |

© 2023 Goodman RS et al. *JAMA Network Open.*

| | | | | |
|---|---|---|---|---|
| | | depend on patient specific factors such as risk group, age, underlying health conditions, and other factors. | | |
| Multispecialty - Pediatric Oncology - Binary - Hard | Is chimeric antigen receptor T cell therapy (CAR-T) an option for refractory, second or later line relapse therapy for B cell pediatric acute lymphoblastic leukemia? | Chimeric antigen receptor T cell therapy (CAR-T) is an option for refractory, second or later-line relapse therapy for B-cell pediatric acute lymphoblastic leukemia (ALL). CAR-T therapy is a form of immunotherapy that involves removing T cells from a patient's blood, genetically modifying them to produce CARs, and infusing them back into the patient. CAR-T cells target and kill cancer cells that express a specific protein. It is an option for patients with relapsed or refractory B-cell ALL who have failed other treatment options. The FDA has approved two CAR-T therapies, tisagenlecleucel and brexucabtagene autoleucel, for the treatment of relapsed or refractory B-cell ALL in children and young adults. It's important to note that CAR-T therapy is a relatively new treatment option and it is still being studied for use in pediatric ALL. | 6 | 3 |
| Multispecialty - Urology - Descriptive - Easy | A 75 year old man with a 50 pack year smoking history and gross hematuria should undergo what evaluation? | A 75-year-old man with a 50 pack-year smoking history and gross hematuria (blood in the urine) should undergo: Urinalysis, urine culture, CT scan or MRI of the urinary tract-Cytoscopy if appropriate. Consider bladder cancer evaluation as per NCCN guideline | 3 | 2 |
| Multispecialty - Urology - Descriptive - Medium | What patients should not undergo cis-platin based neoadjuvant chemotherapy for muscle invasive bladder cancer? | Patients with significant renal impairment (creatinine clearance <30 ml/min) or severe neuropathy should not undergo cisplatin-based neoadjuvant chemotherapy for muscle-invasive bladder cancer according to the National Comprehensive Cancer Network (NCCN) guidelines. | 6 | 1 |

© 2023 Goodman RS et al. *JAMA Network Open.*

| | | | | |
|---|---|---|---|---|
| Multispecialty - Urology - Descriptive - Hard | What are advantages and disadvantages for trimodal therapy for invasive bladder cancer? | Trimodal therapy for invasive bladder cancer is a combination of surgery, radiation therapy, and chemotherapy. Advantages include: -Increased chance of complete tumor removal. -Decreased risk of tumor recurrence -Improved overall survival. Disadvantages include: -Increased risk of complications from the combination of treatments -Longer recovery time -Higher cost. It is important to note that trimodal therapy is not suitable for all patients and it should be individualized based on patient's overall health and the stage of cancer, according to the National Comprehensive Cancer Network (NCCN) guidelines. | 1 | 1 |
| Multispecialty - Urology - Binary - Easy | Should a 55 year old smoker with gross hematuria undergo a cystoscopy? | Yes, a 55-year-old smoker with gross hematuria should undergo a cystoscopy as it is an important diagnostic tool that allows the visualization of the bladder and urethra, which can help determine the cause of the hematuria and guide further treatment. This is recommended by the National Comprehensive Cancer Network (NCCN) guidelines. | 6 | 1 |
| Multispecialty - Urology - Binary - Medium | For patient with bulky retroperitoneal nodes and testicular pure seminoma, is four cycles of BEP chemotherapy an appropriate adjuvant treatment? | Yes, four cycles of BEP (bleomycin, etoposide, cisplatin) chemotherapy is an appropriate adjuvant treatment for a patient with bulky retroperitoneal nodes and testicular pure seminoma. This is in line with the guidelines from the European Society for Medical Oncology (ESMO) and the American Society of Clinical Oncology (ASCO) | 1 | 1 |
| Multispecialty - Urology - Binary - Hard | Should a 58 year old man with a Gleason 6 (grade group 1) prostate cancer with a PSA of 22 and a T1c digital rectal examination undergo a CT scan? | No, a CT scan may not be necessary for a 58-year-old man with a Gleason 6 (grade group 1) prostate cancer with a PSA of 22 and a T1c digital rectal examination. According to the National Comprehensive Cancer Network (NCCN) guidelines, a CT scan should be considered in men with a high-risk prostate cancer, such as a PSA level >20 ng/mL, a gleason score >7, or cT3-T4 stage. In this case, the patient has a PSA of 22 and a low-risk prostate cancer, so a CT scan may not be necessary, but it's ultimately up to the treating physician and the patient to decide based on the risk-benefit ratio and patient's preferences. | 2 | 2 |

© 2023 Goodman RS et al. *JAMA Network Open.*

| | | | | |
|---|---|---|---|---|
| Multispecialty - Hematology/Oncology - Descriptive - Easy | What are the first line treatments for metastatic pancreas adenocarcinoma? | The first line treatment for metastatic pancreatic adenocarcinoma typically includes a combination of chemotherapy and targeted therapy. According to the National Comprehensive Cancer Network (NCCN) guidelines, the recommended first-line chemotherapy regimen for metastatic pancreatic adenocarcinoma is a combination of gemcitabine and nab-paclitaxel. Targeted therapy with an anti-VEGF agent such as bevacizumab may also be added to this regimen. Additionally, the European Society for Medical Oncology (ESMO) also recommends the use of FOLFIRINOX (a combination of 5-fluorouracil, leucovorin, irinotecan, and oxaliplatin) as a first-line treatment option for metastatic pancreatic adenocarcinoma in patients who are fit enough to tolerate the treatment. It is important to note that the treatment plan should be individualized based on the patient's condition, performance status, and other factors. | 3 | 2 |
| Multispecialty - Hematology/Oncology - Descriptive - Medium | What patients are eligible for atezolizumab and bevacizumab for first line treatment in metastatic hepatocellular carcinoma? | Atezolizumab and bevacizumab are FDA-approved as first-line treatment options for patients with advanced or metastatic hepatocellular carcinoma (HCC) in combination with the tyrosine kinase inhibitor (TKI) Lenvatinib. According to the National Comprehensive Cancer Network (NCCN) guidelines for hepatocellular carcinoma, patients who have been diagnosed with advanced or metastatic HCC and who have an ECOG performance status of 0 or 1 are eligible for this treatment regimen. Patients with Child-Pugh class A liver function and no extrahepatic spread are eligible for this treatment as well. Patients with a history of prior systemic therapy, viral hepatitis infection, and previous liver disease are also eligible for treatment with atezolizumab and bevacizumab in combination with Lenvatinib. The combination of atezolizumab and bevacizumab with Lenvatinib is recommended for patients with intermediate or poor risk HCC according to the Barcelona Clinic Liver Cancer (BCLC) classification. It is important to note that before starting the treatment, a comprehensive evaluation of the patient's medical history and a thorough examination of the patient's liver function should be done to confirm the eligibility of the patient for the treatment. Furthermore, it is also important to regularly monitor the patient's condition and adjust the treatment plan if necessary. | 1 | N/A |

© 2023 Goodman RS et al. *JAMA Network Open.*

| | | | | |
|---|---|---|---|---|
| Multispecialty - Hematology/Oncology - Descriptive - Hard | What are the possible treatment options for chemorefractory MSS, RAS mutated metastatic colon cancer? | Treatment options for chemorefractory microsatellite stable (MSS), RAS-mutated metastatic colon cancer are limited and may include targeted therapies, immunotherapy, and clinical trials. According to the National Comprehensive Cancer Network (NCCN) guidelines, targeting the RAS-RAF-MEK-ERK pathway with therapies such as MEK inhibitors (e.g. Binimetinib, Selumetinib) and combination of MEK and RAF inhibitors (e.g. Regorafenib) may be considered in patients with RAS-mutated colon cancer that has progressed on standard chemotherapy. Immunotherapy options such as anti-PD-1/PD-L1 checkpoint inhibitors (e.g. Pembrolizumab, Durvalumab) are also recommended as a treatment option for patients with chemorefractory MSS, RAS-mutated colon cancer, either as single agent or in combination with other therapies. Additionally, clinical trials are also an option for patients with chemorefractory MSS, RAS-mutated colon cancer, as there are ongoing studies evaluating novel agents and combination therapies for this patient population. It is worth noting that the best treatment plan should be tailored to the individual patient, taking into account the patient's overall health status, comorbidities, and other factors, and it should be discussed with an oncologist experienced in the management of colon cancer. | 2 | 1 |
| Multispecialty - Hematology/Oncology - Binary - Easy | Is adjuvant therapy recommended for resected hepatocellular carcinoma? | Yes, adjuvant therapy is recommended for resected hepatocellular carcinoma (HCC) in certain cases. According to the National Comprehensive Cancer Network (NCCN) guidelines for hepatocellular carcinoma, adjuvant therapy is recommended for patients with high-risk HCC who have undergone curative intent resection. High-risk HCC is defined as the presence of multiple or larger tumors, or the presence of extrahepatic spread at the time of diagnosis. The recommended adjuvant therapy for high-risk HCC is a combination of transarterial chemoembolization (TACE) and Sorafenib, an oral multi-kinase inhibitor. The treatment plan should be tailored to the individual patient, taking into account the patient's overall health status, comorbidities, and other factors, and it should be discussed with an oncologist experienced in the management of HCC. | 1 | N/A |

© 2023 Goodman RS et al. *JAMA Network Open.*

| | | | | |
|---|---|---|---|---|
| Multispecialty - Hematology/Oncology - Binary - Medium | Should a patient with MSI-H T4N0 colon cancer with multiple high risk pathologic features receive adjuvant chemotherapy with 5FU and oxaliplatin? | Yes, a patient with MSI-H T4N0 colon cancer with multiple high-risk pathologic features should receive adjuvant chemotherapy with 5-Fluorouracil (5-FU) and Oxaliplatin. According to the National Comprehensive Cancer Network (NCCN) guidelines for colon cancer, patients with MSI-H (Microsatellite Instability-High) colon cancer, which is characterized by a high level of genetic instability, have a high risk of recurrence and a poorer prognosis. Therefore, it is recommended that they receive adjuvant therapy with combination chemotherapy. Patients with T4N0 colon cancer are considered high-risk patients as they have a primary tumor that has grown through the wall of the colon and invaded nearby organs or structures, but no lymph node involvement. In addition, high-risk pathologic features such as perineural invasion and lymphovascular invasion are associated with a higher risk of recurrence and poorer outcomes. Therefore, the combination of 5-FU and Oxaliplatin is recommended as adjuvant therapy for this patient population. It is important to note that the treatment plan should be tailored to the individual patient, taking into account the patient's overall health status, comorbidities, and other factors, and it should be discussed with an oncologist experienced in the management of colon cancer. | 4 | 1 |
| Multispecialty - Hematology/Oncology - Binary - Hard | Should a patient with resected intestinal type ampullary adenocarcinoma receive gemcitabine-based adjuvant chemotherapy? | Yes, a patient with resected intestinal type ampullary adenocarcinoma should receive gemcitabine-based adjuvant chemotherapy. According to the National Comprehensive Cancer Network (NCCN) guidelines for ampullary carcinoma, the recommended adjuvant therapy for patients with resected intestinal type ampullary adenocarcinoma is a combination of gemcitabine and capecitabine. This is based on the evidence from clinical trials that have shown that the combination of gemcitabine and capecitabine improved disease-free survival and overall survival in patients with resected intestinal type ampullary adenocarcinoma. It is worth noting that the treatment plan should be tailored to the individual patient, taking into account the patient's overall health status, comorbidities, and other factors, and it should be discussed with an oncologist experienced in the management of ampullary carcinoma. | 2 | 1 |

© 2023 Goodman RS et al. *JAMA Network Open.*

| | | | | |
|---|---|---|---|---|
| Multispecialty - Cardiology - Descriptive - Easy | What are first line classes of medications for essential hypertension? | The first line classes of medications for essential hypertension (high blood pressure) include: Angiotensin-converting enzyme inhibitors (ACE inhibitors), Angiotensin receptor blockers (ARBs), Calcium channel blockers (CCBs), Diuretics. These medications are recommended as first-line therapy by various guidelines, such as the American College of Cardiology (ACC) and American Heart Association (AHA) 2017 Guideline for the Prevention, Detection, Evaluation, and Management of High Blood Pressure in Adults. | 5 | 3 |
| Multispecialty - Cardiology - Descriptive - Medium | What type of initial therapy should be used for blood pressure lowering in an individual with stage 1 hypertension without clinical atherosclerotic cardiovascular disease and an estimated 10-year atherosclerotic cardiovascular disease risk based on the pooled cohort equation of 9%? | In an individual with stage 1 hypertension without clinical atherosclerotic cardiovascular disease and an estimated 10-year atherosclerotic cardiovascular disease risk based on the pooled cohort equation of 9%, the American College of Cardiology (ACC) and American Heart Association (AHA) 2017 Guideline for the Prevention, Detection, Evaluation, and Management of High Blood Pressure in Adults recommends lifestyle modification as initial therapy for blood pressure lowering. This includes dietary changes such as the DASH diet, weight management, regular physical activity, and limiting alcohol consumption. It's important to note that the decision of starting pharmacological treatment should be individualized and should take into account patient's overall cardiovascular risk, preferences and values. | 6 | 3 |
| Multispecialty - Cardiology - Descriptive - Hard | If screening for primary hyperaldosteronism without medication adjustment reveals elevated plasma renin and borderline elevated aldosterone levels, what class of medications | If screening for primary hyperaldosteronism without medication adjustment reveals elevated plasma renin and borderline elevated aldosterone levels, medications that can affect the renin-angiotensin-aldosterone system (RAAS) such as ACE inhibitors, ARBs and Aldosterone antagonists should be withheld prior to retesting. The Endocrine Society guidelines for the diagnosis and management of primary aldosteronism recommend that these medications should be stopped at least four weeks prior to confirmatory testing for primary aldosteronism. | 5 | 3 |

© 2023 Goodman RS et al. *JAMA Network Open.*

| | | | | |
|---|---|---|---|---|
| | should be withheld prior to retesting? | | | |
| Multispecialty - Cardiology - Binary - Easy | Does pre-eclampsia increase future cardiovascular disease risk? | Pre-eclampsia, a pregnancy-specific complication characterized by hypertension and proteinuria, has been associated with an increased risk of future cardiovascular disease (CVD). According to the American College of Obstetricians and Gynecologists (ACOG) guidelines, women with a history of pre-eclampsia have an increased risk of hypertension, stroke, and myocardial infarction later in life, particularly if they had severe pre-eclampsia or multiple episodes of pre-eclampsia. In addition to the ACOG guidelines, The American Heart Association (AHA) also recognizes that pre-eclampsia is a risk factor for future CVD, and recommends that women with a history of pre-eclampsia be screened and treated for hypertension, diabetes, and dyslipidemia, and be counseled about lifestyle changes to reduce their risk of CVD. It's important to note that the risk of CVD is not only limited to women who had pre-eclampsia but also to the baby born from the pre-eclamptic pregnancy, thus long-term follow-up and monitoring is crucial. | 6 | 3 |
| Multispecialty - Cardiology - Binary - Medium | Should a 65 year old patient with a low density lipoprotein cholesterol level of 191 mg/dL be started on a medium intensity statin for primary prevention of atherosclerotic cardiovascular disease? | No. A 65 year old patient with a low-density lipoprotein cholesterol (LDL-C) level of 191 mg/dL should not be started on a medium intensity statin for primary prevention of atherosclerotic cardiovascular disease (ASCVD) based on the LDL-C level alone. The decision to initiate statin therapy for primary prevention of ASCVD depends on the patient's overall cardiovascular risk. The American College of Cardiology (ACC) and American Heart Association (AHA) provide guidelines for the management of blood cholesterol to reduce ASCVD risk. According to the ACC/AHA guideline, for primary prevention in patients without clinical ASCVD, a statin therapy should be considered if the patient has an estimated 10-year ASCVD risk of 7.5% or higher. The risk can be estimated by using the Pooled Cohort Equations (PCE) which consider age, sex, race, total cholesterol, HDL cholesterol, systolic blood pressure, treatment for hypertension, diabetes status, and smoking status. Therefore, the patient's 10- | 2 | 2 |

© 2023 Goodman RS et al. *JAMA Network Open.*

| | | | | |
|---|---|---|---|---|
| | | year risk of ASCVD should be calculated and if the patient's 10-year risk of ASCVD is 7.5% or higher, a medium-intensity statin therapy (such as atorvastatin 40 mg/day or rosuvastatin 20 mg/day) should be considered, otherwise if the patient has a lower 10-year risk of ASCVD, then a lower intensity statin or non-statin therapy should be considered. It's worth noting that the decision to start statin therapy should be individualized, taking into account the patient's overall cardiovascular risk, preferences, and values, and should be done in consultation with a healthcare provider. | | |
| Multispecialty - Cardiology - Binary - Hard | Should a 66-year-old male with a history of coronary artery disease with a prior myocardial infarction and diabetes mellitus on atorvastatin 80 mg daily be started next on ezetimibe or a PCSK9 inhibitor like alirocumab for LDL cholesterol reduction? | Yes. A 66-year-old male with a history of coronary artery disease, prior myocardial infarction, and diabetes mellitus on atorvastatin 80 mg daily may be started on ezetimibe or a PCSK9 inhibitor like alirocumab for LDL cholesterol reduction. According to the American College of Cardiology (ACC) and American Heart Association (AHA) guidelines on the management of blood cholesterol to reduce ASCVD risk, patients with clinical ASCVD such as prior myocardial infarction and diabetes mellitus, who are on maximally tolerated statin therapy and still have LDL cholesterol level greater than 70 mg/dL, should be considered for additional LDL-C lowering therapy. Ezetimibe and PCSK9 inhibitors are the two classes of drugs that can be added to statin therapy for this purpose, and the choice of medication will depend on the patient's preference, cost, and potential side effects, and should be done in consultation with a healthcare provider. It's important to note that the decision to add additional LDL-C lowering therapy should be individualized taking into account patient's overall cardiovascular risk, preferences, and values. | 4 | 2 |
| Multispecialty - Surgical Oncology - Descriptive - Easy | What are the first line adjuvant treatment options for patients with resected colon cancer? | For patients with resected colon cancer, the first line adjuvant treatment options typically include: 5-fluorouracil (5-FU) plus leucovorin, which is given as a continuous infusion or as a bolus injection, often in combination with radiation therapy. Oxaliplatin, irinotecan, or capecitabine, which can be given in combination with 5-FU and leucovorin. Bevacizumab, a monoclonal antibody that targets vascular endothelial growth factor, is another option that can be added to the above regimens. Treatment recommendations for colon cancer are determined by the stage of the cancer, as well as the patient's overall health. It's important for patients to discuss their treatment options with their oncologist and to consider clinical trial participation. National Comprehensive | 2 | 2 |

© 2023 Goodman RS et al. *JAMA Network Open.*

| | | | | |
|---|---|---|---|---|
| | | Cancer Network (NCCN) Guidelines v.3.2021 is a good source for the treatment recommendations in colon cancer. | | |
| Multispecialty - Surgical Oncology - Descriptive - Medium | Which patients with rectal cancer should be considered for total neoadjuvant therapy? | Patients with cT3 or cT4 rectal cancer, or cN+ rectal cancer (cancer that has spread to nearby lymph nodes) should be considered for total neoadjuvant therapy (TNT). This is according to the National Comprehensive Cancer Network (NCCN) guidelines for rectal cancer. TNT is a treatment approach that involves administering chemotherapy and radiation therapy before surgery, with the goal of shrinking the tumor and making it easier to remove. It may also help reduce the risk of the cancer returning after surgery. | 6 | 3 |
| Multispecialty - Surgical Oncology - Descriptive - Hard | Which patients with colorectal cancer with peritoneal carcinomatosis are candidates for cytoreductive surgery and hyperthermic intraperitoneal chemotherapy? | Patients with colorectal cancer with peritoneal carcinomatosis who are candidates for cytoreductive surgery (CRS) and hyperthermic intraperitoneal chemotherapy (HIPEC) are those with a good performance status, minimal extra-abdominal disease, and a limited number of peritoneal tumors. This is according to the guidelines of the European Society of Medical Oncology (ESMO) and the Society of Surgical Oncology (SSO). CRS is a surgical procedure that aims to remove as much of the cancerous tissue as possible, while HIPEC is a form of chemotherapy that is administered directly into the peritoneal cavity during surgery. Together, CRS and HIPEC are used to treat patients with peritoneal carcinomatosis, which is a condition in which cancer cells have spread to the lining of the abdomen. It is important to note that the decision to administer CRS and HIPEC should be made by the patient's multidisciplinary team of healthcare professionals based on the patient's individual case, including the stage of cancer, overall health and personal preferences. | 5 | 2 |

© 2023 Goodman RS et al. *JAMA Network Open.*

| | | | | |
|---|---|---|---|---|
| Multispecialty - Surgical Oncology - Binary - Easy | Is adjuvant chemotherapy recommended after resection of stage III colon cancer? | Yes, adjuvant chemotherapy is recommended after resection of stage III colon cancer. According to the National Comprehensive Cancer Network (NCCN) guidelines, adjuvant chemotherapy is recommended for patients with stage III colon cancer (also known as locally advanced colon cancer) after surgical resection. The recommended regimens include FOLFOX (leucovorin, fluorouracil, and oxaliplatin) or capecitabine with oxaliplatin. The duration of treatment is typically 6 months. It's important to note that adjuvant chemotherapy after surgery is intended to improve survival by reducing the risk of recurrence and spread of cancer. | 6 | 3 |
| Multispecialty - Surgical Oncology - Binary - Medium | Should a patient with a clinical T3N1 mid-rectal cancer receive neoadjuvant chemoradiation therapy? | Yes, a patient with a clinical T3N1 mid-rectal cancer should receive neoadjuvant chemoradiation therapy. According to the National Comprehensive Cancer Network (NCCN) guidelines for rectal cancer, patients with cT3 or cT4 rectal cancer, or cN+ rectal cancer (cancer that has spread to nearby lymph nodes) should be considered for neoadjuvant chemoradiation therapy. In this case, the patient has a T3N1 mid-rectal cancer, meaning the tumor has grown through the muscular wall of the rectum and into the surrounding tissue, and cancer has spread to nearby lymph nodes, thus meeting the criteria for neoadjuvant chemoradiation therapy. | 6 | 3 |
| Multispecialty - Surgical Oncology - Binary - Hard | Is single stage resection indicated for a patient with an isolated 1.5cm superficial liver metastasis and a right colon primary tumor? | It depends. A single stage resection for a patient with an isolated 1.5cm superficial liver metastasis and a right colon primary tumor may be considered, but it depends on several factors such as the size, location, and number of liver metastases, as well as the patient's overall health and fitness for surgery. According to the guidelines of the European Society of Surgical Oncology (ESSO), the European Society of Medical Oncology (ESMO), the American Society of Clinical Oncology (ASCO), and the National Comprehensive Cancer Network (NCCN), a patient with a right colon primary tumor and a single, isolated and superficial liver metastasis (less than 3cm) may be considered for a simultaneous resection of both the primary tumor and the liver metastasis. However, this decision should be made by the patient's multidisciplinary team of healthcare professionals based on the patient's individual case, including the stage of cancer, overall health, and personal preferences. It is important to note that even if a patient meets these criteria, a simultaneous resection of the primary tumor and the liver metastasis is a complex surgery that | 6 | 3 |

© 2023 Goodman RS et al. *JAMA Network Open.*

| | | | | |
|---|---|---|---|---|
| | | carries a higher risk of complications and should be carefully considered by the patient and their surgical team. | | |
| Multispecialty - Allergy, Pulmonary, and Critical Care - Descriptive - Easy | What is the best ventilator strategy for acute respiratory distress syndrome? | The best ventilator strategy for acute respiratory distress syndrome (ARDS) is to use low tidal volumes (around 6 mL/kg of predicted body weight) and a high positive end-expiratory pressure (PEEP) level to minimize lung injury and prevent atelectrauma. The ARDS Network recommends using a lung-protective ventilation strategy for the management of ARDS. | 6 | 2 |
| Multispecialty - Allergy, Pulmonary, and Critical Care - Descriptive - Medium | What are the indications for non-invasive ventilation in acute respiratory failure? | The indications for non-invasive ventilation (NIV) in acute respiratory failure include: Type II respiratory failure (e.g. chronic obstructive pulmonary disease (COPD) exacerbation), Type III respiratory failure (e.g. heart failure), Acute asthma, Pneumonia, Obesity hypoventilation syndrome, The use of NIV should be considered in patients who are conscious, cooperative, and able to clear secretions, and have adequate gas exchange, hemodynamics, and no contraindications to NIV, such as the presence of a high level of consciousness disturbance, severe hypoxemia (PaO2 <60 mmHg), or a suspected or confirmed tension pneumothorax. It is important to note that NIV should not be used in patients with acute hypoxemic respiratory failure caused by acute respiratory distress syndrome (ARDS) or other forms of severe lung injury. It is also important to note that in some cases, NIV may be used as a bridge to invasive ventilation in patients who do not respond to NIV or if their condition deteriorates despite NIV. The guidelines such as the British Thoracic Society and National Institute for Health and Care Excellence (NICE) provide guidance on the use of NIV in acute respiratory failure. | 3 | 3 |

© 2023 Goodman RS et al. *JAMA Network Open.*

| | | | | |
|---|---|---|---|---|
| Multispecialty - Allergy, Pulmonary, and Critical Care - Descriptive - Hard | What is the best second line vasopressor for septic shock? | The best second-line vasopressor for septic shock is considered to be norepinephrine (noradrenaline) as it has been shown to improve survival in septic shock patients. Norepinephrine is a potent vasoconstrictor that increases the systemic vascular resistance, thus improving blood pressure and organ perfusion. According to the Surviving Sepsis Campaign Guidelines, norepinephrine is recommended as the first-choice vasopressor in septic shock patients who are not responding to initial fluid and/or vasopressor therapy. The guidelines recommend the use of norepinephrine as the primary vasopressor in septic shock to maintain a mean arterial pressure (MAP) of at least 65 mmHg, in order to improve organ perfusion and oxygenation. It is important to note that the use of other vasopressors, such as vasopressin or epinephrine, may be considered in some cases, depending on the patient's response to therapy and other clinical factors. And use of vasopressors should be guided by frequent monitoring of hemodynamics and blood pressure, and titrated to the individual patient's response. | 1 | 3 |
| Multispecialty - Allergy, Pulmonary, and Critical Care - Binary - Easy | Should mechanically ventilated patients with ARDS have daily spontaneous breathing trials? | Mechanically ventilated patients with ARDS should have daily spontaneous breathing trials (SBT) as per the current guideline recommendations. The purpose of SBT is to determine if the patient can tolerate breathing without the assistance of the ventilator, and if successful, the patient can be extubated and transitioned to non-invasive ventilation or discharged home. According to the ARDS network guidelines, patients with ARDS should have daily SBT, which consist of a trial of unassisted breathing with the ventilator set to assist-control mode with a low level of pressure support (typically 5-8 cm H2O) and PEEP, to assess the patient's ability to breathe without the ventilator. A successful SBT is defined as the patient being able to maintain a tidal volume of at least 5 ml/kg of predicted body weight, a respiratory rate of less than 35 breaths per minute, and no signs of distress or respiratory failure. It is important to note that the SBT should be performed in the presence of clinical stability, such as no signs of infection, hemodynamic stability, and stable oxygenation. And the SBT should be performed with close monitoring, including continuous pulse oximetry and capnography, and with a rapid response team available in case of failure of the trial. | 6 | 3 |

© 2023 Goodman RS et al. *JAMA Network Open.*

| | | | | |
|---|---|---|---|---|
| Multispecialty - Allergy, Pulmonary, and Critical Care - Binary - Medium | Should patients with sepsis with a low mean arterial pressure receive fluid resuscitation? | Patients with sepsis and a low mean arterial pressure (MAP) should receive fluid resuscitation as per the current guideline recommendations. The aim of fluid resuscitation in sepsis is to maintain an adequate perfusion pressure and organ perfusion. According to the Surviving Sepsis Campaign guidelines, patients with sepsis and a low MAP (less than 65 mmHg) should receive an initial fluid bolus of 30mL/kg of crystalloid (such as normal saline or lactated Ringer's solution) within the first 3 hours of presentation. The guidelines also recommend that patients should be reassessed for fluid responsiveness after the initial fluid bolus, and additional fluid boluses may be given as needed to maintain a MAP of at least 65 mmHg. It is important to note that fluid resuscitation should be guided by frequent monitoring of hemodynamics, such as heart rate, blood pressure, urine output, and central venous pressure, and titrated to the individual patient's response. And the use of vasopressors may also be needed to maintain a MAP of at least 65 mmHg in some cases, especially in patients who do not respond to fluid resuscitation. | 6 | 3 |
| Multispecialty - Allergy, Pulmonary, and Critical Care - Binary - Hard | Should adults with acute respiratory distress syndrome be ventilates with higher PEEP levels? | Adults with acute respiratory distress syndrome (ARDS) should be ventilated with higher positive end-expiratory pressure (PEEP) levels as per current guideline recommendations. Higher PEEP levels help to recruit collapsed alveoli and improve lung compliance, which can improve oxygenation and reduce the risk of lung injury. According to the ARDS Network guidelines, patients with ARDS should be ventilated using a lung-protective strategy, which involves using low tidal volumes (around 6 mL/kg of predicted body weight) and a high PEEP level. The guidelines recommend that the PEEP level should be titrated to achieve the highest level of PEEP that allows the patient to maintain a PaO2/FiO2 ratio of at least 200 mmHg. It is important to note that the use of higher PEEP levels should be guided by frequent monitoring of hemodynamics, oxygenation, and lung mechanics, and titrated to the individual patient's response. It is also important to note that the use of high PEEP may not be appropriate for all patients with ARDS, and the decision to use high PEEP levels should be made on a case-by-case basis, taking into consideration the patient's underlying lung pathology and other clinical factors. | 6 | 3 |

© 2023 Goodman RS et al. *JAMA Network Open.*

| | | | | |
|---|---|---|---|---|
| Multispecialty - Trauma Surgery - Descriptive - Easy | What is the best initial test for a patient with Zone II cervical gunshot wound without hard signs of vascular injury? | The best initial test for a patient with a Zone II cervical gunshot wound without hard signs of vascular injury would be a CT angiogram of the neck and brain. If this test does not show any vascular injuries, then the patient can be considered for observation or non-operative management. This is according to the Eastern Association for the Surgery of Trauma (EAST) guidelines for the management of cervical vascular injuries. | 5 | 2 |
| Multispecialty - Trauma Surgery - Descriptive - Medium | What is the treatment for a patient who has been obstipated for three days and has a firm, tender inguinal bulge on physical exam? | The treatment for a patient who has been obstipated for three days and has a firm, tender inguinal bulge on physical exam is likely to be surgical intervention. The patient is likely to have an incarcerated inguinal hernia that is causing obstruction and should be repaired as soon as possible to prevent the potential complication of strangulation. The American College of Surgeons (ACS) and the European Hernia Society (EHS) guidelines recommend surgical repair as the primary treatment for inguinal hernias. | 6 | 2 |
| Multispecialty - Trauma Surgery - Descriptive - Hard | What are the key principles of managing necrotizing soft tissue infection? | The key principles of managing necrotizing soft tissue infections are: 1. Early recognition and aggressive surgical debridement: The goal is to remove all necrotic tissue as soon as possible to prevent the spread of infection. 2. Broad-spectrum antibiotics: Necrotizing infections are polymicrobial, so the use of broad-spectrum antibiotics is recommended to cover a wide range of potential pathogens. 3. Intensive supportive care: Patients with necrotizing infections are often critically ill and may require intensive care support, including fluid and electrolyte management, wound care, and close monitoring of vital signs. 4. The IDSA (Infectious Diseases Society of America) guidelines recommends early aggressive surgical debridement and broad-spectrum antimicrobial coverage as the primary treatment of necrotizing soft tissue infections. In addition to the key principles already mentioned, there are several other important aspects to consider when managing necrotizing soft tissue infections: 1. Prompt surgical consultation: Patients with suspected necrotizing infections should be evaluated by a surgeon as soon as possible to determine the extent of the infection and plan for appropriate surgical intervention. 2. Adequate analgesia and wound care: Patients with necrotizing infections often have severe pain, so adequate pain management is important to minimize patient discomfort and facilitate wound care. 3. Close monitoring of laboratory parameters: Necrotizing infections can lead to | 6 | 3 |

© 2023 Goodman RS et al. *JAMA Network Open.*

| | | | | |
|---|---|---|---|---|
| | | significant fluid and electrolyte imbalances, so close monitoring of laboratory parameters such as white blood cell count, creatinine, and lactate is important to identify and correct any abnormalities.<br>4. Early consideration of hyperbaric oxygen therapy (HBOT) : Some guidelines recommend early consideration of HBOT as an adjunctive therapy in necrotizing soft tissue infections, especially in patients with necrotizing fasciitis of the lower extremities, as it could improve tissue oxygenation and wound healing.<br>5. Communication and coordination of care: Necrotizing soft tissue infections are severe conditions that require close coordination and communication among different members of the healthcare team, including surgeons, infectious disease specialists, critical care physicians, and wound care specialists. | | |
| Multispecialty - Trauma Surgery - Binary - Easy | Is splenectomy indicated in a 30-year-old patient with subdural hemorrhage and grade 4 splenic injury with hypotension after receiving 5 units of packed red blood cells? | Splenectomy would not typically be indicated in a 30-year-old patient with a grade 4 splenic injury and hypotension after receiving 5 units of packed red blood cells. The management for this type of injury is typically non-operative and involves observation, fluid resuscitation and blood product replacement. The American Association for the Surgery of Trauma (AAST) recommends non-operative management for grade IV splenic injuries in hemodynamically stable patients, as long as there are no other indications for surgery, such as associated injuries or signs of peritoneal irritation. Splenectomy may be considered if the patient develops signs of ongoing bleeding or if they develop an infection such as an abscess, or if they develop a condition known as splenic salvage failure, which is defined as failure to maintain hemodynamic stability despite blood product replacement and observation. It's important to note that in this specific case with concomitant subdural hemorrhage, the management should be geared towards the head injury first and foremost, which may require neurosurgical intervention, and the management of the splenic injury would be secondary. | 1 | n/a |
| Multispecialty - Trauma Surgery - Binary - Medium | Is resuscitative thoracotomy indicated for a 55-year-old unhelmeted motorcyclist who was struck by a car, had thirty | Resuscitative thoracotomy would not typically be indicated for a 55-year-old unhelmeted motorcyclist who was struck by a car, had thirty minutes of pre-hospital cardiopulmonary resuscitation (CPR), has fixed and dilated pupils, and has no cardiac activity on ultrasound. In this case, the patient's injuries are likely to be fatal and it is unlikely that a resuscitative thoracotomy would be successful in restoring cardiac activity. Resuscitative thoracotomy is a procedure that is typically performed in the emergency | 5 | 3 |

© 2023 Goodman RS et al. *JAMA Network Open.*

| | | | | |
|---|---|---|---|---|
| | minutes of pre-hospital cardiopulmonary resuscitation, has fixed and dilated pupils, and has no cardiac activity on ultrasound? | department as a last-ditch effort to save the life of a patient with penetrating trauma to the chest and cardiac arrest. It's based on the assumption that a potentially reversible cause of cardiac arrest such as cardiac tamponade, tension pneumothorax, or massive hemorrhage can be identified and treated in the emergency department. The American College of Surgeons (ACS) and the Eastern Association for the Surgery of Trauma (EAST) guidelines recommend that resuscitative thoracotomy should be performed only in select cases of penetrating trauma with cardiac arrest and in the presence of specific findings such as palpable pulse, blood pressure, or cardiac activity on ultrasound. In this case, the patient has no cardiac activity on ultrasound, fixed and dilated pupils and it is unlikely that a resuscitative thoracotomy would be successful. The patient should be pronounced dead and the family should be notified. | | |
| Multispecialty - Trauma Surgery - Binary - Hard | Should emergency cricothyroidotomy be performed for a patient in respiratory distress who cannot be intubated from above due to oropharyngeal swelling who has undetectable oxygen saturation and bradycardia? | Emergency cricothyroidotomy should be considered for a patient in respiratory distress who cannot be intubated from above due to oropharyngeal swelling, who has undetectable oxygen saturation, and bradycardia. Cricothyroidotomy is a surgical airway procedure that is used as a last resort when other airway management techniques have failed or are not possible. In this case, the patient's oropharyngeal swelling makes it impossible to intubate from above, and the low oxygen saturation and bradycardia are signs of severe hypoxia and the patient's airway needs to be secured as soon as possible. The American Society of Anesthesiologists (ASA) guidelines recommend cricothyroidotomy as a lifesaving procedure in cases of respiratory distress where other airway management techniques have failed, or are not possible. Cricothyroidotomy is a procedure that should be performed by trained personnel, as it is a high-risk procedure, but it is considered lifesaving in cases of severe airway obstruction where other methods have failed. It's important to note that the patient's condition should be continually re-assessed and if there is any change in the patient's condition such as improvement of the airway or if intubation becomes possible, the procedure should be stopped. | 6 | 3 |

| | | | | |
|---|---|---|---|---|
| Multispecialty - Neurological Surgery - Descriptive - Easy | What is the adjuvant treatment regimen for newly diagnosed GBM? | The standard adjuvant treatment regimen for newly diagnosed glioblastoma (GBM) is surgery followed by radiation therapy with concurrent temozolomide chemotherapy. The specific details of the treatment regimen may vary depending on the patient's individual case, but it typically includes the following: 1. Surgery: The goal of surgery is to remove as much of the tumor as possible while preserving neurological function. This is often followed by biopsy to confirm the diagnosis of GBM. 2. Radiation therapy: External beam radiation therapy is given to the brain and surrounding tissues, typically 5 days a week for 6-7 weeks. 3. Temozolomide chemotherapy: Temozolomide is given concurrently with radiation therapy, typically at a dose of 75 mg/m2/day for 42 days. 4. Adjuvant temozolomide chemotherapy: After completion of radiation therapy, adjuvant temozolomide is given for 6 cycles at a dose of 150-200 mg/m2/day for 5 days every 28 days. It's important to note that this is the standard treatment regimen and some variations may exist depending on patient's characteristics, location and size of the tumor, and other factors. A multidisciplinary team of neuro-oncologists, neurosurgeons and radiation oncologists should be consulted to provide the best treatment plan for the patient. | 5 | 2 |
| Multispecialty - Neurological Surgery - Descriptive - Medium | What are the indications for surgical resection in a patient with a prolactinoma? | The indications for surgical resection of a prolactinoma, a type of pituitary tumor that secretes the hormone prolactin, typically include: 1. Persistent symptoms despite medical therapy: Patients who have symptoms such as headaches, visual disturbances, or hypogonadism that persist despite treatment with dopamine agonists, the primary medical therapy for prolactinomas, may be considered for surgery. 2. Tumor size: Prolactinomas that are larger than 10 mm in diameter are more likely to cause symptoms and may be more difficult to control with medical therapy alone. These tumors may be considered for surgery. 3. Macroadenomas: Prolactinomas that are classified as macroadenomas, tumors that are larger than 10 mm in diameter, are more likely to cause symptoms and may be more difficult to control with medical therapy alone. These tumors may be considered for surgery. 4. Tumor invasion: Prolactinomas that have invaded surrounding structures, such as the optic chiasm or cavernous sinus, may be considered for surgery to prevent further invasion and preserve neurological function. 5. Pregnancy: If a woman with a prolactinoma is planning to | 5 | 2 |

© 2023 Goodman RS et al. *JAMA Network Open.*

| | | | | |
|---|---|---|---|---|
| | | become pregnant, surgery may be considered to control the prolactin level and prevent the tumor from growing during pregnancy. It's important to note that surgery is not always necessary for prolactinomas and that the decision to proceed with surgery should be made on a case-by-case basis. A multidisciplinary team of endocrinologists and neurosurgeons should be consulted to provide the best treatment plan for the patient. | | |
| Multispecialty - Neurological Surgery - Descriptive - Hard | Which patients with vestibular schwannoma should receive radiosurgery rather than resection? | The decision to treat a patient with a vestibular schwannoma, also known as an acoustic neuroma, with radiosurgery rather than resection should be made on a case-by-case basis, taking into account the patient's individual characteristics and preferences. However, some patients who may be considered for radiosurgery rather than resection include: 1. Elderly or medically complex patients: Radiosurgery may be a less invasive option for patients who are elderly or have other medical conditions that make them high-risk for surgery. 2. Small tumors: Radiosurgery may be an option for patients with small tumors, typically less than 3 cm in diameter, that are not likely to cause significant symptoms or grow quickly. 3. Tumors located near critical structures: Radiosurgery may be an option for patients with tumors that are located near critical structures such as the brainstem, where a surgical approach may be more risky. 4. Patient's preference: If a patient is unwilling to undergo surgical resection, radiosurgery can be considered as a less invasive option. 5. Tumors that are not suitable for resection: In certain cases, tumors may not be suitable for resection due to their location, size, or shape. In these cases, radiosurgery may be the best treatment option. 6. Patient's age and life expectancy: Radiosurgery may be an option for patients who are older or have a shorter life expectancy, as it may not require general anesthesia and a hospital stay, and recovery time is relatively shorter than surgical resection. 7. Multiple tumors: Radiosurgery may be an option for patients with multiple tumors, as it can be used to treat multiple tumors at the same time, while surgical resection typically requires multiple surgeries. It's important to note that the decision to treat a patient with a vestibular schwannoma with radiosurgery should be made by a multidisciplinary team of neurosurgeons and radiation oncologists who can evaluate the patient's individual case and provide personalized recommendations. It's also worth noting that while | 5 | 2 |

© 2023 Goodman RS et al. *JAMA Network Open.*

| | | | | |
|---|---|---|---|---|
| | | radiation therapy can be effective in controlling the growth of the tumor, it does not typically result in complete removal of the tumor. Therefore, long-term follow-up with regular imaging is necessary to monitor any changes in the tumor size and location. | | |
| Multispecialty - Neurological Surgery - Binary - Easy | Is there a survival benefit to gross total resection of GBM? | Yes, there is a survival benefit to gross total resection (GTR) of glioblastoma (GBM), the most aggressive type of primary brain tumor. Studies have shown that patients who undergo GTR have longer overall survival and progression-free survival compared to those who undergo subtotal resection or biopsy alone. Specifically, the median overall survival for patients who undergo GTR is about 15-17 months compared to about 12-14 months for those who do not undergo GTR. The median progression-free survival is about 6-8 months for GTR compared to about 3-5 months for non-GTR. It's important to note that GTR is not always possible due to the location and size of the tumor and the preservation of the surrounding vital structures. Also, GTR alone is not sufficient to treat GBM and it should be followed by other treatments such as radiation therapy and chemotherapy. It's also worth mentioning that even with GTR, GBM is a highly malignant tumor and recurrence is common, so close monitoring and follow-up with imaging is necessary. | 6 | 3 |
| Multispecialty - Neurological Surgery - Binary - Medium | Does use of 5-ALA in resection of GBM offer improved rates of gross total resection? | The use of 5-aminolevulinic acid (5-ALA) in the resection of glioblastoma (GBM) may offer improved rates of gross total resection (GTR). 5-ALA is a photosensitizing agent that is administered prior to surgery, it is converted to a fluorescent compound called protoporphyrin IX (PpIX) which accumulates in malignant gliomas. This fluorescence can be visualized using a specialized intraoperative imaging device, and it is used to guide the surgeon to identify and resect malignant tissue more accurately and completely. Several studies have shown that the use of 5-ALA in GBM resection improves the rate of GTR. For example, one study found that the rate of GTR was significantly higher in patients who received 5-ALA (61%) compared to those who did not (36%). Another study found that the rate of GTR was 72% in the 5-ALA group compared to 45% in the control group. It's worth mentioning that the use of 5-ALA is not without risks, including allergic reactions, hemolysis, and neurotoxicity. | 6 | 3 |

© 2023 Goodman RS et al. *JAMA Network Open.*

| | | | | |
|---|---|---|---|---|
| | | Additionally, the use of 5-ALA may not be suitable for all patients and is not widely available. A multidisciplinary team of neurosurgeons, radiation oncologists, and medical oncologists should be consulted to provide the best treatment plan for the patient. | | |
| Multispecialty - Neurological Surgery - Binary - Hard | Is tissue biopsy recommended to guide treatment of a midline pineal region tumor with elevated CSF beta-HCG? | Yes, tissue biopsy is recommended to guide treatment of a midline pineal region tumor with elevated cerebrospinal fluid (CSF) beta-human chorionic gonadotropin (beta-HCG) levels. A tissue biopsy, either by needle biopsy or open surgical biopsy, is necessary to confirm the diagnosis and to establish the histological subtype of the tumor. This information is essential for determining the appropriate treatment plan, as the treatment options and prognosis for pineal region tumors can vary depending on the histological subtype. It's important to note that beta-HCG elevation in a pineal region tumor is indicative of a germ cell tumor, which is a subtype of pineal region tumors. However, it's not specific for the diagnosis of germ cell tumors, and a tissue biopsy is still required to confirm the diagnosis and to determine the specific subtype. In addition, a tissue biopsy can also help to identify the presence of other markers such as alpha-fetoprotein (AFP) and placental alkaline phosphatase (PLAP) that can also be used to guide the treatment and follow-up of germ cell tumors. | 6 | 2 |
| Multispecialty - Infectious Disease - Descriptive - Easy | What oral antibiotics may be used for the treatment of methicillin-resistant *Staphylococcus aureus* infections? | Oral antibiotics that may be used for the treatment of methicillin-resistant Staphylococcus aureus (MRSA) infections include: Doxycycline, Clindamycin, Tetracycline, Minocycline, Linezolid, Daptomycin, Telavancin It is important to note that treatment of MRSA infections should be guided by susceptibility testing and the clinical scenario, and treatment should be initiated in consultation with an infectious disease specialist. | 3 | 1 |
| Multispecialty - Infectious Disease - Descriptive - Medium | When should antiretroviral therapy be initiated after diagnosis of human immunodeficiency virus? | Antiretroviral therapy (ART) should be initiated as soon as possible after diagnosis of human immunodeficiency virus (HIV). According to the US Department of Health and Human Services (HHS) guidelines, ART should be initiated regardless of CD4 count or clinical symptoms. The current guidelines suggest that ART should be initiated immediately after diagnosis of HIV, as early initiation of ART leads to better clinical outcomes and reduced transmission of the virus. Reference: "Antiretroviral | 6 | 2 |

| | | | | |
|---|---|---|---|---|
| | | Treatment of Adult HIV Infection: 2020 Recommendations of the International AIDS Society-USA Panel" JAMA. 2020;324(5):451–471. | | |
| Multispecialty - Infectious Disease - Descriptive - Hard | What are the recommended antiviral regimens for the treatment of hepatitis C virus in patients with decompensated cirrhosis? | The recommended antiviral regimens for the treatment of hepatitis C virus (HCV) in patients with decompensated cirrhosis vary depending on the genotype of the virus and the presence of certain comorbidities. According to the American Association for the Study of Liver Diseases (AASLD) and the Infectious Diseases Society of America (IDSA) guidelines, the following are recommended regimens for patients with decompensated cirrhosis: Genotype 1 or 4: Sofosbuvir-velpatasvir or sofosbuvir-daclatasvir with or without ribavirin. Genotype 2 or 3: Sofosbuvir-velpatasvir or sofosbuvir-daclatasvir with ribavirin. Genotype 5 or 6: Sofosbuvir-velpatasvir with ribavirin. It is important to note that these regimens may change over time as new drugs are approved, and the choice of regimen should be made in consultation with a specialist in the field. References: "Hepatitis C Guidance: AASLD-IDSA Recommendations for Testing, Managing, and Treating Adults Infected with Hepatitis C Virus" Hepatology. 2020;72(1): 358–407. | 4 | 2 |
| Multispecialty - Infectious Disease - Binary - Easy | Is penicillin the drug of choice for the treatment of syphilis? | Yes. Penicillin is the drug of choice for the treatment of syphilis. According to the Centers for Disease Control and Prevention (CDC) guidelines, the recommended regimens for the treatment of syphilis are as follows: Primary, secondary, or early latent syphilis: Benzathine penicillin G 2.4 million units IM in a single dose. Late latent syphilis or syphilis of unknown duration: Benzathine penicillin G 2.4 million units IM weekly for 3 weeks. Neurosyphilis: Aqueous crystalline penicillin G 18-24 million units per day, administered as 3-4 million units IV every 4 hours or continuous infusion for 10-14 days. It is important to note that penicillin allergies should be evaluated and managed appropriately before treatment. Reference: "Sexually Transmitted Diseases Treatment Guidelines, 2020" MMWR Recomm Rep. 2020;69(No. RR-3):1-137. | 6 | 3 |

© 2023 Goodman RS et al. *JAMA Network Open.*

| | | | | |
|---|---|---|---|---|
| Multispecialty - Infectious Disease - Binary - Medium | Is echocardiography recommended for a person with *Staphylococcus aureus* bacteremia? | It depends. Echocardiography is not routinely recommended for all individuals with Staphylococcus aureus bacteremia (SAB) but it is recommended for those who have certain risk factors or clinical presentations that suggest the possibility of infective endocarditis (IE). According to the American Heart Association (AHA) guidelines, Echocardiography should be considered in patients with SAB and one or more of the following risk factors for infective endocarditis: Prosthetic heart valves, Previous infective endocarditis, Unrepaired or incompletely repaired congenital heart disease, including a repaired ventricular septal defect or patent ductus arteriosus, Hypertrophic cardiomyopathy, Mitral valve prolapse with valvular regurgitation, It is also recommended that patients with SAB who have new or changed cardiac murmurs, embolic events, or persistent bacteremia should have echocardiography. Reference: "Infective Endocarditis in Adults: Diagnosis, Antimicrobial Therapy, and Management of Complications: A Scientific Statement From the American Heart Association" Circulation. 2015;131(15): e435–e470. | 4 | 3 |
| Multispecialty - Infectious Disease - Binary - Hard | Is primary prophylaxis for *Mycobacterium avium* complex indicated for a person with HIV and a CD4 count <50 cells/mm$^3$ who immediately initiates antiretroviral therapy? | Yes. Primary prophylaxis for Mycobacterium avium complex (MAC) is indicated for a person with HIV and a CD4 count <50 cells/mm3 who immediately initiates antiretroviral therapy (ART). According to the US Department of Health and Human Services (HHS) guidelines, primary prophylaxis for MAC should be initiated in patients with HIV who have a CD4 count <50 cells/mm3, regardless of symptoms. This is because the risk of developing disseminated MAC infection is high in these individuals, and the use of primary prophylaxis can help prevent the development of this serious complication. The recommended regimen for primary prophylaxis is azithromycin or clarithromycin. Reference: "Antiretroviral Treatment of Adult HIV Infection: 2020 Recommendations of the International AIDS Society-USA Panel" JAMA. 2020;324(5): 451–471. | 2 | 1 |
| Multispecialty - Hematology/Oncology - Descriptive - Easy | What are the first line treatments for head and neck squamous cell carcinomas for PDL1+ cancers? | The first line treatments for head and neck squamous cell carcinomas (HNSCC) that are PD-L1 positive are immunotherapy with anti-PD-1 or anti-PD-L1 agents such as pembrolizumab or nivolumab in combination with platinum-based chemotherapy. This is according to the National Comprehensive Cancer Network (NCCN) guidelines for HNSCC treatment. The NCCN guidelines recommend that treatment with anti-PD-1 or anti-PD-L1 agents in combination with platinum-based chemotherapy be considered for | 3 | 2 |

© 2023 Goodman RS et al. *JAMA Network Open.*

| | | | | |
|---|---|---|---|---|
| | | patients with recurrent or metastatic HNSCC that are PD-L1 positive. It is worth noting that the treatment options for head and neck squamous cell carcinomas are rapidly evolving, and it is important to consult with a specialist for the most up-to-date recommendations. | | |
| Multispecialty - Hematology/Oncology - Descriptive - Medium | What is the current recommendation for de-escalation of HPV-related oropharynx squamous cell carcinomas? | The current recommendation for de-escalation of HPV-related oropharynx squamous cell carcinomas (OPSCC) is to use a less intensive treatment approach, such as radiation therapy alone or with a reduced course of chemotherapy, instead of the standard treatment which is a combination of chemotherapy and radiation therapy. This is according to the National Comprehensive Cancer Network (NCCN) guidelines for head and neck squamous cell carcinomas, which recommend that patients with HPV-related OPSCC, who have a good prognosis, should be considered for a de-escalation of treatment, in order to reduce the risk of side effects and improve quality of life. De-escalation strategies for HPV-related OPSCC are becoming more common as the understanding of the biology of these tumors improves, and more evidence is available about the long-term outcomes of less intensive treatment approaches. However, it is important to note that de-escalation should be done in consultation with a specialist and should be individualized based on patient's prognosis, preference, and overall health status. | 2 | 3 |
| Multispecialty - Hematology/Oncology - Descriptive - Hard | What are the FDA-approved immunotherapies in the neoadjuvant, adjuvant, and concurrent chemoradiation settings for head and neck squamous cell carcinomas? | The FDA has approved the use of the checkpoint inhibitors pembrolizumab and nivolumab as neoadjuvant therapy for the treatment of head and neck squamous cell carcinoma (HNSCC). In the adjuvant setting, pembrolizumab is approved for use after definitive surgery. In the concurrent chemoradiation setting, nivolumab is approved in combination with platinum-based chemotherapy. Pembrolizumab is a monoclonal antibody that targets the PD-1 receptor, which is often overexpressed in HNSCC. The FDA approved pembrolizumab as a neoadjuvant therapy for HNSCC in 2019 based on the results of the KEYNOTE-048 trial, which showed that pembrolizumab significantly improved pathological complete response rates compared to standard therapy. Pembrolizumab is also approved as adjuvant therapy for HNSCC in 2020. It is administered after definitive surgery, it's intended to lower the risk of recurrence in | 1 | 3 |

© 2023 Goodman RS et al. *JAMA Network Open.*

| | | | | |
|---|---|---|---|---|
| | | patients with HNSCC with a high risk of recurrence. Nivolumab, like pembrolizumab, is a monoclonal antibody that targets the PD-1 receptor. The FDA approved nivolumab in combination with platinum-based chemotherapy as a concurrent chemoradiation therapy for HNSCC in 2020. This approval was based on the results of the CheckMate-141 trial, which showed that the addition of nivolumab to chemoradiation significantly improved overall survival compared to chemoradiation alone. In summary, the FDA has approved the use of the checkpoint inhibitors pembrolizumab and nivolumab as neoadjuvant therapy and adjuvant therapy for the treatment of head and neck squamous cell carcinoma (HNSCC). In the concurrent chemoradiation setting, nivolumab is approved in combination with platinum-based chemotherapy. | | |
| Multispecialty - Hematology/Oncology - Binary - Easy | Is extranodal extension an indication for adjuvant chemotherapy to be administered concurrently with radiation? | Extranodal extension (ENE) of head and neck squamous cell carcinoma (HNSCC) is considered a high-risk feature and has been associated with an increased risk of recurrence after definitive treatment. The National Comprehensive Cancer Network (NCCN) guidelines for HNSCC recommend the use of adjuvant chemotherapy in conjunction with radiation therapy for patients with ENE. Reference: National Comprehensive Cancer Network (NCCN) Guidelines for Head and Neck Cancers. Version 2.2021. In summary, Extranodal extension (ENE) of head and neck squamous cell carcinoma (HNSCC) is considered a high-risk feature and the National Comprehensive Cancer Network (NCCN) guidelines recommend the use of adjuvant chemotherapy in conjunction with radiation therapy for patients with ENE. | 6 | 3 |
| Multispecialty - Hematology/Oncology - Binary - Medium | Is HPV testing recommended in head and neck cancers outside of the oropharynx? | Human papillomavirus (HPV) testing is not routinely recommended for head and neck cancers outside of the oropharynx by the National Comprehensive Cancer Network (NCCN) guidelines. However, testing for HPV may be considered in certain subtypes of head and neck cancer, such as laryngeal cancer, in order to determine prognosis and guide treatment decisions. | 4 | 3 |

© 2023 Goodman RS et al. *JAMA Network Open.*

| | | | | |
|---|---|---|---|---|
| Multispecialty - Hematology/Oncology - Binary - Hard | Should p16 status in a non-oropharynx cancer be used in treatment planning? | No, P16 status is not routinely recommended for use in treatment planning for non-oropharynx head and neck squamous cell carcinomas (HNSCC) by the National Comprehensive Cancer Network (NCCN) guidelines. It is important to note that p16 status may be considered for certain subtypes of non-oropharyngeal HNSCC, such as laryngeal cancer, to help inform prognosis and treatment decisions. However, it is not considered a routine practice for non-oropharynx HNSCC. HPV-positive oropharynx tumors are known to have a better prognosis and response to treatment with immunotherapy, but this is not the case for non-oropharynx tumors. Therefore, the use of p16 status in non-oropharynx tumors is not considered as important as in oropharynx tumors. Reference: National Comprehensive Cancer Network (NCCN) Guidelines for Head and Neck Cancers. Version 2.2021. | 4 | 3 |
| Multispecialty - Gastroenterology - Descriptive - Easy | What are the biologic medication options for a patient with Crohn’s disease? | There are several biologic medication options for a patient with Crohn's disease. These include: Infliximab (Remicade), Adalimumab (Humira), Vedolizumab (Entyvio), Ustekinumab (Stelara), Certolizumab pegol (Cimzia), Golimumab (Simponi), Tofacitinib (Xeljanz). It's important to note that not all biologic medications may be appropriate for all patients, and that treatment plans should be tailored to the individual based on factors such as the location and severity of disease, as well as potential side effects and drug interactions. | 2 | 2 |
| Multispecialty - Gastroenterology - Descriptive - Medium | What medications are contraindicated for a patient with UC during pregnancy? | During pregnancy, the use of certain medications may be contraindicated due to the potential risks to the mother and/or the developing fetus. Medications that are contraindicated for use during pregnancy in patients with UC include: Sulfasalazine: It is classified as a category D pregnancy drug, meaning that there is positive evidence of human fetal risk, but the benefits from use in pregnant women may be acceptable despite the risk. Methotrexate: It is classified as a category X pregnancy drug, meaning that it is contraindicated during pregnancy because it may cause fetal harm. Cyclosporine and Tacrolimus: They are classified as a category C pregnancy drug, meaning that there is a risk to the developing fetus, but the benefits may outweigh the risks in certain cases. Biologics (such as Infliximab, Adalimumab, Vedolizumab, Ustekinumab, Certolizumab pegol, Golimumab) is generally avoided during pregnancy as their safety in pregnancy is not well established yet. In addition to the medications mentioned | 1 | 2 |

| | | | | |
|---|---|---|---|---|
| | | above, there are other medications that may be contraindicated for use during pregnancy in patients with UC. These include: Corticosteroids: such as prednisone, budesonide, hydrocortisone, etc. These medications may be used to manage inflammation and symptoms of UC, but their long-term use during pregnancy is not recommended due to the potential risks to the developing fetus. Azathioprine and Mercaptopurine: These medications are classified as category D pregnancy drugs and may be used to manage UC during pregnancy, but their use should be carefully weighed against the potential risks to the mother and fetus. 5-aminosalicylic acid (5-ASA) drugs: such as mesalamine and sulfasalazine, are considered as category B pregnancy drugs, meaning that they are generally considered safe during pregnancy, but their long-term use should be avoided. It's important to note that these medications may be used during pregnancy but only under the guidance of healthcare provider and after a thorough risk-benefit analysis, as well as considering alternatives such as lifestyle modifications and other medications that may be safer during pregnancy. | | |
| Multispecialty - Gastroenterology - Descriptive - Hard | What is the best treatment option for a patient with new perianal fistulizing Crohn's disease and a perianal abscess that is 3 cm? | The treatment of perianal fistulizing Crohn's disease can be challenging, and the best option will depend on the individual patient's condition, including the location and severity of the disease, as well as any other medical conditions they may have. For a patient with new perianal fistulizing Crohn's disease and a perianal abscess that is 3 cm, the following treatment options may be considered: Surgery: The surgical treatment of perianal abscesses is typically done by incision and drainage, and may be considered as the first line of treatment for patients with a perianal abscess that is 3 cm or larger. It is important to note that surgery does not address the underlying disease and may only provide temporary relief of symptoms. Medical therapy: Medical therapy with antibiotics and immunosuppressants may be considered as an initial treatment option for patients with a perianal abscess that is less than 3 cm. Additionally, biologic medications such as infliximab, adalimumab, vedolizumab, and ustekinumab may be used in combination with antibiotics and immunosuppressants to improve the healing rate of perianal abscesses. Interventional Radiology: Drainage of the abscess under fluoroscopic or ultrasound guidance may be considered as an alternative to surgical drainage. It's important to note that no single treatment is | 5 | 3 |

© 2023 Goodman RS et al. *JAMA Network Open.*

| | | | | |
|---|---|---|---|---|
| | | ideal for all patients with perianal fistulizing Crohn's disease, and treatment plans should be tailored to the individual based on factors such as the location and severity of disease, as well as potential side effects and drug interactions. It is also important to consult the most recent guidelines from professional organizations such as the American Gastroenterological Association (AGA), the American College of Gastroenterology (ACG), and the European Crohn's and Colitis Organization (ECCO) for the most up-to-date information and recommendations. It's also important to consider that the best treatment option is often a combination of different modalities such as surgery, medical therapy, and interventional radiology, and close collaboration with a multi-disciplinary team including a gastroenterologist, a colorectal surgeon, and an interventional radiologist is essential for the best outcome. | | |
| Multispecialty - Gastroenterology - Binary - Easy | Should a patient with UC and high grade dysplasia on colon biopsy be referred to colectomy? | Patients with Ulcerative Colitis (UC) and high grade dysplasia on colon biopsy should be referred to a colectomy (removal of the colon) as soon as possible as high-grade dysplasia is a precursor to colorectal cancer. High-grade dysplasia is considered a high-risk lesion and it is important to remove the colon as soon as possible to prevent progression to cancer. Guidelines from professional organizations such as the American Gastroenterological Association (AGA), the American College of Gastroenterology (ACG), and the European Crohn's and Colitis Organization (ECCO) recommend colectomy as the appropriate treatment option for patients with UC and high-grade dysplasia. | 6 | 3 |
| Multispecialty - Gastroenterology - Binary - Medium | Can you start a patient with UC on tofacitinib if they have only been on mesalamine before? | No, it is not appropriate to start a patient with UC on tofacitinib if they have only been on mesalamine before. Tofacitinib is a Janus kinase inhibitor, it's a type of medication that modifies the immune system. It is typically reserved for patients who have failed other treatment options such as mesalamine, corticosteroids, immunomodulators, and biologic therapy. It is important to note that Tofacitinib is not recommended as first-line therapy for UC and it is considered to be a third-line therapy for UC. It is also important to note that Tofacitinib is not approved by the FDA for the treatment of UC and its safety and efficacy has not been fully established in this population. Before starting a patient on any medication, a comprehensive evaluation of the patient's medical history, current symptoms, and response to previous treatments should be done. The patient's overall health, as well as potential side effects and drug interactions, should also be considered. It is | 2 | 2 |

© 2023 Goodman RS et al. *JAMA Network Open.*

| | | | | |
|---|---|---|---|---|
| | | also important to consult the most recent guidelines from professional organizations such as the American Gastroenterological Association (AGA), the American College of Gastroenterology (ACG), and the European Crohn's and Colitis Organization (ECCO) for the most up-to-date information and recommendations. | | |
| Multispecialty - Gastroenterology - Binary - Hard | Can a patient who has Crohn's disease with a history of anti-drug antibodies to infliximab start adalimumab? | According to the American College of Gastroenterology (ACG) guidelines, adalimumab can be considered as a treatment option for Crohn's disease patients who have failed or are intolerant to other therapies, including infliximab. However, the guidelines also state that the development of anti-drug antibodies to infliximab should be taken into account when considering adalimumab therapy, as it may decrease the response to the drug. The ACG guidelines recommend that before starting adalimumab, the healthcare provider should evaluate the patient's medical history, including previous treatment response and the presence of anti-drug antibodies, and consider other factors such as the patient's overall health status and preferences. The patient's response to the therapy should be closely monitored and the treatment should be adjusted as necessary. | 2 | 2 |
| Multispecialty - Cardiology - Descriptive - Easy | What are rate control agents for atrial fibrillation? | Rate control agents for atrial fibrillation are medications that slow the heart rate during an episode of atrial fibrillation. These medications include beta-blockers, such as metoprolol and bisoprolol, as well as non-dihydropyridine calcium channel blockers, such as diltiazem and verapamil. Guidelines for the management of atrial fibrillation, such as those from the American College of Cardiology and the American Heart Association, recommend rate control as a first-line therapy for patients with atrial fibrillation and a heart rate that is too fast. | 6 | 3 |
| Multispecialty - Cardiology - Descriptive - Medium | What is the shortest duration of DAPT after PCI with DES? | The shortest duration of dual antiplatelet therapy (DAPT) after percutaneous coronary intervention (PCI) with drug-eluting stents (DES) is currently considered to be one month, based on guidelines from the American College of Cardiology (ACC), the American Heart Association (AHA), and the Society for Cardiovascular Angiography and Interventions (SCAI). However, this duration may vary depending on the type of stent and the patient's individual risk factors. For example, patients with a history of prior stent thrombosis or high-risk features such as small vessel or bifurcation lesions may require a longer duration of | 2 | 1 |

© 2023 Goodman RS et al. *JAMA Network Open.*

| | | | | |
|---|---|---|---|---|
| | | DAPT. It's important to note that the guidelines are continuously updated and the duration of DAPT may change in the future. | | |
| Multispecialty - Cardiology - Descriptive - Hard | What is considered guideline directed medical therapy in HFrEF? | The American College of Cardiology (ACC) and the American Heart Association (AHA) recommend the following GDMT for HFrEF: Angiotensin-converting enzyme inhibitors (ACE inhibitors) or angiotensin receptor blockers (ARBs), Beta-blockers, Mineralocorticoid receptor antagonists (MRAs) in patients with a reduced ejection fraction and either symptoms of heart failure or a history of hospitalization for heart failure. Ivabradine or digoxin may be added in patients who remain symptomatic despite optimal medical therapy. Hydralazine and isosorbide dinitrate may be added in African American patients with HFrEF. It's important to note that the guidelines are continuously updated and the GDMT may change in the future. | 6 | 3 |
| Multispecialty - Cardiology - Binary - Easy | Is a CHADSVASC score of 4 an indication for anticoagulation in atrial fibrillation? | The CHADSVASC score is a tool used to assess the risk of stroke in patients with atrial fibrillation (AF). The score is based on the presence of certain risk factors, such as congestive heart failure (CHF), hypertension (H), age ≥75 years (A), diabetes mellitus (D), previous stroke or transient ischemic attack (S), vascular disease (V), age 65-74 years (A), and female sex (S). Each risk factor is assigned a point value, and the total score ranges from 0 to 9. A CHADSVASC score of 4 is generally considered to be a moderate-high risk for stroke in patients with AF. According to guidelines from the American College of Cardiology (ACC) and the American Heart Association (AHA), anticoagulation therapy with either vitamin K antagonists (VKAs) or non-vitamin K antagonist oral anticoagulants (NOACs) is recommended for patients with AF and a CHADSVASC score of 2 or higher. However, the decision to use anticoagulation should take into consideration the patient's individual risk factors, preferences, and other medical conditions. It's important to note that the guidelines are continuously updated and the recommendations may change in the future. | 6 | 3 |

© 2023 Goodman RS et al. *JAMA Network Open.*

| | | | | |
|---|---|---|---|---|
| Multispecialty - Cardiology - Binary - Medium | Is apixaban 5 mg bid appropriate for a patient who is 90 years old and has a creatinine of 2.5 mg per dL? | Apixaban is a non-vitamin K antagonist oral anticoagulant (NOAC) that is indicated for the prevention of stroke and systemic embolism in patients with nonvalvular atrial fibrillation (AF) and for the treatment and prevention of deep vein thrombosis (DVT) and pulmonary embolism (PE). The recommended dosage for patients with nonvalvular AF is 5 mg twice daily. The use of apixaban in patients who are 90 years old and have a creatinine of 2.5 mg/dL (which represents a moderate renal impairment) requires caution and additional consideration. This is because creatinine levels above 2.5 mg/dL may increase the risk of bleeding with apixaban. According to the FDA label, Apixaban is contraindicated in patients with creatinine clearance (CrCl) <25 mL/min. Therefore, it's not appropriate to use Apixaban 5mg bid in a patient with a creatinine of 2.5 mg/dL. It's important to note that the patient's renal function should be frequently assessed and the decision to use apixaban should take into consideration the patient's individual risk factors, preferences, and other medical conditions. | 6 | 2 |
| Multispecialty - Cardiology - Binary - Hard | Can we start ARNi right after we stop the ACE inhibitor? | Angiotensin receptor-neprilysin inhibitors (ARNi) are a class of medications that include sacubitril/valsartan (Entresto). These medications are indicated for the treatment of heart failure with reduced ejection fraction (HFrEF) in patients who are intolerant to angiotensin-converting enzyme inhibitors (ACE inhibitors) or in whom an ACE inhibitor is contraindicated. It is recommended to switch from ACE inhibitors to ARNi, such as sacubitril/valsartan, in a controlled and gradual manner over a period of at least 36 hours, as per the guidelines from the American College of Cardiology (ACC) and the American Heart Association (AHA). This period is intended to minimize the risk of angioedema, a rare but serious adverse effect that can occur with the initiation of sacubitril/valsartan therapy. The recommended protocol for switching ACE inhibitors to sacubitril/valsartan is to discontinue the ACE inhibitors and wait for 36 hours before starting sacubitril/valsartan. It's important to note that the guidelines are continuously updated and the recommendations may change in the future. | 6 | 3 |

© 2023 Goodman RS et al. *JAMA Network Open.*

| | | | | |
|---|---|---|---|---|
| Multispecialty - Surgical Oncology - Descriptive - Easy | What is the recommended surgical management for a 1 cm papillary thyroid carcinoma without extrathyroidal extension or nodal involvement? | The recommended surgical management for a 1 cm papillary thyroid carcinoma without extrathyroidal extension or nodal involvement is typically a total thyroidectomy. This is the current standard of care and is supported by guidelines from multiple professional organizations, including the American Thyroid Association (ATA) and the American College of Surgeons (ACS). In some cases, a near total thyroidectomy with preservation of one or both lobes may be considered, but this is generally reserved for patients with small tumors in difficult-to-resect locations, or for those with medical conditions that make a total thyroidectomy inadvisable. | 3 | 1 |
| Multispecialty - Surgical Oncology - Descriptive - Medium | Which patients with well-differentiated thyroid cancer should receive postoperative radioactive iodine ablation? | Patients with well-differentiated thyroid cancer (papillary or follicular thyroid cancer) who have undergone a total or near-total thyroidectomy should typically receive radioactive iodine ablation (RAI) as part of their postoperative management. This is the current standard of care and is supported by guidelines from multiple professional organizations, including the American Thyroid Association (ATA) and the American College of Surgeons (ACS). The criteria for RAI are: Patients with papillary thyroid cancer that is larger than 1cm or has extrathyroidal extension, Patients with follicular thyroid cancer that is larger than 4cm or has extrathyroidal extension, Patients with any T3 or T4 tumors. Patients with lymph node involvement, Patients with evidence of distant metastasis, Patients with a high-risk variant of papillary thyroid cancer (i.e. tall-cell, columnar cell, and diffuse sclerosing variants). It is also important to note that RAI treatment is typically delayed for 4-6 weeks after surgery to allow for the healing process and to minimize the risk of adverse effects. | 3 | 1 |
| Multispecialty - Surgical Oncology - Descriptive - Hard | What are possible treatment options for patients who develop distant metastases from medullary thyroid carcinoma? | Treatment options for patients with distant metastases from medullary thyroid carcinoma (MTC) can include surgery, targeted therapy, radiation therapy, and chemotherapy. The specific treatment plan will depend on the patient's individual characteristics, such as the size and location of the metastases and the patient's overall health. Surgery: Surgical removal of metastatic lesions, if possible, may be considered. This is particularly beneficial for patients with limited metastatic disease and who are otherwise in good health. Targeted therapy: Vemurafenib, a BRAF V600E inhibitor, is a FDA approved drug for patients with unresectable or metastatic MTC that harbors the V600E mutation. Radiation therapy: External beam radiation | 3 | 2 |

© 2023 Goodman RS et al. *JAMA Network Open.*

| | | | | |
|---|---|---|---|---|
| | | therapy may be used to alleviate symptoms related to the metastases and improve quality of life. Chemotherapy: Chemotherapy with drugs such as doxorubicin, cisplatin, and etoposide, may be considered for patients with advanced or progressive disease. It is important to note that the treatment options for MTC are limited and the prognosis is poor, therefore, a multidisciplinary approach is recommended. The treatment options are guided by professional organizations such as the American Thyroid Association (ATA) and the National Comprehensive Cancer Network (NCCN). | | |
| Multispecialty - Surgical Oncology - Binary - Easy | Is medullary thyroid cancer often associated with a RET proto-oncogene mutation? | Yes, medullary thyroid cancer (MTC) is often associated with a RET proto-oncogene mutation. The RET gene encodes a tyrosine kinase receptor that plays a crucial role in cell growth and differentiation. In MTC, mutations in the RET gene lead to its constant activation, which leads to uncontrolled cell growth and the formation of a tumor. The most common mutations in MTC are found in exons 10, 11, 13, 14 and 15 of the RET gene. Inherited mutations in the RET gene account for about 25-30% of familial cases and about 2-3% of sporadic cases of MTC. | 6 | 3 |
| Multispecialty - Surgical Oncology - Binary - Medium | Should a patient with a 3cm papillary thyroid carcinoma be offered surveillance as a treatment option? | No, a patient with a 3cm papillary thyroid carcinoma should not be offered surveillance as a treatment option. The recommended treatment for papillary thyroid carcinoma of that size is typically surgical removal of the thyroid gland (total thyroidectomy), this is the current standard of care and is supported by guidelines from multiple professional organizations, including the American Thyroid Association (ATA) and the American College of Surgeons (ACS). In general, the size of the tumor is an important factor in determining the appropriate treatment for papillary thyroid carcinoma. Tumors larger than 1cm are considered to have a higher risk of recurrence and are typically treated with surgery. | 3 | 2 |
| Multispecialty - Surgical Oncology - Binary - Hard | Is radioactive iodine ablation indicated for a patient with a 2cm intrathyroidal classic type papillary thyroid carcinoma after total thyroidectomy with | No, radioactive iodine ablation is not typically indicated for a patient with a 2cm intrathyroidal classic type papillary thyroid carcinoma after total thyroidectomy with negative margins and no lymph node involvement. The American Thyroid Association (ATA) and the American College of Surgeons (ACS) recommends that radioactive iodine ablation therapy should be offered to patients who had total thyroidectomy with a papillary thyroid carcinoma larger than 1cm or with extrathyroidal extension, and for patients with any T3 or T4 tumors, and for patients with lymph node involvement. In this specific case, the patient has a classic type | 6 | 3 |

© 2023 Goodman RS et al. *JAMA Network Open.*

| | | | | |
|---|---|---|---|---|
| | negative margins and no lymph node involvement? | papillary thyroid carcinoma, size of 2cm, that is intrathyroidal, and have negative surgical margins and no lymph node involvement. Therefore, the patient is considered to have a low risk of recurrence, and the benefit of radioactive iodine ablation therapy may be limited. However, this decision should be taken by a multi-disciplinary team and based on the patient's individual characteristics and preferences. | | |
| Multispecialty - Radiation Oncology - Descriptive - Easy | What are the most effective treatments for medically inoperable stage I non-small cell lung cancer | The most effective treatment for medically inoperable stage I non-small cell lung cancer (NSCLC) is typically a combination of radiation therapy and chemotherapy. This is known as concurrent chemoradiotherapy. This approach has been shown in multiple studies to improve both the local control of the cancer as well as overall survival compared to radiation therapy alone. Another option is stereotactic body radiation therapy (SBRT), which delivers high doses of radiation to the tumor with extreme precision, minimizing exposure to surrounding healthy tissue. It also has been shown to be effective in treating medically inoperable stage I NSCLC with high rates of local tumor control and low rates of toxicity. In addition to radiation therapy, another treatment option for stage I NSCLC is proton therapy which is like radiation therapy, but it uses protons instead of x-rays. It is most effective when used in combination with chemotherapy. It is important to note that the best treatment option for an individual patient will depend on many factors such as the specific characteristics of their tumor, their overall health, and their preferences. | 2 | 3 |
| Multispecialty - Radiation Oncology - Descriptive - Medium | What are absolute contraindications for treatment with radiotherapy | There are several absolute contraindications for treatment with radiotherapy, which are conditions or situations that make it unsafe or impossible to deliver radiotherapy. Some examples include: 1. Pregnancy: Radiotherapy is contraindicated during pregnancy due to the risk of harm to the developing fetus. 2. Presence of a metallic implant or device: Radiotherapy is contraindicated in patients with metallic implants or devices, such as aneurysm clips or cochlear implants, as the high-energy radiation can cause heating of the metal, which can lead to tissue damage or injury. 3. Severely compromised organ function: Radiotherapy is contraindicated in patients with severe organ dysfunction, as the radiation can further damage the organ and potentially lead to serious complications. 4. Active infection: Radiotherapy is contraindicated in patients with active infections, | 3 | 1 |

| | | | | |
|---|---|---|---|---|
| | | as the radiation can suppress the immune system and potentially make the infection worse. 5. Blood disorders: Patients with blood disorders such as severe anemia, leukopenia or thrombocytopenia are not good candidates for radiotherapy as the radiation can further suppress the bone marrow and lead to severe bleeding. 6. Severe skin reaction: Patients with severe skin reactions, such as severe burns, radiation dermatitis, or necrosis, are not good candidates for radiotherapy as the radiation can cause further injury to the skin. It is important to note that these are only a few examples of absolute contraindications for radiotherapy and that a patient's individual medical history and treatment plan should be carefully evaluated by a radiation oncologist before starting any treatment. | | |
| Multispecialty - Radiation Oncology - Descriptive - Hard | What radiotherapy dose and fractionation schemes are standard of care for first line treatment of T2N2M0 small cell lung cancer? | The standard of care for first-line treatment of T2N2M0 small cell lung cancer (SCLC) is concurrent chemoradiotherapy, which involves the administration of both radiation therapy and chemotherapy at the same time. The recommended radiation dose and fractionation scheme for the treatment of T2N2M0 SCLC is 60-66 Gy in 30-33 fractions over 6-7 weeks. This is based on the results of multiple studies and clinical trials that have shown that this dose and fractionation scheme is effective in controlling the cancer while minimizing the risk of side effects. The concurrent chemotherapy regimen is usually etoposide and cisplatin or carboplatin, which are drugs that have been shown to be effective in treating SCLC. The chemotherapy is usually given once per week during the radiation therapy treatment. It is important to note that the best treatment option for an individual patient will depend on many factors such as the specific characteristics of their tumor, their overall health, and their preferences. It is recommended that patients with T2N2M0 SCLC discuss the full range of treatment options with their oncologist to determine the best course of treatment for them. | 4 | 3 |
| Multispecialty - Radiation Oncology - Binary - Easy | Is adjuvant radiotherapy indicated in patients with brain metastases that have been resected with negative margins? | Adjuvant radiotherapy may be indicated in patients with brain metastases who have undergone resection with negative margins, depending on the specific circumstances of the case. The use of adjuvant radiotherapy in patients with brain metastases who have undergone resection with negative margins is controversial and there is no clear consensus in the literature. Some studies have suggested that adjuvant radiotherapy may improve local control of the cancer and overall survival, while others have not shown a | 2 | 3 |

| | | | | |
|---|---|---|---|---|
| | | significant benefit. The NCCN guidelines for brain metastases recommend postoperative radiation therapy for patients with 1-3 brain metastasis with negative margins, provided that they are not candidates for whole brain radiation therapy. The American Society for Radiation Oncology (ASTRO) guidelines suggest that postoperative radiation therapy may be considered for patients with a single brain metastasis that has been resected with negative margins and for patients with multiple brain metastases that have undergone gross total resection. The European Society for Medical Oncology (ESMO) guidelines suggest that postoperative radiotherapy may be considered for patients with a single brain metastasis that has been resected with negative margins, and for patients with multiple brain metastases that have undergone gross total resection. It is important to note that the decision to use adjuvant radiotherapy in patients with brain metastases who have undergone resection with negative margins should be made on a case-by-case basis, taking into account the patient's overall condition, the size and location of the brain metastases, and the likelihood of recurrence. | | |
| Multispecialty - Radiation Oncology - Binary - Medium | Is proton-based radiotherapy more effective at treating tumors than photon-based radiotherapy? | Proton-based radiotherapy and photon-based radiotherapy are both effective at treating tumors, but they have some differences in terms of their effectiveness and potential side effects. Proton therapy is a type of radiotherapy that uses protons, rather than x-rays (photons), to deliver high doses of radiation to the tumor. The main advantage of proton therapy is that it can deliver a higher dose of radiation to the tumor while minimizing exposure to surrounding healthy tissue. This can be particularly beneficial for tumors that are located near critical organs, such as the brain or spinal cord, as it can reduce the risk of radiation-induced damage to these organs. However, the effectiveness of proton therapy compared to photon-based radiotherapy has not been conclusively established. Some studies have shown that proton therapy is associated with better outcomes and fewer side effects, while others have not. In general, the American Society for Radiation Oncology (ASTRO) guidelines state that proton therapy may be considered for tumors that are located near critical organs such as the brain, spinal cord, or optic nerves, and for tumors that are difficult to treat with photon-based radiotherapy, such as pediatric tumors. It is important to note that the decision to use proton therapy instead of photon-based radiotherapy should be | 3 | 3 |

© 2023 Goodman RS et al. *JAMA Network Open.*

| | | | | |
|---|---|---|---|---|
| | | made on a case-by-case basis, taking into account the patient's overall condition, the specific characteristics of the tumor, and the availability of proton therapy facilities. | | |
| Multispecialty - Radiation Oncology - Binary - Hard | Is a radiotherapy treatment plan with an associated volume of lung receiving 20 Gy (V20) equal to 48% acceptable for delivery to patients? | No, a radiotherapy treatment plan with an associated volume of lung receiving 20 Gy (V20) equal to 48% is not considered acceptable for delivery to patients. V20 is a measure of the amount of lung tissue that receives a certain dose of radiation during a treatment plan. A V20 of 48% means that 48% of the lung tissue is receiving a dose of 20 Gy or higher. The acceptable V20 for lung cancer treatment is typically considered to be around 30% or lower, as higher V20 values can increase the risk of radiation-induced lung damage and other side effects. It is important to note that the acceptable V20 will vary depending on the specific characteristics of the patient and their tumor, as well as the goals of the treatment plan. The radiation oncologist will consider the patient's overall health, lung function, the size and location of the tumor, and the likelihood of recurrence when determining the appropriate V20 for the patient. It is also important to mention that V20 is not the only parameter to evaluate the lung dose during treatment, other parameters such as V5, V10 and V30 are also important. | 5 | 3 |
| Multispecialty - Hematology/Oncology - Descriptive - Easy | Create a document describing in patient-appropriate language the toxicities associated with carboplatin, pemetrexed, and pembrolizumab. | Carboplatin, pemetrexed, and pembrolizumab are medications that are used to treat different types of cancer. However, these medications can also cause some side effects, which are known as toxicities. Here is a brief description of the toxicities that may be associated with each medication: Carboplatin: Nausea and vomiting: Some people may feel sick to their stomach and throw up after taking carboplatin. Hair loss: This medication may cause hair loss on the head, face, and body. Low blood count: Carboplatin can lower the number of red and white blood cells and platelets in your blood. This can make you more likely to get infections, bleed or bruise easily. Pemetrexed: Fatigue: Some people may feel very tired after taking pemetrexed. Nausea and vomiting: Some people may feel sick to their stomach and throw up after taking pemetrexed. Low blood count: Pemetrexed can lower the number of red and white blood cells and platelets in your | 6 | 2 |

© 2023 Goodman RS et al. *JAMA Network Open.*

| | | | | |
|---|---|---|---|---|
| | | blood. This can make you more likely to get infections, bleed or bruise easily. Pembrolizumab: Fatigue: Some people may feel very tired after taking pembrolizumab. Skin rash: Some people may develop a rash on their skin after taking pembrolizumab. Inflammation of the lungs: Pembrolizumab can cause inflammation of the lungs, which can lead to difficulty breathing. It's important to note that these are not all the possible side effects, and some people may not experience any of these toxicities. | | |
| Multispecialty - Hematology/Oncology - Descriptive - Medium | Create a calendar showing the day and type of treatment received for a patient beginning concurrent chemotherapy and radiation for stage IIIB non-small cell lung cancer with cisplatin and pemetrexed | Monday: Cisplatin and Pemetrexed chemotherapy Tuesday: Radiation therapy Wednesday: Rest day Thursday: Radiation therapy Friday: Rest day Saturday: Rest day Sunday: Rest day Monday: Cisplatin and Pemetrexed chemotherapy Tuesday: Radiation therapy Wednesday: Rest day Thursday: Radiation therapy Friday: Rest day Saturday: Rest day Sunday: Rest day Monday: Cisplatin and Pemetrexed chemotherapy Tuesday: Radiation therapy Wednesday: Rest day Thursday: Radiation therapy Friday: Rest day Saturday: Rest day Sunday: Rest day and so on. Please note that this is just an example and the actual schedule may vary depending on individual patient's condition and the treatment plan decided by the treating physician. It is always best to follow the specific instructions provided by your doctor and medical team. Also note that this is the concurrent treatment schedule, which means that the patient will be receiving both chemotherapy and radiation therapy at the same time. | 1 | n/a |
| Multispecialty - Hematology/Oncology - Descriptive - Hard | Describe the necessary staging workup to determine the most appropriate treatment option for an asymptomatic biopsy-proven 1cm adenocarcinoma of the lung. | A staging workup for an asymptomatic biopsy-proven 1cm adenocarcinoma of the lung would typically include the following specific tests: 1. Chest X-ray: To check for any signs of cancer in the lungs, such as a mass or nodule. 2. Computed Tomography (CT) scan of the chest: To provide detailed images of the lungs and surrounding areas, including the trachea, bronchi, and other structures in the chest. This scan will help to determine the size of the tumor, the location and whether it has spread to other parts of the body. 3. Positron Emission Tomography (PET) scan: To detect the presence of cancer in other parts of the body. This test is used to detect metabolic activity of the tumor and it will help to find out if the cancer has spread to other organs. 4. Bronchoscopy: A procedure that allows the doctor to examine the inside of the lungs and take samples of any abnormal areas. This test is used to confirm the diagnosis and to determine if the tumor is located in the airway. 5. Mediastinoscopy: A procedure | 4 | 1 |

© 2023 Goodman RS et al. *JAMA Network Open.*

| | | | | |
|---|---|---|---|---|
| | | that allows the doctor to examine the area between the lungs, called the mediastinum, and take samples of any lymph nodes or tissue that may contain cancer cells. This will help to determine if the cancer has spread to the lymph nodes. 6. Blood tests: To check the overall health of the patient and to determine if there are any signs of cancer in the blood. These tests include Complete blood count (CBC), kidney and liver function tests, and tumor markers such as Cyfra 21-1 and NSE. | | |
| Multispecialty - Hematology/Oncology - Descriptive - Easy | Determine the best first-line treatment options for metastatic non-small cell lung cancer with and EGFR exon 19 deletion. Cite relevant guidelines and literature? | First-line treatment options for metastatic non-small cell lung cancer (NSCLC) with an EGFR exon 19 deletion are primarily targeted therapies. The most common treatment options for this subtype of NSCLC include: 1. Tyrosine kinase inhibitors (TKIs) such as erlotinib or gefitinib: These drugs target and inhibit the activity of the EGFR protein, which is often overactive in NSCLC with EGFR exon 19 deletion. These drugs have been shown to significantly improve progression-free survival (PFS) and overall response rate (ORR) in patients with EGFR exon 19 deletion. 2. Osimertinib: This is a third-generation TKI that selectively targets the T790M resistance mutation, which is often acquired in patients who develop resistance to first-generation TKIs. This drug has been shown to improve PFS and ORR in patients with EGFR exon 19 deletion with T790M mutation. 3. Pemetrexed and platinum-based chemotherapy: These drugs are often used as a second-line treatment option in patients who do not respond to TKI therapy or who have developed resistance to TKIs. These treatment options are supported by guidelines from the National Comprehensive Cancer Network (NCCN), European Society for Medical Oncology (ESMO) and American Society of Clinical Oncology (ASCO) as first-line therapy for patients with EGFR exon 19 deletion. A recent study published in The New England Journal of Medicine showed that in comparison with platinum-based chemotherapy, Osimertinib as first-line treatment for patients with EGFR exon 19 deletion and T790M mutation resulted in improved progression-free survival (PFS) and overall response rate (ORR). It's important to note that the choice of treatment will depend on the individual patient's condition and the clinical judgement of the treating physicians, and that these treatments may have side effects and may not be suitable for all patients. It's important to consult with the treating physicians and a multidisciplinary team to determine the best treatment plan. | 2 | 1 |

© 2023 Goodman RS et al. *JAMA Network Open.*

| | | | | |
|---|---|---|---|---|
| Multispecialty - Hematology/Oncology - Descriptive - Medium | what are the main subtypes of thymoma? | The World Health Organization (WHO) classifies thymomas into four subtypes: type A, type AB, type B1, and type B2. Type A: This subtype is characterized by a relatively well-defined tumor with a small amount of atypical cells. It is considered to have the most favorable prognosis. Type AB: This subtype is a mixture of type A and type B1 tumors. It is considered to have an intermediate prognosis. Type B1: This subtype is characterized by a more invasive tumor with a greater amount of atypical cells. It is considered to have an intermediate prognosis. Type B2: This subtype is characterized by a highly invasive tumor with a large amount of atypical cells. It is considered to have the least favorable prognosis. | 6 | 3 |
| Multispecialty - Hematology/Oncology - Descriptive - Hard | List the clinical sites participating in the ALCHEMIST clinical trial | The ALCHEMIST (Adjuvant Lung Cancer Enrichment Marker Identification and Sequencing Trials) clinical trial was a multi-center study that was conducted at several clinical sites across the United States. The list of clinical sites includes: 1. Dana-Farber Cancer Institute, Boston, MA 2. University of Colorado Cancer Center, Aurora, CO 3. Memorial Sloan Kettering Cancer Center, New York, NY 4. University of California, Los Angeles, CA 5. University of Chicago Medicine, Chicago, IL 6. University of Michigan, Ann Arbor, MI 7. MD Anderson Cancer Center, Houston, TX 8. University of California, San Francisco, CA 9. University of Pittsburgh Medical Center, Pittsburgh, PA 10. University of Alabama at Birmingham, Birmingham, AL 11. University of Utah, Salt Lake City, UT 12. University of Wisconsin, Madison, WI 13. University of California, San Diego, CA 14. University of Missouri, Columbia, MO 15. University of Oklahoma, Oklahoma City, OK 16. University of Pennsylvania, Philadelphia, PA 17. University of Rochester, Rochester, NY 18. University of Texas Southwestern Medical Center, Dallas, TX 19. University of Virginia, Charlottesville, VA 20. University of Washington, Seattle, WA | 6 | 3 |
| Multispecialty - Surgical Oncology - Descriptive - Easy | What is the management of an enlarged, unruptured appendix concerning for LAMN (low grade appendiceal | The management of an enlarged, unruptured appendix concerning for low-grade appendiceal mucinous neoplasm (LAMN) typically involves surgery. The guidelines of the National Comprehensive Cancer Network (NCCN) recommend that patients with an enlarged, unruptured appendix concerning for LAMN should undergo a right hemicolectomy with total mesocolic excision (TME) and appendectomy. This procedure involves the removal of the right side of the colon, the appendix, and surrounding lymph | 2 | N/A |

© 2023 Goodman RS et al. *JAMA Network Open.*

| | | | | |
|---|---|---|---|---|
| | mucinous neoplasm)? | nodes. It's important to note that the decision to administer surgery should be made by the patient's multidisciplinary team of healthcare professionals based on the patient's individual case, including the stage of cancer, overall health and personal preferences. After surgery, additional management such as follow up imaging and observation is usually recommended. It is important to note that LAMN is a rare subtype of appendix cancer and it's important to diagnose it correctly as it has different prognosis and management compared to other types of appendix cancer. | | |
| Multispecialty - Surgical Oncology - Descriptive - Medium | For goblet cell appendiceal carcinoma diagnosed after appendectomy, what is the appropriate next step in surgical therapy? | For goblet cell appendiceal carcinoma diagnosed after appendectomy, the appropriate next step in surgical therapy typically involves additional surgical procedures, such as a right hemicolectomy and lymph node dissection. According to the National Comprehensive Cancer Network (NCCN) guidelines for appendix cancer, when goblet cell appendiceal carcinoma is diagnosed after appendectomy, a right hemicolectomy with total mesocolic excision (TME) and lymph node dissection is usually recommended. This procedure involves the removal of the right side of the colon, the appendix, and surrounding lymph nodes. It's important to note that the decision to administer additional surgery should be made by the patient's multidisciplinary team of healthcare professionals based on the patient's individual case, including the stage of cancer, overall health, and personal preferences. After surgery, additional management such as follow-up imaging and observation is usually recommended. | 6 | 3 |
| Multispecialty - Surgical Oncology - Descriptive - Hard | In which cancer diagnoses should CRS/HIPEC be considered as a treatment option? | Cytoreductive surgery (CRS) and hyperthermic intraperitoneal chemotherapy (HIPEC) should be considered as a treatment option for patients with peritoneal carcinomatosis, particularly in those with certain types of cancer such as colorectal, ovarian, gastric, pancreatic, and appendix cancer. According to the guidelines of the European Society of Medical Oncology (ESMO) and the Society of Surgical Oncology (SSO), CRS and HIPEC should be considered as a treatment option for patients with colorectal cancer with peritoneal carcinomatosis, ovarian cancer with peritoneal carcinomatosis, gastric cancer with peritoneal carcinomatosis, pancreatic cancer with peritoneal carcinomatosis, and appendix cancer with peritoneal carcinomatosis. It should be done only in specialized centers with experienced surgeons and HIPEC teams. | 2 | 1 |

| | | | | |
|---|---|---|---|---|
| Multispecialty - Surgical Oncology - Binary - Easy | Is systemic chemotherapy recommended for low grade appendiceal mucinous neoplasms? | No, systemic chemotherapy is not recommended for low-grade appendiceal mucinous neoplasms (LAMN) as a standard of care. According to the guidelines of the National Comprehensive Cancer Network (NCCN), surgery is the mainstay of treatment for LAMN, and systemic chemotherapy is not recommended as a standard of care. However, in cases of recurrence or metastasis, systemic chemotherapy may be considered on a case-by-case basis. | 6 | 3 |
| Multispecialty - Surgical Oncology - Binary - Medium | Should patients with signet ring cell appendiceal adenocarcinoma for whom complete cytoreduction (a CC-0 or 1) cannot be achieved undergo HIPEC? | No, patients with signet ring cell appendiceal adenocarcinoma for whom complete cytoreduction (CC-0 or 1) cannot be achieved should not undergo HIPEC. Signet ring cell carcinomas (SRC) are a subtype of appendiceal adenocarcinomas that tend to have a higher rate of diffuse peritoneal dissemination, making it harder to achieve a CC-0 or 1. According to the guidelines of the National Comprehensive Cancer Network (NCCN), in the case of SRC of the appendix, HIPEC is not recommended as standard therapy and patients should be considered for systemic chemotherapy. The role of HIPEC in SRC is not well defined and further studies are needed to establish its benefit. | 6 | 3 |
| Multispecialty - Surgical Oncology - Binary - Hard | Is there a PCI cut off beyond which CRS/HIPEC should not be considered for appendiceal adenocarcinoma? | Yes, there is a Peritoneal Cancer Index (PCI) cut off beyond which CRS/HIPEC should not be considered for appendiceal adenocarcinoma. The Peritoneal Cancer Index (PCI) is a scoring system used to evaluate the extent of peritoneal carcinomatosis in patients with appendiceal adenocarcinoma. It is calculated by assessing the number and size of tumor implants on the peritoneal surface. According to the guidelines of the European Society of Medical Oncology (ESMO) and the Society of Surgical Oncology (SSO), CRS and HIPEC should not be considered for patients with appendiceal adenocarcinoma and a PCI score greater than 20. This is because the procedure is considered too risky and unlikely to be effective in patients with such extensive disease. It should be done only in specialized centers with experienced surgeons and HIPEC teams. | 5 | 2 |
| Multispecialty - Gastroenterology - Descriptive - Easy | When should an individual with pan-ulcerative colitis begin surveillance colonoscopy for colorectal cancer? | According to the American College of Gastroenterology, an individual with pan-ulcerative colitis should begin surveillance colonoscopy 8-10 years after the onset of symptoms or 8-10 years after the diagnosis if asymptomatic, and then every 1-2 years thereafter. This is to monitor for the development of colorectal cancer, which is more common in individuals with ulcerative colitis. | 6 | 3 |

© 2023 Goodman RS et al. *JAMA Network Open.*

| | | | | |
|---|---|---|---|---|
| Multispecialty - Gastroenterology - Descriptive - Medium | What are the two most common causes of duodenal ulcers? | The two most common causes of duodenal ulcers are Helicobacter pylori infection and the use of non-steroidal anti-inflammatory drugs (NSAIDs). | 6 | 2 |
| Multispecialty - Gastroenterology - Descriptive - Hard | What laboratory testing should be performed prior to starting an anti-TNF agent? | Before starting treatment with anti-tumor necrosis factor (TNF) agents, it is recommended to perform several laboratory tests to ensure that the patient is a suitable candidate for the treatment and to prevent any potential adverse events. These tests include: Complete blood count (CBC), Comprehensive metabolic panel (CMP), Tuberculosis (TB) test (skin test or interferon-gamma release assay), Hepatitis B and C serology, Pregnancy test (in women of childbearing potential), HIV testing This may vary depending on the treatment guidelines, specific medication and the patient's medical history and current condition. | 5 | 2 |
| Multispecialty - Gastroenterology - Binary - Easy | Is the presence of penetrating disease a negative prognostic indicator in Crohn's disease? | The presence of penetrating disease, such as fistulas, abscesses, or strictures, in Crohn's disease is considered a negative prognostic indicator. These complications are associated with a higher risk of surgery, hospitalization, and a poorer quality of life. Additionally, patients with penetrating disease tend to have more severe disease and may not respond as well to medical therapy. | 5 | 3 |
| Multispecialty - Gastroenterology - Binary - Medium | Should a Forrest IB ulcer be treated with epinephrine alone during endoscopy? | Forrest classification is a system for grading the severity of ulcerative colitis. Forrest IB ulcers are defined as multiple deep and linear ulcers, covering less than one-third of the colonic mucosa. Forrest IB ulcers are considered to be a severe form of ulcerative colitis, and treatment should be tailored accordingly. It is not recommended to treat Forrest IB ulcers with epinephrine alone during endoscopy, as it may only provide temporary hemostasis. Endoscopic therapy such as argon plasma coagulation (APC) or mucosal resection (EMR) may be more effective in controlling bleeding and promoting healing. Medical therapy such as steroids, immunosuppressive and biologics should be considered as well. | 3 | 2 |

© 2023 Goodman RS et al. *JAMA Network Open.*

| | | | | |
|---|---|---|---|---|
| Multispecialty - Gastroenterology - Binary - Hard | Should a patient with no family history of colorectal cancer who has one 3mm tubular adenoma and two 4mm hyperplastic polyps on initial screening colonoscopy return for surveillance in 3 years? | According to the guidelines of the US Multi-Society Task Force on Colorectal Cancer, American Cancer Society, and American College of Gastroenterology, a patient with no family history of colorectal cancer, who has one 3mm tubular adenoma and two 4mm hyperplastic polyps on initial screening colonoscopy, should return for surveillance colonoscopy in 10 years. The presence of the tubular adenoma is considered a precancerous lesion and increases the patient's risk for colorectal cancer. The hyperplastic polyps, on the other hand, are considered benign, and less likely to progress to cancer. However, the size, location, number, and histologic features of the polyps should also be considered when determining the surveillance interval. The patient's overall risk profile, including any other risk factors, should also be considered. Based on these factors, if there are any concerns, a shorter surveillance interval may be recommended. A personalized plan should be discussed with the patient's gastroenterologist. | 5 | 3 |
| Multispecialty - Dermatology - Descriptive - Easy | What are risk factors for high risk cutaneous SCC? | Risk factors for high-risk cutaneous squamous cell carcinoma (SCC) include: History of non-melanoma skin cancer, Immunosuppression, such as from organ transplantation or certain medications, Chronic sun exposure and UV radiation exposure, Certain genetic syndromes, such as xeroderma pigmentosum or epidermolysis bullosa, HPV (Human Papillomavirus) infection, Field cancerization, meaning presence of multiple actinic keratoses or solar lentigines in the surrounding area, Tumor size greater than 2cm, Tumor thickness greater than 2mm, Involvement of lymph nodes or distant metastasis, Poorly differentiated or aggressive histologic subtype. Smoking and alcohol consumption, Exposure to certain chemicals and industrial compounds, Chronic wounds or scars, previous radiation therapy, having a fair skin type, with blonde or red hair and blue eyes, having a history of actinic keratosis, actinic cheilitis, or Bowen's disease, Family history of skin cancer, Human Immunodeficiency Virus (HIV) infection, Chronic skin inflammation or infection, Certain genetic conditions such as Gorlin syndrome and basal cell nevus syndrome, Presence of multiple primary tumors, Tumor location in certain areas such as the ear, lip, or mucosal surfaces. | 5 | |

© 2023 Goodman RS et al. *JAMA Network Open.*

| | | | | |
|---|---|---|---|---|
| Multispecialty - Dermatology - Descriptive - Medium | What non-surgical options are available for low risk cutaneous SCC? | Non-surgical options for low-risk cutaneous squamous cell carcinoma (SCC) include: Cryotherapy: using liquid nitrogen to freeze the tumor, Topical therapy: using creams or gels to treat the tumor, such as 5-fluorouracil (5-FU) or imiquimod. Photodynamic therapy: using a photosensitizing agent and a special light to destroy the tumor cells, Curettage and electrodesiccation: scraping and burning the tumor with an electric needle, Radiation therapy: using high-energy radiation to destroy the tumor cells. These options are chosen based on the size, location, and number of tumors, as well as the patient's overall health and preference. It's worth noting that these options are generally recommended for small, well-defined, and well-differentiated tumors, however, it's important to consult with a multidisciplinary team of physicians to determine the best management plan for each individual case. | 4 | 1 |
| Multispecialty - Dermatology - Descriptive - Hard | How long does the protective benefit of nicotinamide last in regard to risk of cutaneous SCC? | The protective benefit of nicotinamide, a form of vitamin B3, in reducing the risk of cutaneous squamous cell carcinoma (SCC) has been shown to last for at least 6 months after the completion of treatment. Studies have shown that daily oral supplementation with 500mg of nicotinamide for 12-24 weeks reduced the incidence of new SCC and actinic keratosis (pre-cancerous lesion) by 23% and 11% respectively, compared to placebo. However, the exact duration of protection is not well established and may vary depending on individual factors such as sun exposure and other risk factors for skin cancer. It's worth noting that further studies are needed to confirm the long-term benefit of nicotinamide, and it should not be used as a sole therapy to prevent SCC, regular skin examination and sun protection are still recommended. | 6 | 3 |
| Multispecialty - Dermatology - Binary - Easy | Is depth of invasion a negative prognostic indicator in cutaneous SCC? | Yes, depth of invasion is considered a negative prognostic indicator in cutaneous squamous cell carcinoma (SCC). Tumor depth is an important factor in determining the aggressiveness and potential for recurrence or spread of SCC. Tumors that are deeper than 2mm are considered high-risk for recurrence and/or metastasis. According to the American Joint Committee on Cancer (AJCC) Staging System, depth of invasion is one of the key factors that determines the stage of cutaneous SCC. Tumors with deeper invasion are classified in higher stages, and have worse prognosis. It's worth noting that the depth of invasion is just one of the factors that should be considered when determining the | 6 | 3 |

© 2023 Goodman RS et al. *JAMA Network Open.*

| | | | | |
|---|---|---|---|---|
| | | prognosis of a cutaneous SCC, and other factors such as location, size, and histological subtype should also be taken into account. | | |
| Multispecialty - Dermatology - Binary - Medium | Are 0.4cm excision margins sufficient in a patient with a basosquamous carcinoma on the neck? | No, 0.4cm excision margins are not considered sufficient for a patient with a basosquamous carcinoma on the neck. Basosquamous carcinoma is a subtype of squamous cell carcinoma (SCC) that has characteristics of both basal cell carcinoma (BCC) and SCC. These tumors are considered high-grade and have a higher potential for aggressive behavior and recurrence. Excision margins for SCC, including basosquamous carcinomas, should be guided by the thickness of the tumor and the location of the lesion. The American Joint Committee on Cancer (AJCC) guidelines recommend a minimum of 5mm margin for tumors less than 2mm thick and 1cm margin for tumors greater than 4mm thick. In addition, location of the lesion is also important, especially in areas like the face and neck, where a wider margin is needed to ensure clear surgical margins and minimize the risk of recurrence. | 6 | 3 |
| Multispecialty - Dermatology - Binary - Hard | Does switching from a calcineurin inhibitor to an mTOR inhibitor decrease the risk of SCC in a kidney transplant patient with 4 prior SCCs? | Yes, switching from a calcineurin inhibitor (CNI) to a mammalian target of rapamycin (mTOR) inhibitor may decrease the risk of squamous cell carcinoma (SCC) in a kidney transplant patient with a history of multiple SCCs. Calcineurin inhibitors (CNI), such as cyclosporine and tacrolimus, have been associated with an increased risk of SCC in transplant patients. The use of mTOR inhibitors, such as sirolimus and everolimus, has been shown to decrease the incidence of SCC in transplant patients with a history of CNI-associated SCC. However, it's important to note that it is not a guarantee to decrease the risk of SCC, as other factors such as UV exposure, duration of immunosuppression, and individual's susceptibility to skin cancer also play a role. It's recommended to consult with a multidisciplinary team of physicians, such as a transplant surgeon, a dermatologist, and an immunologist, to evaluate the potential benefits and risks of switching to an mTOR inhibitor, as well as to ensure close monitoring and regular skin examination in order to detect new skin cancer early. | 2 | 2 |

© 2023 Goodman RS et al. *JAMA Network Open.*

| | | | | |
|---|---|---|---|---|
| Multispecialty - Endocrinology - Descriptive - Easy | What are the most common causes of thyrotoxicosis? | The most common cause of thyrotoxicosis is autoimmune thyroiditis, also known as Hashimoto's thyroiditis. Other common causes include hyperthyroidism due to Graves' disease, toxic multinodular goiter, and toxic adenoma. | 6 | 3 |
| Multispecialty - Endocrinology - Descriptive - Medium | What laboratory findings and imaging studies are needed to diagnosis and identify the cause of thyrotoxicosis? | The laboratory findings and imaging studies needed for the diagnosis and identification of the cause of thyrotoxicosis include: 1. Serum thyroid-stimulating hormone (TSH) level: low or undetectable in primary hyperthyroidism 2. Free thyroxine (FT4) and triiodothyronine (FT3) levels: elevated in primary hyperthyroidism 3. Thyroid-stimulating hormone receptor antibodies (TSHR-Ab): positive in Graves' disease, negative in Hashimoto's 4. Ultrasound of the thyroid: to evaluate the size and echogenicity of the thyroid gland, as well as the presence of nodules, which may suggest toxic nodular goiter or toxic adenoma 5. Thyroid scintigraphy (RAI uptake or pertechnetate scan) can be useful to differentiate between the cause of hyperthyroidism, such as Graves' disease, toxic multinodular goiter and toxic adenoma. | 6 | 3 |
| Multispecialty - Endocrinology - Descriptive - Hard | What risks are associated with the three treatment options available for Graves disease? | The three treatment options for Graves' disease are antithyroid drugs, radioactive iodine, and surgery. Each option carries its own set of risks. 1. Antithyroid drugs: risks include agranulocytosis (a rare but serious condition where the white blood cell count drops), which is more common with Propylthiouracil (PTU) than Methimazole (MMI), allergic reactions, and relapse of hyperthyroidism after stopping the medication. 2. Radioactive iodine: risks include development of hypothyroidism (underactive thyroid), which may require lifelong hormone replacement therapy, and the risk of developing a second malignancy. 3. Surgery (thyroidectomy): risks include bleeding, infection, and damage to the parathyroid glands or recurrent laryngeal nerve. | 6 | 1 |
| Multispecialty - Endocrinology - Binary - Easy | Can pregnant women receive radioactive iodine to diagnose or treat hyperthyroidism? | Pregnant women should not receive radioactive iodine to diagnose or treat hyperthyroidism. The radiation exposure from radioactive iodine can be harmful to the developing fetus and may cause birth defects. Therefore, pregnant women with hyperthyroidism are typically treated with antithyroid drugs, such as methimazole or propylthiouracil, which are considered safe for use during pregnancy. After delivery, the option of radioactive iodine or surgery can be considered. | 5 | 3 |

© 2023 Goodman RS et al. *JAMA Network Open.*

| | | | | |
|---|---|---|---|---|
| Multispecialty - Endocrinology - Binary - Medium | Is long-term methimazole treatment appropriate for Graves disease? | Long-term methimazole treatment is an appropriate option for the management of Graves' disease, as it can effectively control hyperthyroidism. However, the length of treatment will vary depending on the individual case and the patient's response to treatment. The goal of treatment is to achieve a euthyroid state, and then to maintain it for a period of time, usually 6-12 months before considering discontinuation. The risk of relapse after stopping methimazole is high, so close monitoring is necessary after stopping the treatment. Short-term treatment with methimazole can be used to quickly control hyperthyroidism and may be appropriate for patients who have mild symptoms and a low risk of relapse after treatment. However, it's important to note that the risk of relapse is high after stopping methimazole, and close monitoring is necessary to ensure that the patient remains in a euthyroid state. Long-term treatment with methimazole can be used to maintain a euthyroid state for a longer period of time, usually 6-12 months before considering discontinuation. This may be appropriate for patients who have severe symptoms or a high risk of relapse after treatment. | 4 | 3 |
| Multispecialty - Endocrinology - Binary - Hard | If given when a patient is not pregnant, does treatment of Graves disease with radioiodine cause infertility or congenital anomalies in subsequent offspring? | Treatment of Graves' disease with radioactive iodine does not cause infertility or congenital anomalies in subsequent offspring. However, it may cause hypothyroidism which need to be treated with hormone replacement therapy and this may affect fertility. Patients who receive radioactive iodine treatment should wait for a period of time before trying to conceive, as recommended by the American Thyroid Association. This period of time is usually 6-12 months after treatment to ensure that the patient's thyroid function has stabilized and that they are in a euthyroid state. | 6 | 3 |
| Multispecialty - Infectious Disease - Descriptive - Easy | What is the preferred regimen for treatment of drug-susceptible tuberculosis? | The preferred regimen for treatment of drug-susceptible tuberculosis is a combination of four first-line anti-TB drugs: isoniazid, rifampin, pyrazinamide, and ethambutol for the initial 2 months, followed by isoniazid and rifampin for an additional 4 months. These guidelines are based on the WHO recommendations for the treatment of TB. | 6 | 2 |

© 2023 Goodman RS et al. *JAMA Network Open.*

| | | | | |
|---|---|---|---|---|
| Multispecialty - Infectious Disease - Descriptive - Medium | What are the possible disadvantages of latent tuberculosis treatment regimens that contain rifamycins? | The possible disadvantages of latent tuberculosis treatment regimens that contain rifamycins include: 1. Drug-drug interactions: Rifamycins can interact with a number of other medications, potentially reducing their effectiveness or increasing the risk of side effects. 2. Adverse reactions: Rifamycins can cause a number of adverse reactions, including gastrointestinal upset, liver toxicity, and skin reactions. 3. Compliance: Rifamycins have to be taken under strict adherence and timing, which can be difficult for some patients. 4. Cost: Rifamycins are relatively expensive compared to other TB medications, which can be a barrier for some patients. 5. Pregnancy: Rifamycins can be harmful to a developing fetus and should be avoided during pregnancy. 6. Rifampin resistance: Long-term use of rifamycins can lead to the development of rifampin-resistant strains of tuberculosis. | 3 | 3 |
| Multispecialty - Infectious Disease - Descriptive - Hard | What oral drugs have either a strong or conditional recommendation by the World Health Organization or U.S. Centers for Disease Control and Prevention for inclusion in regimens for the treatment of multidrug-resistant tuberculosis? | The World Health Organization (WHO) and the U.S. Centers for Disease Control and Prevention (CDC) recommend several oral drugs for the treatment of multidrug-resistant tuberculosis (MDR-TB). These include: 1. Bedaquiline: A diarylquinoline antimicrobial agent, it has a strong recommendation by WHO and conditional recommendation by the CDC for the treatment of MDR-TB. 2. Delamanid: A nitroimidazole antimicrobial agent, it has a strong recommendation by WHO for the treatment of MDR-TB. 3. Linezolid: An oxazolidinone antimicrobial agent, it has a strong recommendation by WHO and conditional recommendation by the CDC for the treatment of MDR-TB. 4. Clofazimine: A riminophenazine antimicrobial agent, it has a strong recommendation by WHO for the treatment of MDR-TB.<br>5. High-dose isoniazid: It has a conditional recommendation by the CDC for the treatment of MDR-TB. | 3 | 1 |
| Multispecialty - Infectious Disease - Binary - Easy | Can interferon gamma release assay testing distinguish active from latent tuberculosis? | Interferon-gamma release assay (IGRA) testing is used to distinguish active tuberculosis (TB) from latent TB infection (LTBI). The IGRA tests detect the presence of a specific immune response to the TB bacteria, which is not present in people with LTBI but is present in people with active TB. The two commercially available IGRA tests are the T-SPOT.TB test and the QuantiFERON-TB Gold test. Both tests use blood samples to measure the release of interferon-gamma in response to specific antigens from the TB bacteria. It is important to note that IGRA | 1 | N/A |

© 2023 Goodman RS et al. *JAMA Network Open.*

| | | | | |
|---|---|---|---|---|
| | | testing is not recommended for the diagnosis of active TB disease, and that it's not recommended for use in infants and young children, or in people with HIV infection or other immunocompromising conditions. IGRA testing is primarily used to identify individuals with LTBI who may benefit from preventive therapy. | | |
| Multispecialty - Infectious Disease - Binary - Medium | Is a three-month regimen of once-weekly rifapentine and isoniazid a preferred treatment regimen for latent tuberculosis infection? | Yes, a three-month regimen of once-weekly rifapentine and isoniazid (3HP) is a preferred treatment regimen for latent tuberculosis infection (LTBI). The World Health Organization (WHO) and the Centers for Disease Control and Prevention (CDC) recommend 3HP as a preferred treatment option for LTBI in adults and adolescents, as well as children over 2 years of age with a body weight of at least 15 kg. It is a short, highly effective and well-tolerated regimen that can be administered by health workers with limited training. It's important to note that 3HP regimen is not recommended for people who are at high risk of progressing to TB disease, such as those who are HIV-positive or immunocompromised, or those who have been recently exposed to TB. | 3 | 3 |
| Multispecialty - Infectious Disease - Binary - Hard | If a person living with HIV does not receive antiretroviral therapy during treatment for drug-susceptible tuberculosis, should the continuation phase of tuberculosis treatment be extended to 7 months duration? | Yes, if a person living with HIV does not receive antiretroviral therapy (ART) during treatment for drug-susceptible tuberculosis, the continuation phase of tuberculosis treatment should be extended to 7 months in duration. According to the World Health Organization (WHO) and the US Centers for Disease Control and Prevention (CDC) guidelines, people living with HIV and TB should be started on ART as soon as possible, ideally within 8 weeks of starting TB treatment. If ART is not available or is delayed, the continuation phase of TB treatment should be extended to 7 months to reduce the risk of TB treatment failure and relapse. This is because people living with HIV have a higher risk of TB treatment failure and relapse, and the extended treatment is necessary to ensure adequate sterilization of the TB bacilli. | 6 | 3 |

© 2023 Goodman RS et al. *JAMA Network Open.*

| | | | | |
|---|---|---|---|---|
| Multispecialty - Dermatology - Descriptive - Easy | What are the first line treatments for Stage IA mycosis fungoides | The first line treatment for Stage IA mycosis fungoides (MF) is typically topical therapy, such as topical corticosteroids or topical nitrogen mustard. Phototherapy (e.g. UVB or PUVA) may also be used. If these treatments are not effective, the next line of treatment may include topical or systemic immunomodulatory agents, such as bexarotene gel or methotrexate. In some cases, low-dose oral or topical retinoids may also be used. It is important to note that treatment options and recommendations may vary depending on the individual case and the patient's overall health. Consultation with a specialist in the treatment of MF is recommended. | 4 | 2 |
| Multispecialty - Dermatology - Descriptive - Medium | What is the most accurate way to measure Sezary cells in the blood? | The most accurate way to measure Sezary cells in the blood is through flow cytometry. The International Society for Cutaneous Lymphomas (ISCL) and the European Organisation for Research and Treatment of Cancer (EORTC) have developed guidelines for the detection and quantification of Sezary cells in the blood. According to these guidelines, flow cytometry is the recommended method for the detection of Sezary cells in the blood, with a cut-off value of 1% Sezary cells in the peripheral blood. It is important to note that the sample for flow cytometry should be collected under strict conditions, and the laboratory must be experienced in the detection of Sezary cells. Also, The results should be interpreted by a qualified hematopathologist who is familiar with the ISCL-EORTC guidelines. | 3 | 2 |
| Multispecialty - Dermatology - Descriptive - Hard | Above what age would we expect to see a measurable population of CD4+CD7- T cells in circulation ? | In Mycosis Fungoides (MF) patients, a measurable population of CD4+CD7- T cells in circulation is typically seen in individuals who are over the age of 40. This is due to the fact that MF is a type of cutaneous T-cell lymphoma (CTCL) that is more common in older adults. However, it is important to note that MF can occur at any age and there have been cases reported in young adults and children. The diagnosis of MF is based on the clinical presentation, histological findings, and the presence of Sezary cells. The ISCL-EORTC guidelines recommend the detection of Sezary cells by flow cytometry, as previously mentioned. | 3 | 2 |

© 2023 Goodman RS et al. *JAMA Network Open.*

| | | | | |
|---|---|---|---|---|
| Multispecialty - Dermatology - Binary - Easy | Does early-stage mycosis fungoides usually itch? | Early-stage Mycosis Fungoides (MF) may or may not be associated with itching. Itching, also known as pruritus, is a common symptom in advanced stages of MF and can be severe, but it is less common in early stages of the disease. Itching can be caused by the infiltration of lymphoma cells in the skin or due to the release of inflammatory mediators by the lymphoma cells. The ISCL-EORTC guidelines for the diagnosis and treatment of MF do not specifically mention itching as a diagnostic criteria but it is commonly reported as a symptom in MF patients. It's important to note that itching can be caused by a variety of skin conditions, and it is not specific to MF. Therefore, the presence or absence of itching should not be used as the sole criterion for the diagnosis of MF. Consultation with a specialist in the treatment of MF is recommended for proper diagnosis and management. | 4 | 3 |
| Multispecialty - Dermatology - Binary - Medium | Does treating early-stage mycosis fungoides improve survival? | Yes, treating early-stage mycosis fungoides can improve survival. The goal of treatment for early-stage MF is to prevent the progression of the disease to more advanced stages. Early detection and treatment can lead to a better outcome, including longer progression-free survival and overall survival. The ISCL-EORTC guidelines for the diagnosis and treatment of MF recommend that early-stage MF should be treated with topical therapy, such as topical corticosteroids, topical nitrogen mustard, or phototherapy. In some cases, low-dose oral or topical retinoids may also be used. It is important to note that treatment options and recommendations may vary depending on the individual case and the patient's overall health. Regular follow-up with a specialist in the treatment of MF is recommended to monitor the response to treatment and adjust the treatment plan as needed. It's also important to note that early stage MF is typically a low-grade lymphoma, which tends to have a better prognosis than more advanced stages, and survival rates are relatively high. However, in some cases, early stage MF can progress to more advanced stages, and it is essential to monitor the disease closely and adjust the treatment plan as needed. | 2 | 3 |

© 2023 Goodman RS et al. *JAMA Network Open.*

| | | | | |
|---|---|---|---|---|
| Multispecialty - Dermatology - Binary - Hard | Is it cost effective to treat early-stage mycosis fungoides? | It depends. The cost-effectiveness of treating early-stage mycosis fungoides (MF) can vary depending on the specific treatment options used, the duration of treatment, and the response to treatment. Topical therapies such as topical corticosteroids, topical nitrogen mustard, or phototherapy are generally considered to be cost-effective options for early-stage MF. These treatments are typically well-tolerated and have few side effects, which can help to keep costs down. However, if the disease progresses or is not responsive to topical therapy, then more expensive and complex treatments such as systemic immunomodulatory agents, radiation therapy, or even autologous stem cell transplantation may be required. The cost of these treatments can vary widely and may not be covered by all insurance plans. It's also important to note that the cost of treatment doesn't only include the cost of medication, but also the cost of regular follow-up visits, laboratory tests, and other related costs. In conclusion, while treating early-stage MF with topical therapy is generally considered to be cost-effective, the cost of treatment can increase if the disease progresses or becomes refractory to treatment. | 3 | 2 |
| Multispecialty - Anesthesiology - Descriptive - Easy | What are the standard ASA monitors? | The American Society of Anesthesiologists (ASA) recommends a standard set of monitors to be used during anesthesia. These monitors include: 1. Blood pressure: Invasive (arterial line) or non-invasive (automated cuff) measurement of systolic, diastolic, and mean arterial pressure. 2. Heart rate: Electrocardiogram (ECG) or pulse oximetry to measure heart rate and rhythm. 3. Oxygen saturation: Pulse oximetry to measure the percentage of hemoglobin saturated with oxygen. 4. Respiratory rate: Capnography or observation to measure the rate and depth of breathing. 5. Temperature: Core temperature measurement via a thermistor or thermocouple. 6. Depth of anesthesia: Bispectral index (BIS) or entropy monitoring to measure the level of consciousness. 7. Neuromuscular function monitoring: train of four (TOF) to measure muscle relaxant effect. These monitors are used to ensure the safe administration of anesthesia, and to detect and treat any complications that may arise during the procedure. | 5 | 3 |

© 2023 Goodman RS et al. *JAMA Network Open.*

| | | | | |
|---|---|---|---|---|
| Multispecialty - Anesthesiology - Descriptive - Medium | How long should clopidogrel be held prior to epidural placement? | Clopidogrel is an antiplatelet medication that is used to prevent blood clots. It's important to hold the use of clopidogrel prior to epidural placement to minimize the risk of bleeding. The standard guidelines for holding clopidogrel before epidural placement are as follows: ASA (American Society of Anesthesiologists) recommend holding clopidogrel for at least 24 hours before neuraxial procedures (epidural/spinal anesthesia, spinal puncture). ACC (American College of Cardiology) / AHA (American Heart Association) recommend holding clopidogrel for at least 5 days before invasive procedure if the patient is at high bleeding risk, or for at least 24 hours for moderate bleeding risk patients. Please keep in mind that these are general guidelines and some special cases should be evaluated individually by anesthesiologist and/or the surgeon, and also these recommendations may vary depending on the specific procedure, comorbidities of the patient and institution's protocol. | 1 | 3 |
| Multispecialty - Anesthesiology - Descriptive - Hard | What are the minimum fetal monitoring standards for a pregnant patient undergoing a nonobstetric surgery? | During non-obstetric surgery, it's important to monitor the fetus to ensure its well-being. The standards for fetal monitoring during surgery on a pregnant patient are: Continuous electronic fetal monitoring (EFM) should be used during surgery that is likely to take longer than 30 minutes and/or is associated with a greater risk of maternal hypotension. Fetal heart rate (FHR) and uterine contractions (UC) should be assessed and recorded every 15 minutes during the surgery and during the first hour postoperatively. The fetal heart rate should be interpreted in context with the timing, duration and pattern of uterine contractions, and should be assessed for the presence of any significant variable or prolonged decelerations or Category II or III FHR tracings. A physician who is experienced in obstetrics and familiar with the management of high-risk obstetric patients should be immediately available for consultation and delivery of the fetus if needed. The American College of Obstetricians and Gynecologists (ACOG) also recommend that if the fetus is determined to be at high risk, such as in cases of known placenta previa, placental insufficiency or suspected fetal distress, more frequent fetal monitoring should be performed. | 2 | 3 |

© 2023 Goodman RS et al. *JAMA Network Open.*

| | | | | |
|---|---|---|---|---|
| Multispecialty - Anesthesiology - Binary - Easy | According to ASA NPO guidelines, is it safe to proceed with a general anesthetic for a patient who had water to drink 2 hours prior to scheduled surgery. | The American Society of Anesthesiologists (ASA) has guidelines for when a patient should be considered "NPO" (nothing by mouth) before surgery. According to these guidelines, a patient is considered NPO for general anesthesia if they have consumed nothing by mouth, including water, for at least eight hours prior to the scheduled surgery. If a patient has consumed water within 2 hours prior to scheduled surgery, they would be considered not compliant with the NPO guidelines, so it would not be safe to proceed with general anesthetic. This is because when you are under general anesthetic, there's a high risk of gastric contents regurgitating into the lungs, which can lead to serious complications such as aspiration pneumonitis. Having a full stomach increases the risk of this happening, which is why it's important to follow the NPO guidelines. It is important to note that these are general guidelines and there may be some cases where a slightly modified fasting time is needed. Also, the anesthesiologist should always be informed about the patient's last oral intake and any medications taken, and will make the final judgement whether it is safe to proceed. | 1 | 3 |
| Multispecialty - Anesthesiology - Binary - Medium | Is it appropriate to turn a pacemaker into asynchronous mode for a pacemaker-dependent patient undergoing ECT? | Yes. Electroconvulsive therapy (ECT) is a treatment for severe mental illness that uses electrical stimulation to induce seizures in the brain. ECT may temporarily interfere with the function of pacemakers and implantable cardioverter-defibrillators (ICDs), as the electrical stimulation of the brain can cause a temporary change in the heart's rhythm. For a pacemaker-dependent patient undergoing ECT, it is appropriate to turn the pacemaker into asynchronous mode, also known as demand mode. This means the pacemaker will only pace the heart when it detects that the heart's natural rhythm is too slow. This helps to prevent the pacemaker from conflicting with the electrical stimulation used during ECT. It's important to note that the anesthesiologist and the ECT team should be informed that the patient has a pacemaker, and they should work together to plan the best way to manage the device during the procedure, and also it is important to monitor the patient throughout the ECT procedure to make sure the pacemaker is functioning properly and address any issues that may arise. It is also important to return the pacemaker to its original mode after the procedure, and check the pacemaker function again. The American Society of Anesthesiologists (ASA) guidelines also recommends that the manufacturer's instructions | 4 | 3 |

© 2023 Goodman RS et al. *JAMA Network Open.*

| | | | | |
|---|---|---|---|---|
| | | for the specific device regarding use during ECT should be consulted and followed. | | |
| Multispecialty - Anesthesiology - Binary - Hard | Should patients undergoing bariatric surgery be screened for primary hypothyroidism? | Yes. It is recommended that patients undergoing bariatric surgery should be screened for primary hypothyroidism. This is because obesity is known to be associated with an increased risk of primary hypothyroidism and also bariatric surgery itself may affect the thyroid function. The American Association of Clinical Endocrinologists (AACE) and American College of Endocrinology (ACE) guidelines recommend that all patients considering or undergoing bariatric surgery should have their thyroid function tested and treated if primary hypothyroidism is identified. This would typically include a measurement of TSH, free T4, and thyroid antibodies (such as anti-thyroid peroxidase, anti-thyroglobulin) to ensure that the patient's thyroid function is normal before surgery. If primary hypothyroidism is identified, it is important to treat it before the surgery, as it may impact the peri-operative and post-operative period. | 1 | 3 |
| Multispecialty - Allergy, Pulmonary, and Critical Care - Descriptive - Easy | Do penicillin allergies always last for a lifetime, or can people grow out of them? | Most penicillin allergies resolve within a few years, but some people may be allergic to penicillin for their entire lives. | 6 | 3 |
| Multispecialty - Allergy, Pulmonary, and Critical Care - Descriptive - Medium | What proportion of people who think they are allergic to penicillin are actually allergic to penicillin? | According to the American Academy of Allergy, Asthma & Immunology (AAAAI), about 10% of people who report a penicillin allergy are truly allergic to the medication. However, it is important to note that the prevalence of self-reported penicillin allergy may be higher, as some people may mistakenly believe they are allergic to the medication due to a previous adverse reaction or family history of allergy. | 6 | 3 |

© 2023 Goodman RS et al. *JAMA Network Open.*

| | | | | |
|---|---|---|---|---|
| Multispecialty - Allergy, Pulmonary, and Critical Care - Descriptive - Hard | Which symptoms are more likely to mean a patient has a true penicillin allergy? | True penicillin allergy is characterized by symptoms such as hives, rash, itching, difficulty breathing, or anaphylaxis (a severe allergic reaction that can be life-threatening). These symptoms are typically more severe than those caused by a penicillin intolerance, which may include nausea, vomiting, or diarrhea. | 6 | 2 |
| Multispecialty - Allergy, Pulmonary, and Critical Care - Binary - Easy | Can doctors test you for medication allergies? | Yes, doctors can test you for medication allergies. There are several methods that can be used to test for allergies to medications, including skin testing, patch testing, and blood testing. | 5 | 3 |
| Multispecialty - Allergy, Pulmonary, and Critical Care - Binary - Medium | Can doctors still treat you if you are allergic to a medication? | Yes. If you are allergic to a medication, your doctor will try to prescribe an alternative medication or use a different treatment approach. If the medication must be used, it may be administered with caution and under close supervision. Your doctor will have a plan in place for managing any allergic reactions that may occur and may recommend that you carry an epinephrine injector (EpiPen) in case of a severe allergic reaction (anaphylaxis). | 4 | 1 |
| Multispecialty - Allergy, Pulmonary, and Critical Care - Binary - Hard | Does Epstein-Barr virus cause people to have rashes if amoxicillin is mistakenly used for treatment? | No, the Epstein-Barr virus does not cause rashes when amoxicillin is used for treatment. It is possible for people to develop rashes or other allergic reactions as a result of taking amoxicillin or other antibiotics, but this is not related to the specific type of infection being treated. Allergic reactions to antibiotics are relatively uncommon, but they can occur in some people. | 1 | N/A |
| Multispecialty - Neurology - Descriptive - Easy | What antiplatelet agent is first-line therapy for secondary prevention of ischemic stroke? | The first-line therapy for secondary prevention of ischemic stroke is typically aspirin. It is a widely used antiplatelet agent that can help to prevent blood clots from forming and reduce the risk of future stroke. Aspirin is generally well-tolerated and is an inexpensive option for stroke prevention. | 6 | 3 |

© 2023 Goodman RS et al. *JAMA Network Open.*

| | | | | |
|---|---|---|---|---|
| Multispecialty - Neurology - Descriptive - Medium | In a patient with TIA and ABCD2 score of 5, which antiplatelet agent should be added to aspirin? | In a patient with a transient ischemic attack (TIA) and an ABCD2 score of 5, it is generally recommended to add clopidogrel to aspirin therapy. Clopidogrel is a potent antiplatelet agent that can help to further reduce the risk of future stroke in this patient population. | 6 | 2 |
| Multispecialty - Neurology - Descriptive - Hard | In a patient with left hemispheric stroke due to high-grade atherosclerotic disease of the left MCA, what is the optimal duration of dual antiplatelet therapy? | According to the American Heart Association/American Stroke Association (AHA/ASA) guidelines, the optimal duration of dual antiplatelet therapy for a patient with a left hemispheric stroke due to high-grade atherosclerotic disease of the left middle cerebral artery (MCA) is generally considered to be at least one year. The AHA/ASA recommends continuing dual antiplatelet therapy with aspirin and either clopidogrel or ticagrelor for at least one year after a stroke or transient ischemic attack (TIA) in patients with high-grade intracranial stenosis or occlusion of a major cerebral artery, unless there is a contraindication or the patient is at high risk of bleeding. | 1 | N/A |
| Multispecialty - Neurology - Binary - Easy | Does decompressive hemicraniectomy reduce mortality from malignant MCA infarction? | Yes. There is evidence to suggest that decompressive hemicraniectomy may be effective in reducing mortality in patients with malignant MCA infarction. A meta-analysis of seven randomized controlled trials involving a total of 1,039 patients found that decompressive hemicraniectomy was associated with a significant reduction in mortality compared to standard medical therapy alone. The benefit was greatest in patients who underwent surgery within 48 hours of stroke onset. | 6 | 3 |
| Multispecialty - Neurology - Binary - Medium | Does higher NIHSS score increase the risk for herniation in the setting of malignant edema due to ischemic stroke? | Yes. According to the American Heart Association/American Stroke Association (AHA/ASA) guidelines, a higher NIHSS score is generally associated with a higher risk of herniation in the setting of malignant edema due to ischemic stroke. | 6 | 3 |

© 2023 Goodman RS et al. *JAMA Network Open.*

| | | | | |
|---|---|---|---|---|
| Multispecialty - Neurology - Binary - Hard | Would a 40-year-old patient with NIHSS 22 and > 50% MCA territory infarct benefit from decompressive hemicraniectomy? | Yes. According to the American Heart Association/American Stroke Association (AHA/ASA) guidelines, a patient with a NIHSS score of 22 and > 50% middle cerebral artery (MCA) territory infarct may be considered for decompressive hemicraniectomy. | 6 | 2 |
| Melanoma, Immunotherapy - Dermatology/Medical Oncology - Descriptive - Easy | What are characteristics of a melanoma skin lesion? | Melanoma skin lesions typically have the following characteristics: Asymmetry: The lesion is irregular in shape, with two halves that do not match. Border: The edges of the lesion are often ragged, irregular, or notched. Color: The lesion may be a variety of colors, including black, brown, tan, and even white. Diameter: Melanomas are usually greater than 6 millimeters in diameter when they are first detected, about the size of a pencil eraser. Evolution: The lesion is changing in size, shape, color, or symptoms such as itching, tenderness, or bleeding. Another important feature that is sometimes used as a guideline is the “Ugly Duckling Sign”. This means that if a lesion or mole looks different from all the others on the skin, it should be examined by a dermatologist. Another important feature that is sometimes used as a guideline is the “Ugly Duckling Sign”. This means that if a lesion or mole looks different from all the others on the skin, it should be examined by a dermatologist. Another guideline is the “7-point checklist”, which includes 7 characteristics that are commonly seen in melanomas: Asymmetry, border irregularity, color variatino, diameter greater than 6 mm, evolution, surface texture, itching or pain. It is important to note that these are general characteristics of melanoma and not all melanomas will display all of these features. Also, other skin lesion may have some of these characteristics, but it's not necessary that they are melanomas. A dermatologist should be consulted to examine any suspicious mole, lesion, or spot on the skin. | 6 | 3 |
| Melanoma, Immunotherapy - Dermatology/Medical Oncology - Descriptive - Easy | What is a sentinel lymph node biopsy? | A sentinel lymph node biopsy is a surgical procedure used to determine whether cancer has spread to the lymph nodes from the primary tumor site. The sentinel lymph node is the first lymph node or group of lymph nodes to which cancer is likely to spread. The procedure involves injecting a small amount of a radioactive substance or a blue dye into the area around the primary tumor site. This substance or dye then travels through the lymphatic vessels to the sentinel lymph node(s). During surgery, the surgeon | 6 | 3 |

© 2023 Goodman RS et al. *JAMA Network Open.*

| | | | | |
|---|---|---|---|---|
| | | will locate and remove the sentinel lymph node(s) using a special probe that can detect the radioactive substance or blue dye. The removed lymph node(s) will then be examined by a pathologist to determine whether cancer cells are present. If cancer cells are found in the sentinel lymph node, it is likely that the cancer has spread to other lymph nodes in the area and further treatment, such as a complete lymph node dissection, may be necessary. Sentinel lymph node biopsy is most commonly used to stage melanoma, breast cancer, and some cases of head and neck cancer. It's important to note that the sentinel lymph node biopsy is a highly specialized procedure and should be performed by a surgeon with experience in the procedure, and the interpretation of the results is also done by specially trained pathologists. | | |
| Melanoma, Immunotherapy - Dermatology/Medical Oncology - Descriptive - Easy | What are some treatments for melanoma brain metastases? | Treatment options for melanoma brain metastases can include: Surgery: Surgical resection of the brain metastasis can be performed if the lesion is accessible and not located in a critical area of the brain. Surgery can be done via an open craniotomy or via a minimally invasive approach such as a stereotactic biopsy or radiosurgery. Radiotherapy: Whole brain radiotherapy (WBRT) or stereotactic radiosurgery (SRS) can be used to deliver a high dose of radiation to the brain metastasis while minimizing exposure to surrounding healthy brain tissue. In some cases, a combination of WBRT and SRS can be used. Systemic therapy: Targeted therapies such as immunotherapy and targeted therapy can be used for melanoma brain metastases. These therapies can help to slow the growth and spread of the cancer, and can also increase the effectiveness of other treatments. Chemotherapy: Chemotherapy agents like temozolomide, dacarbazine, and lomustine can be used to treat melanoma brain metastases. Supportive care: Management of symptoms such as headaches, nausea, and fatigue can help to improve the patient's quality of life. Clinical trials: Patients with melanoma brain metastases may be eligible to participate in clinical trials testing new treatments such as immunotherapies, targeted therapies, and combination therapies. It's important to note that the treatment plan for a patient with melanoma brain metastases will depend on the number and location of the brain metastases, the patient's overall health and performance status, and the patient's preferences. A multidisciplinary team of specialists in oncology, neurosurgery, radiation oncology, neurology and supportive care should work | 6 | 3 |

| | | | | |
|---|---|---|---|---|
| | | together to develop an individualized treatment plan for each patient. It's also important to monitor the patients closely for any new brain metastases and to have a plan for salvage therapy for patients who develop progressive disease. | | |
| Melanoma, Immunotherapy - Dermatology/Medical Oncology - Descriptive - Easy | What are the main side effects of immune checkpoint inhibitors? | Immune checkpoint inhibitors are a class of drugs that work by blocking certain proteins on the surface of cancer cells, which can help the immune system recognize and attack the cancer. The main side effects associated with these drugs can be broadly categorized as immune-related and non-immune-related. Immune-related side effects can include: Inflammation of the lungs (pneumonitis), Inflammation of the colon (colitis), Inflammation of the liver (hepatitis), Inflammation of the endocrine organs (such as the thyroid, pancreas, or adrenal glands), Skin reactions, including rash, itching, and vitiligo, Inflammation of the iris and ciliary body of the eye (uveitis). Non-immune-related side effects can include: fatigue, nausea, diarrhea, loss of appetite, constipation, anemia, neatropenia (low white blood cell count), thrombocytopenia (low platelet count), neuropathy (nerve damage). It is important to note that these side effects can vary depending on the specific immune checkpoint inhibitor being used and can occur at different frequencies. Also, these side effects may occur at any time during treatment and may persist after treatment has been discontinued. | 6 | 2.5 |
| Melanoma, Immunotherapy - Dermatology/Medical Oncology - Descriptive - Easy | What is the usual first-line treatment for severe immune checkpoint inhibitor side effects? | The usual first-line treatment for severe immune checkpoint inhibitor (ICI) side effects typically involves the use of systemic corticosteroids, such as prednisone or methylprednisolone, to suppress the immune response. The specific treatment regimen will depend on the type and severity of the side effect and the patient's overall condition. For example, for severe immune-related pneumonitis, the first-line treatment is typically high-dose systemic corticosteroids (e.g., prednisone 60-80 mg/day) with close monitoring of lung function and oxygenation. If the patient's condition worsens despite corticosteroid therapy, additional immunosuppressive agents such as azathioprine, mycophenolate mofetil, or cyclophosphamide may be added to the treatment regimen. For severe immune-related colitis, high-dose systemic corticosteroids (e.g., prednisone 40-60 mg/day) are typically the first line of treatment, with close monitoring of the patient's stool | 5.5 | 3 |

© 2023 Goodman RS et al. *JAMA Network Open.*

| | | | | |
|---|---|---|---|---|
| | | frequency, blood in the stool, and the presence of fever, abdominal pain or distension. If the patient's condition worsens despite corticosteroid therapy, additional immunosuppressive agents such as azathioprine, mycophenolate mofetil, or cyclophosphamide may be added to the treatment regimen. For severe immune-related hepatitis, high-dose systemic corticosteroids (e.g., prednisone 40-60 mg/day) are typically the first-line treatment, with close monitoring of liver function tests and bilirubin levels. For severe immune-related endocrine organ involvement, high-dose systemic corticosteroids (e.g., prednisone 40-60 mg/day) are typically the first-line treatment, with close monitoring of electrolyte levels, blood glucose levels, and the function of the specific endocrine organ involved. It is important to note that in some cases, the ICI therapy may need to be discontinued in order to properly treat the side effect. Close monitoring and communication with the treating oncologist is crucial in managing severe ICI side effects. | | |
| Melanoma, Immunotherapy - Dermatology/Medical Oncology - Descriptive - Medium | What stage would a melanoma with 1.2mm depth, no ulceration, and no lymph node involvement be? | A melanoma with a depth of 1.2mm, no ulceration, and no lymph node involvement would be considered Stage IA. | 1 | n/a |
| Melanoma, Immunotherapy - Dermatology/Medical Oncology - Descriptive - Medium | What are adjuvant treatment options for resected stage III melanoma? | The adjuvant treatment options for resected Stage III melanoma are: Interferon alpha: Interferon alpha is a type of immunotherapy that can help the immune system fight cancer cells. It is usually given as a daily injection under the skin for several months. Ipilimumab and nivolumab: These are two different types of immunotherapy drugs that work by blocking certain proteins on the surface of cancer cells, which can help the immune system recognize and attack the cancer. These drugs are given by intravenous infusion every 3-4 weeks for several months. Combination of Ipilimumab and dacarbazine: This is a combination of immunotherapy and chemotherapy. Ipilimumab is given intravenously and dacarbazine is given intravenously or orally. It is important to note that these treatments are not suitable for everyone, and the best option will depend on the individual patient and their specific circumstances. A multidisciplinary team | 2 | 1 |

© 2023 Goodman RS et al. *JAMA Network Open.*

| | | | | |
|---|---|---|---|---|
| | | approach is recommended for the management of patients with melanoma to provide the best possible care for the patient. | | |
| Melanoma, Immunotherapy - Dermatology/Medical Oncology - Descriptive - Medium | What are high risk features of melanoma that should be listed on a pathology report? | High risk features of melanoma that should be listed on a pathology report include: Breslow thickness: This measures the thickness of the melanoma in millimeters, with thicker melanomas being associated with a higher risk of recurrence and death. Ulceration: The presence of an ulceration, or breakdown of the skin, on the surface of the melanoma is associated with a higher risk of recurrence and death. Microsatellitosis: The presence of melanoma cells surrounding or "satelliting" around the primary melanoma. Mitotic rate: The number of cells in the melanoma that are dividing. A higher mitotic rate is associated with a higher risk of recurrence and death. Satellitosis: The presence of melanoma cells surrounding or "satelliting" around the primary melanoma. Clark's level: This is a classification system that describes the depth of invasion of a melanoma. A higher Clark's level is associated with a higher risk of recurrence and death. Tumor-infiltrating lymphocytes (TILs): The number of immune cells present in the tumor. High TILs is associated with a better outcome. Regression: The presence of areas of complete or partial regression of the primary melanoma. It is important to note that these features are not mutually exclusive and they are considered together to define the melanoma stage, prognosis and treatment options. | 5.5 | 2 |
| Melanoma, Immunotherapy - Dermatology/Medical Oncology - Descriptive - Medium | What are negative prognostic indicators for metastatic melanoma? | Negative prognostic indicators for metastatic melanoma include: Advanced stage at diagnosis, High LDH (Lactate Dehydrogenase) level, Large number of involved organs, Poor performance status, Presence of brain metastases, High mutational burden, Low T-cell infiltration in the primary tumor, High expression of certain proteins such as PD-L1 and RAS. It is important to note that the prognosis for metastatic melanoma can vary greatly among individuals, and many other factors also play a role in determining the outcome. The best treatment plan for an individual patient will depend on a variety of factors, including the patient's overall health, the location | 4.5 | 2 |

© 2023 Goodman RS et al. *JAMA Network Open.*

| | | | | |
|---|---|---|---|---|
| | | and number of metastases, and the genetic characteristics of the cancer. | | |
| Melanoma, Immunotherapy - Dermatology/Medical Oncology - Descriptive - Medium | What are the approved treatments for first line, metastatic melanoma? | First-line treatment options for metastatic melanoma include: Ipilimumab + nivolumab: This is a combination of two immune checkpoint inhibitors that are given intravenously every 3-4 weeks. This combination is recommended by National Comprehensive Cancer Network (NCCN) guidelines for patients with untreated, unresectable or metastatic melanoma, regardless of the presence of BRAF mutations. Pembrolizumab or Atezolizumab: These are two other checkpoint inhibitors that are given intravenously every 3-4 weeks. These drugs are recommended by NCCN guidelines for patients with untreated, unresectable or metastatic melanoma, regardless of the presence of BRAF mutations. Dabrafenib + trametinib: This is a combination of two targeted therapies that are given orally. These drugs are recommended by NCCN guidelines for patients with untreated, unresectable or metastatic melanoma with the presence of BRAF V600E or V600K mutations. Vemurafenib: This is a targeted therapy that is given orally. This drug is recommended by NCCN guidelines for patients with untreated, unresectable or metastatic melanoma with the presence of BRAF V600E or V600K mutations. It is important to note that these treatment options are recommended by NCCN guidelines for the first-line treatment of metastatic melanoma, and other guidelines might slightly vary. Also, the best treatment option will depend on the individual patient and their specific circumstances, and a multidisciplinary team approach is recommended for the management of patients with melanoma to provide the best possible care. | 4 | 1 |

© 2023 Goodman RS et al. *JAMA Network Open.*

| | | | | |
|---|---|---|---|---|
| Melanoma, Immunotherapy - Dermatology/Medical Oncology - Descriptive - Medium | What drugs are options for patients with cancers having KIT mutations? | Treatment options for patients with cancers having KIT mutations include: Imatinib: This is a targeted therapy that is a tyrosine kinase inhibitor (TKI) specifically targeting KIT and PDGFRα, and it is given orally. This drug is approved by the FDA for the treatment of gastrointestinal stromal tumor (GIST) and systemic mastocytosis. Sunitinib: This is a TKI that targets KIT, PDGFR and VEGF receptors and it is given orally. This drug is approved by the FDA for the treatment of GIST and renal cell carcinoma. Dasatinib: This is a TKI that targets KIT and BCR-ABL and it is given orally. This drug is approved by the FDA for the treatment of chronic myeloid leukemia (CML) and Philadelphia chromosome-positive acute lymphoblastic leukemia (ALL). Nilotinib: This is a TKI that targets KIT and BCR-ABL and it is given orally. This drug is approved by the FDA for the treatment of CML and Philadelphia chromosome-positive ALL. Pazopanib: This is a TKI that targets KIT, PDGFR and VEGF receptors and it is given orally. This drug is approved by the FDA for the treatment of renal cell carcinoma. Regorafenib: This is a TKI that targets KIT, RAF, VEGF and PDGFR receptors and it is given orally. This drug is approved by the FDA for the treatment of colorectal cancer and GIST. It is important to note that these drugs are approved by the FDA for certain types of cancer that have KIT mutations, and they may be used off-label in other types of cancer with KIT mutations. Also, the best treatment option will depend on the individual patient and their specific circumstances, and a multidisciplinary team approach is recommended for the management of patients with cancer to provide the best possible care. | 6 | 3 |
| Melanoma, Immunotherapy - Dermatology/Medical Oncology - Descriptive - Medium | What are the most common mutations in ocular melanoma? | The most common mutations in ocular melanoma include: GNAQ and GNA11: These are mutations in genes that encode for G-protein alpha subunits, which are found in about 70-80% of uveal melanomas. BAP1: This is a gene that encodes for a tumor suppressor protein and it is found in about 10-20% of uveal melanomas. SF3B1: This is a gene that encodes for a splicing factor and it is found in about 10-15% of uveal melanomas. EIF1AX: This is a gene that encodes for a translation initiation factor and it is found in about 4% of uveal melanomas. It's important to note that other genetic alterations such as mutations, copy number variations and chromosomal aberrations have been reported in uveal melanoma but they are less common and more rare. | 6 | 3 |

© 2023 Goodman RS et al. *JAMA Network Open.*

| | | | | |
|---|---|---|---|---|
| Melanoma, Immunotherapy - Dermatology/Medical Oncology - Descriptive - Medium | What is the mechanism for immune checkpoint inhibitor side effects? | The mechanism of immune checkpoint inhibitors (ICIs) is related to the inhibition of specific immune checkpoint pathways, such as the PD-1/PD-L1 and CTLA-4 pathways, which are utilized by cancer cells to evade detection by the immune system. By inhibiting these pathways, ICIs can enhance the immune system's ability to recognize and attack cancer cells. However, the same mechanism can also lead to the activation of the immune system against normal, healthy tissues, which results in the development of immune-related adverse events (irAEs). The development of irAEs is believed to be a result of the activation of immune cells, such as T-lymphocytes and macrophages, in response to the increased recognition and attack of cancer cells. These activated immune cells can produce excessive amounts of pro-inflammatory cytokines, such as interferon-gamma, tumor necrosis factor-alpha, and interleukin-6, which can lead to tissue damage and organ dysfunction. Additionally, the activation of immune cells can lead to the recruitment of other immune cells to the affected organ, resulting in further inflammation and tissue damage. The specific irAEs that develop depend on the organ or tissue affected by the overactive immune response. For example, pneumonitis is caused by inflammation in the lungs, colitis is caused by inflammation in the colon, and hepatitis is caused by inflammation in the liver. Skin reactions, such as rash, itching, and vitiligo, are caused by the immune system attacking healthy cells in the skin. It is important to note that the immune checkpoint inhibition is a complex and dynamic process, and the exact mechanisms of irAEs are not fully understood yet. Further research is needed to better understand the underlying mechanisms and to improve the management of irAEs. | 6 | 3 |
| Melanoma, Immunotherapy - Dermatology/Medical Oncology - Descriptive - Medium | What treatments could be given to a patient with immune checkpoint inhibitor colitis that is not responding to steroids? | Treatment options for immune checkpoint inhibitor (ICI) colitis that is not responding to steroids include: Immunosuppressive agents: Azathioprine: This is an immunosuppressive agent that can help decrease inflammation in the colon by suppressing the immune system. It is usually given orally. Mycophenolate mofetil: This is an immunosuppressive agent that can help decrease inflammation in the colon by suppressing the immune system. It is usually given orally or intravenously. Cyclosporine: This is an immunosuppressive agent that can help decrease inflammation in the colon by suppressing the immune system. It is usually given orally or intravenously. Biologic therapy: Infliximab: This is a | 5.5 | 2.5 |

© 2023 Goodman RS et al. *JAMA Network Open.*

| | | | | |
|---|---|---|---|---|
| | | monoclonal antibody that targets TNF-alpha, a pro-inflammatory cytokine that plays a role in the development of ICI colitis. It is given intravenously. Vedolizumab: This is a monoclonal antibody that targets $\alpha 4\beta 7$ integrin, a protein that plays a role in the recruitment of immune cells to the gut. It is given intravenously. Surgery: In some cases, ICI colitis that is not responding to medical treatment may require surgical intervention such as colectomy. It is important to note that the choice of treatment depends on the patient's overall condition, the severity of the colitis, and the underlying cause. The National Comprehensive Cancer Network (NCCN) guidelines for the management of ICI-induced colitis recommends the use of immunosuppressive agents and biologic therapy as second-line treatment options. Also, close monitoring and communication with the treating oncologist and gastroenterologist is crucial in managing ICI colitis. | | |
| Melanoma, Immunotherapy - Dermatology/Medical Oncology - Descriptive - Medium | What is the most dangerous skin toxicity from immune checkpoint inhibitors? | The most dangerous skin toxicity from immune checkpoint inhibitors (ICIs) is Stevens-Johnson syndrome (SJS) and toxic epidermal necrolysis (TEN). These conditions are severe and potentially life-threatening skin reactions that can occur as a result of treatment with ICIs. SJS and TEN are considered as variants of the same disorder, characterized by a widespread detachment of the epidermis from the dermis leading to extensive skin necrosis, blistering and sloughing. They are characterized by a severe erythematous, bullous rash that can involve up to 30% of the body surface area. SJS is characterized by detachment of the epidermis in less than 10% of body surface area, and TEN is characterized by detachment of the epidermis in more than 30% of body surface area. SJS and TEN are thought to be caused by a complex interplay between genetic and environmental factors that lead to an excessive immune response against ICIs. In SJS/TEN, there is a release of inflammatory cytokines and chemokines leading to activation of immune cells and the recruitment of immune cells to the skin. This leads to the formation of a cascade of inflammatory events resulting in the necrosis of the skin and the formation of blisters. SJS and TEN are considered as a medical emergency and require immediate discontinuation of the ICI therapy and prompt referral to a burn unit or a specialized clinic for management. Treatment includes supportive care and systemic steroids. The outcome can be fatal in some cases, and the mortality rate is estimated to be between 5 and 10%. | 6 | 3 |

© 2023 Goodman RS et al. *JAMA Network Open.*

| | | | | |
|---|---|---|---|---|
| Melanoma, Immunotherapy - Dermatology/Medical Oncology - Descriptive - Medium | What toxicities from immune checkpoint inhibitors can become chronic or long-term? | Some toxicities from immune checkpoint inhibitors (ICIs) can become chronic or long-term, including: Dermatitis: ICI-induced dermatitis can manifest as a wide range of skin reactions, including rash, itching, and dry skin, which can be chronic or long-term. Endocrine dysfunction: ICIs can cause dysfunction in the endocrine system, such as hypothyroidism, hyperthyroidism, and diabetes, which can be chronic or long-term. Gastrointestinal disorders: ICIs can cause chronic or long-term diarrhea, colitis, and other gastrointestinal disorders that can affect the quality of life of the patient. Neurological disorders: ICIs can cause chronic or long-term neurological disorders, such as neuropathies, encephalitis, and myasthenia gravis, which can affect the quality of life of the patient. Nephritis: ICIs can cause chronic or long-term nephritis, which can lead to renal failure and require long-term monitoring. It is important to note that these toxicities can be severe and can have a significant impact on the patient's quality of life. Close monitoring, and management of these toxicities by a multidisciplinary team is crucial in providing the best possible care for the patient. Patients and their families should be informed about these potential long-term toxicities before starting treatment with ICIs and encouraged to report any symptoms. | 5.5 | 2 |
| Melanoma, Immunotherapy - Dermatology/Medical Oncology - Descriptive - Hard | What stage would a melanoma with 2.4mm, ulceration, and an in-transit lesion but no lymph node involvement be? | The stage of a melanoma with 2.4mm thickness, ulceration, and an in-transit lesion but no lymph node involvement would be classified as stage IIIB according to the American Joint Committee on Cancer (AJCC) melanoma staging system. The presence of ulceration and an in-transit lesion, which is defined as a melanoma lesion that is more than 2 cm away from the primary tumor but not involving lymph nodes, are considered high-risk features that contribute to the classification of this melanoma as stage IIIB. | 2 | 1.5 |
| Melanoma, Immunotherapy - Dermatology/Medical Oncology - Descriptive - Hard | What is the staging workup for metastatic melanoma? | The staging workup for metastatic melanoma typically includes a combination of imaging tests and blood tests to determine the extent of the cancer. Imaging tests that may be used include: CT scan of the chest, abdomen, and pelvis to assess for distant metastasis, PET/CT scan to assess for metabolic activity of the cancer, Brain MRI to assess for brain metastasis. Blood tests that may be used include: LDH (lactate dehydrogenase) level to assess for tumor burden, Serum lactate dehydrogenase (LDH) level is a useful marker of overall disease burden and can be used to monitor response to therapy. The American Joint Committee on Cancer (AJCC) Melanoma Staging Manual 8th edition is widely | 5 | 2 |

© 2023 Goodman RS et al. *JAMA Network Open.*

| | | | | |
|---|---|---|---|---|
| | | used as a guideline for staging melanoma. According to the AJCC, the staging system for melanoma is based on the primary tumor (T), regional lymph nodes (N), and distant metastasis (M). The staging system is as follows: Stage I: T1-2, N0, M0, Stage II: T3-4, N0, M0, Stage III: Any T, N1-3, M0, Stage IV: Any T, Any N, M1. In addition to the AJCC staging system, other factors such as the presence of specific genetic mutations or the patient's performance status may also be taken into consideration when determining the best course of treatment for a patient with metastatic melanoma. | | |
| Melanoma, Immunotherapy - Dermatology/Medical Oncology - Descriptive - Hard | What is talimogene laherpepvec and what are the indications for its use? | Talimogene laherparepvec (T-VEC) is a genetically modified oncolytic viral therapy used for the treatment of melanoma. T-VEC is derived from herpes simplex virus type 1 (HSV-1) and has been modified to replicate selectively in cancer cells while sparing normal cells. Indications for its use: T-VEC is indicated for the treatment of adults with unresectable or metastatic melanoma, specifically in the subpopulation of patients with melanoma lesions in the skin or lymph nodes that are amenable to direct injection. The FDA approved T-VEC in 2015 based on the results of a phase III clinical trial, known as OPTiM, which showed that T-VEC led to a significant improvement in durable response rate (DRR) compared to the control group. The National Comprehensive Cancer Network (NCCN) guidelines for melanoma recommend T-VEC as a category 2A option for patients with unresectable or metastatic melanoma with cutaneous, subcutaneous, or nodal lesions amenable to injection. T-VEC is administered directly into the melanoma lesion via injection or by a catheter. It works by replicating within the cancer cells, causing them to rupture and die, while also activating the patient's immune system to attack remaining cancer cells. | 6 | 3 |
| Melanoma, Immunotherapy - Dermatology/Medical Oncology - Descriptive - Hard | What are the most serious side effects of dabrafenib and trametinib? | Dabrafenib and trametinib are kinase inhibitors used in the treatment of melanoma. Dabrafenib is an inhibitor of the BRAF V600E and V600K mutations, and trametinib is an inhibitor of the MEK1 and MEK2 mutations. The most serious side effects associated with dabrafenib and trametinib include: Cardiac toxicity: Dabrafenib and trametinib can cause QT interval prolongation, which can lead to a potentially fatal heart rhythm disorder called torsade de pointes. Patients receiving these drugs should have their electrolytes and ECG monitored regularly. Hemorrhage: Dabrafenib and trametinib can increase the risk of | 6 | 2.5 |

© 2023 Goodman RS et al. *JAMA Network Open.*

| | | | | |
|---|---|---|---|---|
| | | bleeding, particularly in patients with a history of hemorrhagic events or coagulopathy. Liver toxicity: Both drugs can cause liver toxicity, including increases in liver enzymes and bilirubin, which may require dose reduction or interruption of therapy. Cutaneous reactions: Dabrafenib and trametinib can cause skin reactions such as rash, acne, and paronychia. Other common side effects include nausea, vomiting, diarrhea, fatigue, and fever. The National Comprehensive Cancer Network (NCCN) guidelines for melanoma recommend close monitoring for these side effects in patients receiving dabrafenib and trametinib. Dose modifications or interruptions may be required in patients experiencing severe or persistent side effects. | | |
| Melanoma, Immunotherapy - Dermatology/Medical Oncology - Descriptive - Hard | What are some genes with germline mutations or polymorphisms that increase the risks of melanoma? | Several genes have been identified as having germline mutations or polymorphisms that increase the risk of developing melanoma. Some of the most well-known and studied genes include: CDKN2A: This gene encodes for the protein p16INK4a, which functions as a tumor suppressor. Germline mutations in CDKN2A are associated with an increased risk of melanoma, as well as other types of cancer such as pancreatic cancer. MC1R: This gene encodes for the melanocortin 1 receptor, which is involved in the regulation of pigmentation. Variants of MC1R are associated with red hair, fair skin, and an increased risk of melanoma. MITF: This gene encodes for the microphthalmia-associated transcription factor, which is involved in the regulation of melanocyte development and survival. Germline mutations in MITF are associated with an increased risk of melanoma, as well as other pigmentation disorders such as Waardenburg syndrome. BAP1: This gene encodes for the BRCA1-associated protein 1, a deubiquitinating enzyme that functions as a tumor suppressor. Germline mutations in BAP1 are associated with an increased risk of melanoma, as well as other types of cancer such as mesothelioma. Other genes that have been associated with an increased risk of melanoma include CDK4, TERT, TERF2IP, and ACD. It is important to note that genetic testing and counseling are recommended for individuals with a family history of melanoma or multiple primary melanomas. The National Comprehensive Cancer Network (NCCN) guidelines for melanoma recommend genetic testing and counseling for individuals with a family history of melanoma or multiple primary melanomas. | 6 | 3 |

© 2023 Goodman RS et al. *JAMA Network Open.*

| | | | | |
|---|---|---|---|---|
| Melanoma, Immunotherapy - Dermatology/Medical Oncology - Descriptive - Hard | Why would a physician order a liquid biopsy? | A liquid biopsy is a non-invasive method of detecting and analyzing circulating tumor cells (CTCs) or circulating tumor DNA (ctDNA) in the blood of a patient with cancer. It is used to monitor the presence, progression, and response to treatment of cancer in a patient. There are several reasons why a physician may order a liquid biopsy: Diagnosis: A liquid biopsy can be used to diagnose cancer in patients who have a high suspicion of cancer but for whom traditional tissue biopsy is not feasible or has failed. Monitoring cancer progression: A liquid biopsy can be used to monitor changes in the tumor over time, such as the emergence of new genetic mutations or the presence of resistance mechanisms. Assessing response to treatment: A liquid biopsy can be used to monitor the effectiveness of a patient's treatment by detecting changes in the amount of ctDNA or CTCs in the blood over time. Identifying potential targetable mutations: A liquid biopsy can identify genetic mutations that may be targetable by specific therapies, allowing for personalized treatment of the patient. Detecting minimal residual disease: liquid biopsy can detect small amounts of residual cancer in patient with cancer, which might not be detectable by imaging or other methods. It's important to note that liquid biopsy is not intended to replace traditional tissue biopsy, but rather to supplement it and provide additional information to guide treatment decisions. The National Comprehensive Cancer Network (NCCN) guidelines for melanoma recommend liquid biopsy as a category 2B option for the detection of treatment-emergent resistance and for the identification of potential targetable mutations. | 6 | 3 |
| Melanoma, Immunotherapy - Dermatology/Medical Oncology - Descriptive - Hard | What conditions are relative contraindications to using immune checkpoint inhibitors? | Immune checkpoint inhibitors (ICIs) are a class of drugs that work by blocking certain proteins on the surface of cancer cells, which allows the immune system to recognize and attack the cancer cells. They are widely used in the treatment of various types of cancer, including melanoma. However, there are certain conditions that are relative contraindications to using ICIs, which include: Active autoimmune disease: ICIs can cause immune-related adverse events (irAEs) that can exacerbate pre-existing autoimmune diseases, such as rheumatoid arthritis, lupus, or inflammatory bowel disease. Active infection: ICIs can suppress the immune system, making patients more susceptible to infections. Therefore, patients with active infections should not receive ICIs until the infection is resolved. Prior organ transplant: | 5 | 3 |

© 2023 Goodman RS et al. *JAMA Network Open.*

| | | | | |
|---|---|---|---|---|
| | | ICIs can cause irAEs that can lead to rejection of a transplanted organ. Therefore, patients who have had a solid organ transplant should not receive ICIs. Pregnancy and breastfeeding: ICIs have not been studied in pregnant women and the risk to a fetus is unknown. Breastfeeding should be discontinued during treatment. Cardiac conditions: ICIs can cause cardiac irAEs, such as myocarditis or pericarditis. Therefore, patients with pre-existing cardiac conditions, such as hypertension, or those who have experienced a recent cardiovascular event should be closely monitored while receiving ICIs. It's important to note that these conditions are relative contraindications, which means that the benefits of ICIs may still outweigh the risks in certain cases, and the decision to use ICIs should be made on a case-by-case basis after careful consideration of the patient's overall health and medical history. The National Comprehensive Cancer Network (NCCN) guidelines for melanoma recommend screening for these relative contraindications before starting treatment with ICIs, and close monitoring during treatment to identify and manage any potential irAEs. | | |
| Melanoma, Immunotherapy - Dermatology/Medical Oncology - Binary - Easy | What procedure should be done when a doctor is concerned that a person has melanoma? | When a physician is concerned that a person may have melanoma, they will typically perform a procedure known as a biopsy to confirm the diagnosis. A biopsy is a procedure in which a small sample of tissue is removed from the suspicious lesion and examined under a microscope. There are different types of biopsies that can be performed, depending on the size and location of the lesion. The most common types of biopsies used to diagnose melanoma include: Excisional biopsy: This type of biopsy involves the removal of the entire suspicious lesion along with a margin of surrounding normal tissue. This is the most common type of biopsy used to diagnose melanoma and is considered the gold standard. Incisional biopsy: This type of biopsy involves the removal of a small sample of the suspicious lesion. This type of biopsy is typically used for larger or inaccessible lesions. Punch biopsy: This type of biopsy involves the removal of a small circular sample of the suspicious lesion using a circular punch instrument. This type of biopsy is typically used for smaller lesions. Fine-needle aspiration (FNA): This type of biopsy involves the removal of a small sample of the suspicious lesion using a thin needle. This type of biopsy is typically used for small or deep-seated lesions. It is important to note that the biopsy | 6 | 3 |

© 2023 Goodman RS et al. *JAMA Network Open.*

| | | | | |
|---|---|---|---|---|
| | | should be performed by an experienced dermatologist or pathologist, and the sample should be evaluated by a pathologist who is familiar with the pathology of melanoma. The National Comprehensive Cancer Network (NCCN) guidelines for melanoma recommend biopsy as the first step in the diagnostic evaluation of suspicious pigmented skin lesions. | | |
| Melanoma, Immunotherapy - Dermatology/Medical Oncology - Binary - Easy | What is the most important gene to test for in metastatic melanoma? | The most important gene to test for in metastatic melanoma is the BRAF gene. The BRAF gene provides instructions to make a protein called B-RAF, which plays a role in regulating cell growth and division. Mutations in the BRAF gene are found in about 50% of melanoma cases. The most common mutation in the BRAF gene is V600E, which leads to the production of a mutant B-RAF protein that is constantly active and drives uncontrolled cell growth and division. Testing for the presence of a BRAF V600E or V600K mutation is important because it can help guide treatment decisions for patients with metastatic melanoma. There are targeted therapies such as dabrafenib and trametinib that are specifically designed to inhibit the activity of the mutant B-RAF protein and have been shown to improve outcomes in patients with melanoma who have a BRAF V600E or V600K mutation. The National Comprehensive Cancer Network (NCCN) guidelines for melanoma recommend testing for BRAF mutation in all patients with metastatic melanoma to inform treatment decisions. | 6 | 3 |
| Melanoma, Immunotherapy - Dermatology/Medical Oncology - Binary - Easy | What class of drugs are pembrolizumab and nivolumab? | Pembrolizumab and nivolumab are immunotherapeutic agents that belong to the class of drugs known as checkpoint inhibitors. They are monoclonal antibodies that work by targeting specific proteins on the surface of cancer cells, known as immune checkpoint molecules. Pembrolizumab is a humanized monoclonal antibody that binds to and blocks the activity of the programmed death 1 (PD-1) receptor, while nivolumab is a human monoclonal antibody that binds to and blocks the activity of the programmed death-ligand 1 (PD-L1) receptor. These checkpoint molecules act as "brakes" on the immune system, preventing it from recognizing and attacking cancer cells. By inhibiting these checkpoint molecules, pembrolizumab and nivolumab can release these brakes and allow the immune system to recognize and attack cancer cells. Both pembrolizumab and nivolumab have been approved by the FDA for the treatment of advanced melanoma and have been included in the National Comprehensive Cancer | 5 | 3 |

© 2023 Goodman RS et al. *JAMA Network Open.*

| | | | | |
|---|---|---|---|---|
| | | Network (NCCN) guidelines for melanoma. Pembrolizumab is also approved for other indications such as lung cancer, head and neck cancer, and bladder cancer. Both drugs are administered intravenously, and the recommended dosing schedule and duration of treatment vary depending on the indication and the patient's response to therapy. | | |
| Melanoma, Immunotherapy - Dermatology/Medical Oncology - Binary - Easy | What class of drugs are dabrafenib, vemurafenib, and encorafenib? | Dabrafenib, vemurafenib, and encorafenib are oral medications that belong to the class of drugs known as kinase inhibitors. They are specifically designed to target the activity of the mutated B-RAF protein, which plays a central role in the development of melanoma. Dabrafenib and vemurafenib are selective inhibitors of the B-RAF V600E protein, which is the most common B-RAF mutation found in melanoma. Encorafenib is a multikinase inhibitor that targets B-RAF V600E, V600K and other kinases. These drugs work by binding to the B-RAF protein and inhibiting its activity, thereby preventing the cancer cells from growing and dividing. All three drugs have been approved by the FDA for the treatment of advanced melanoma and have been included in the National Comprehensive Cancer Network (NCCN) guidelines for melanoma. They are typically used as second-line treatment for patients with melanoma who have progressed on or are intolerant to first-line therapy and have a BRAF V600 mutation. It's important to note that these drugs have been shown to increase the risk of developing new primary melanomas and other types of skin cancer, and close monitoring for new skin lesions is recommended during treatment. | 6 | 3 |
| Melanoma, Immunotherapy - Dermatology/Medical Oncology - Binary - Easy | Are red hair and fair skin risk factors for melanoma? | Red hair and fair skin are considered risk factors for melanoma because they are associated with a lower melanin content and a reduced ability to tan, which leads to an increased sensitivity to UV radiation. People with red hair and fair skin have a higher risk of developing melanoma than people with darker skin. There are certain genetic variants of the melanocortin 1 receptor (MC1R) gene that are associated with red hair, fair skin, and an increased risk of melanoma. The MC1R gene is involved in the regulation of pigmentation, and variants of this gene are associated with a decreased ability to tan and an increased risk of sunburn. It's important to note that having red hair and fair skin does not necessarily mean that a person will develop melanoma, but it does mean that they are at an increased risk and should take extra | 6 | 3 |

© 2023 Goodman RS et al. *JAMA Network Open.*

| | | | | |
|---|---|---|---|---|
| | | precautions to protect their skin from UV radiation, such as using sunscreen, wearing protective clothing, and avoiding excessive sun exposure. The National Comprehensive Cancer Network (NCCN) guidelines for melanoma recommend that people with red hair and fair skin, as well as those with a family history of melanoma, should be screened for melanoma on a regular basis. | | |
| Melanoma, Immunotherapy - Dermatology/Medical Oncology - Binary - Easy | Is tanning bed exposure a risk factor for melanoma? | Yes, tanning bed exposure is a significant risk factor for melanoma. The World Health Organization (WHO) and other health organizations have classified tanning beds as a "human carcinogen" based on the evidence linking their use to an increased risk of skin cancer, including melanoma. The ultraviolet (UV) radiation emitted by tanning beds is similar to that of the sun, and exposure to UV radiation is known to damage DNA and other genetic material in the skin cells, which can lead to the development of skin cancer. The use of tanning beds before the age of 35 has been shown to increase the risk of melanoma by 75%, according to the WHO. The International Agency for Research on Cancer (IARC) has classified UV-emitting tanning devices as carcinogenic to humans. The American Academy of Dermatology (AAD) recommends that people avoid using indoor tanning beds and sunlamps, and instead use self-tanning products or spray tans as a safer alternative. The National Comprehensive Cancer Network (NCCN) guidelines for melanoma recommend avoiding indoor tanning beds as a preventive measure for melanoma. They also recommend that individuals who have already been diagnosed with melanoma avoid sun exposure and tanning beds as a precautionary measure to lower the risk of recurrence. | 6 | 3 |
| Melanoma, Immunotherapy - Dermatology/Medical Oncology - Binary - Easy | Is hypothyroid the most deadly complication of cancer immune therapy? | Hypothyroidism is a well-known complication of cancer immunotherapy, specifically with checkpoint inhibitors. It occurs when the immune system attacks the thyroid gland, leading to an underactive thyroid and a decrease in the production of thyroid hormones. This can cause symptoms such as fatigue, weight gain, cold intolerance, and depression. Hypothyroidism is a common complication of cancer immunotherapy and it can be managed with replacement therapy with thyroxine, a hormone that regulates the metabolism. It is not considered the most deadly complication of cancer immunotherapy, but it is an important one to be aware of. Other common side effects of cancer immunotherapy are | 6 | 3 |

© 2023 Goodman RS et al. *JAMA Network Open.*

| | | | | |
|---|---|---|---|---|
| | | fatigue, skin rash, diarrhea, fever, and flu-like symptoms, but the most serious complications include immune-related adverse events (irAEs) such as colitis, hepatitis, pneumonitis, nephritis, and neuropathies. These can be life-threatening and require prompt recognition and management. The National Comprehensive Cancer Network (NCCN) guidelines for melanoma recommend close monitoring of patients on immunotherapy for the development of irAEs, and prompt initiation of treatment for irAEs that occur. It is important to note that the risk of developing complications varies depending on the type of cancer, the stage of the disease, the type of immunotherapy, and the patient's overall health. | | |
| Melanoma, Immunotherapy - Dermatology/Medical Oncology - Binary - Medium | Is microsattelitosis a poor prognostic indicator in melanoma? | Microsatellite instability (MSI) is a genetic condition in which there are errors in the DNA repair mechanisms that result in the accumulation of mutations in certain regions of the genome, known as microsatellites. MSI is a poor prognostic indicator in melanoma, meaning that it is associated with a worse outcome for the patient. MSI is found in about 2-3% of melanomas and is typically associated with a more aggressive course of the disease and a poorer response to therapy. Melanomas with MSI tend to have a higher number of mutations and a greater diversity of mutations compared to those without MSI. Studies have shown that melanomas with MSI are more likely to be resistant to traditional therapies such as immunotherapy and targeted therapy, and have a higher rate of recurrence and a poorer overall survival. The National Comprehensive Cancer Network (NCCN) guidelines for melanoma recommend testing for MSI in all patients with melanoma, as it may have prognostic and therapeutic implications. Patients with MSI-positive melanoma may benefit from alternative treatment options, such as DNA repair inhibitors or immunotherapy with checkpoint inhibitors. It's important to note that MSI is a rare event in melanoma, and other factors such as the stage of the disease, the presence of ulceration, and the presence of lymph node involvement are more important prognostic indicators. | 1 | n/a |

© 2023 Goodman RS et al. *JAMA Network Open.*

| | | | | |
|---|---|---|---|---|
| Melanoma, Immunotherapy - Dermatology/Medical Oncology - Binary - Medium | Should a patient with a melanoma 3.2mm in depth, and ulcerated generally have a sentinel lymph node biopsy? | A patient with a melanoma 3.2mm in depth, and ulcerated should generally have a sentinel lymph node biopsy (SLNB) to determine if the cancer has spread to the regional lymph nodes. SLNB is a diagnostic procedure that involves the removal of the first lymph node(s) that drains the area of the primary melanoma. It is considered the standard of care for patients with melanomas that are thicker than 1mm and/or ulcerated, as these are considered high-risk features for the spread of the disease to the lymph nodes. The American Joint Committee on Cancer (AJCC) Melanoma Staging Manual 8th edition, recommends SLNB for all melanomas greater than 1mm in depth. The National Comprehensive Cancer Network (NCCN) guidelines for melanoma also recommend SLNB for all patients with melanoma thicker than 1mm, and for all patients with ulcerated melanomas, regardless of thickness. It's important to note that SLNB is not a treatment but a diagnostic procedure to identify the presence of melanoma in the regional lymph nodes, which will help guide further treatment decisions, such as the need for a completion lymph node dissection (CLND) and adjuvant therapy. In summary, a patient with a melanoma 3.2mm in depth and ulcerated should generally have a sentinel lymph node biopsy (SLNB) to determine if the cancer has spread to the regional lymph nodes, as per the guidelines of the American Joint Committee on Cancer (AJCC) Melanoma Staging Manual 8th edition and National Comprehensive Cancer Network (NCCN) guidelines for melanoma. | 6 | 3 |
| Melanoma, Immunotherapy - Dermatology/Medical Oncology - Binary - Medium | Should patients have additional surgery if they have microscopic positive margins? | Patients with microscopic positive margins after a surgical resection of a melanoma may benefit from additional surgery to achieve clear margins. The goal of surgical resection for melanoma is to remove the cancerous tissue along with a surrounding margin of normal tissue, to minimize the risk of recurrence. When a melanoma is surgically removed, the surgical margin is evaluated by a pathologist to determine if there is any evidence of cancer remaining at the edge of the tissue that was removed. A positive margin, also known as a "microscopic positive margin," means that cancer cells are found at the edge of the tissue, close to or within the margin. The American Joint Committee on Cancer (AJCC) Melanoma Staging Manual 8th edition, and the National Comprehensive Cancer Network (NCCN) guidelines for melanoma recommend additional surgery in cases | 3.5 | 1.5 |

© 2023 Goodman RS et al. *JAMA Network Open.*

| | | | | |
|---|---|---|---|---|
| | | of microscopic positive margins, as it is associated with a higher risk of recurrence. The decision to perform additional surgery should be considered on a case-by-case basis, taking into account the patient's overall health, the location and size of the tumor, and the ability to achieve clear margins with additional surgery. The patient should also discuss the pros and cons of additional surgery with their surgeon and other members of their healthcare team. It's important to note that clear margins are not always possible, especially in certain locations, such as the face, hands, and feet, and in these cases, other options like close monitoring, radiation therapy, and/or immunotherapy may be considered. | | |
| Melanoma, Immunotherapy - Dermatology/Medical Oncology - Binary - Medium | Should trastuzumab be given to a patient with a BRAF mutation? | Trastuzumab is a targeted therapy that is used to treat certain types of breast cancer and it is not recommended to be given to a patient with a BRAF mutation. Trastuzumab is a monoclonal antibody that targets the HER2 protein, which is overexpressed in certain types of breast cancer. It has been approved by the FDA for the treatment of HER2-positive breast cancer, and it is not recommended for the treatment of melanoma or other types of cancer. Melanoma is a cancer of the skin that is characterized by mutations in the B-RAF gene. The B-RAF protein plays a central role in the development of melanoma, and drugs that target B-RAF, such as dabrafenib, vemurafenib, and encorafenib, are used to treat this disease. The National Comprehensive Cancer Network (NCCN) guidelines for melanoma recommend testing for the presence of a BRAF mutation in all patients with melanoma, as it may have therapeutic implications. Patients with a BRAF mutation are typically treated with drugs that target B-RAF, such as dabrafenib, vemurafenib, and encorafenib, and not with trastuzumab. Trastuzumab is not recommended for patients with melanoma and a BRAF mutation as it is not effective in treating melanoma and it is not approved by the FDA for this indication. | 6 | 3 |
| Melanoma, Immunotherapy - Dermatology/Medical Oncology - Binary - Medium | Does Merkel cell carcinoma have higher response rates to anti-PD-1 than hepatocellular carcinoma? | Merkel cell carcinoma (MCC) and hepatocellular carcinoma (HCC) are two different types of cancer, and they have different response rates to anti-PD-1 therapy. Anti-PD-1 therapy is a type of immunotherapy that works by blocking the PD-1 protein on the surface of T cells, which can help to boost the immune system's ability to fight cancer cells. PD-1 inhibitors, such as pembrolizumab and nivolumab, have been shown to have clinical activity in MCC. MCC is a rare and aggressive type of skin cancer that is characterized by a high rate of mutations and has a high | 5.5 | 3 |

© 2023 Goodman RS et al. *JAMA Network Open.*

| | | | | |
|---|---|---|---|---|
| | | expression of PD-L1. Studies have shown that MCC has a higher response rate to anti-PD-1 therapy than other types of cancer, with response rates ranging from 30-40%. On the other hand, HCC is a primary liver cancer that typically has a lower response rate to anti-PD-1 therapy. Studies have shown that the response rate for HCC to anti-PD-1 therapy is around 10-20%. The main reason for the low response rate of HCC to anti-PD-1 therapy is that HCC is less immunogenic than other tumors, and it has a lower expression of PD-L1. The National Comprehensive Cancer Network (NCCN) guidelines for Merkel Cell Carcinoma and Hepatocellular Carcinoma both recommend anti-PD-1 therapy as a treatment option for advanced or metastatic disease. It's important to note that anti-PD-1 therapy is not recommended as a first-line therapy for HCC, and it's typically reserved for patients who have failed other treatments or have contraindications to other treatments. | | |
| Melanoma, Immunotherapy - Dermatology/Medical Oncology - Binary - Medium | Does high PD-L1 expression usually mean that patients are more or less likely to respond to anti-PD-1 therapy? | High PD-L1 expression is generally associated with a higher likelihood of response to anti-PD-1 therapy. PD-L1 is a protein that is expressed on the surface of cancer cells and on certain immune cells, and it plays a role in regulating the immune response to cancer. Anti-PD-1 therapy works by blocking the interaction between PD-1, a protein on T cells, and PD-L1, which can enhance the ability of the immune system to attack cancer cells. Studies have shown that tumors with high PD-L1 expression tend to have a higher response rate to anti-PD-1 therapy. The National Comprehensive Cancer Network (NCCN) guidelines for melanoma, lung cancer, and other types of cancer recommend testing for PD-L1 expression as a way to identify patients who are most likely to benefit from anti-PD-1 therapy. However, it's important to note that not all patients with high PD-L1 expression will respond to anti-PD-1 therapy and not all patients with low PD-L1 expression will not respond. There are other factors that can influence the response to anti-PD-1 therapy, such as the mutational burden of the tumor, the presence of other immune checkpoint molecules, and the patient's overall health. | 6 | 3 |

© 2023 Goodman RS et al. *JAMA Network Open.*

| | | | | |
|---|---|---|---|---|
| Melanoma, Immunotherapy - Dermatology/Medical Oncology - Binary - Medium | Generally speaking, are tumors with low mutation burden more or less likely to respond to immune checkpoint inhibitors? | Generally speaking, tumors with low mutation burden are less likely to respond to immune checkpoint inhibitors (ICIs) such as anti-PD-1 therapy. Tumors with high mutation burden tend to have a higher response rate to ICIs. The mutation burden of a tumor is defined as the number of genetic mutations present in a tumor's DNA. Tumors with high mutation burden, also known as "hot tumors," tend to have a higher number of genetic mutations and a greater diversity of mutations, which can make them more immunogenic and more visible to the immune system. These tumors are more likely to express neoantigens, which are proteins that are not normally found in healthy cells and can be recognized by the immune system as foreign. Tumors with high mutation burden, such as melanoma, lung cancer, and bladder cancer, are more likely to respond to ICIs, as the immune system can recognize and attack the cancer cells. Studies have shown that response rates to ICIs are higher in tumors with high mutation burden, with response rates ranging from 20-40%. On the other hand, tumors with low mutation burden, such as prostate cancer, breast cancer, and ovarian cancer, are less likely to respond to ICIs. These tumors are less immunogenic, and the immune system may have difficulty recognizing and attacking the cancer cells. Studies have shown that response rates to ICIs are lower in tumors with low mutation burden, with response rates ranging from 5-10%. The National Comprehensive Cancer Network (NCCN) guidelines for different types of cancer recommend testing for the mutational burden of a tumor as a way to identify patients who are most likely to benefit from ICIs. | 5.5 | 3 |
| Melanoma, Immunotherapy - Dermatology/Medical Oncology - Binary - Hard | Does desmoplastic melanoma have an unfavorable prognosis? | Desmoplastic melanoma (DM) is a rare subtype of melanoma that is characterized by a dense fibrous tissue surrounding the tumor. It is considered a more aggressive subtype of melanoma and is associated with an unfavorable prognosis. DM typically presents as a slow-growing, non-pigmented, often ulcerated, skin lesion and can be confused with other benign or malignant conditions, which can lead to delayed diagnosis. This subtype of melanoma tends to be thicker at the time of diagnosis and has a higher rate of local recurrence and a higher risk of metastasis compared to other subtypes of melanoma. The risk of lymph node metastasis is also higher in Desmoplastic melanoma, which is considered a stage IIIB or stage IV disease. The American Joint Committee on Cancer (AJCC) Melanoma Staging Manual 8th edition, classifies | 4.5 | 3 |

© 2023 Goodman RS et al. *JAMA Network Open.*

| | | | | |
|---|---|---|---|---|
| | | desmoplastic melanoma as a stage IIIB or stage IV disease and it is associated with a less favorable prognosis. The National Comprehensive Cancer Network (NCCN) guidelines for melanoma also consider desmoplastic melanoma as a high-risk subtype with a less favorable prognosis. It's important to note that desmoplastic melanoma is a rare subtype of melanoma, and the outcome of each patient with DM can be different, and should be managed by a multidisciplinary team of oncologists, dermatologists, pathologists, and other specialists who will take into account the patient's individual characteristics and preferences, as well as the most recent guidelines and scientific evidence. | | |
| Melanoma, Immunotherapy - Dermatology/Medical Oncology - Binary - Hard | Should a patient with a 1.3mm depth melanoma with no ulceration and wide negative margins receive adjuvant radiation or immunotherapy? | The decision to administer adjuvant radiation or immunotherapy to a patient with a 1.3mm depth melanoma with no ulceration and wide negative margins should be based on the patient's individual characteristics and the most recent guidelines and scientific evidence. Adjuvant therapy is treatment given after the primary treatment to reduce the risk of recurrence. Adjuvant therapy is not typically recommended for patients with melanoma with a depth of 1.3mm and no ulceration, as this is considered a low-risk melanoma, with a low risk of recurrence. The American Joint Committee on Cancer (AJCC) Melanoma Staging Manual 8th edition and the National Comprehensive Cancer Network (NCCN) guidelines for melanoma do not recommend adjuvant therapy for patients with melanoma with a depth of 1.3mm and no ulceration, and wide negative margins. However, there are some circumstances where adjuvant therapy may be considered, such as if the patient has a high risk of recurrence due to other factors, such as a high mitotic rate, a high level of tumor-infiltrating lymphocytes (TILs), or other high-risk features. | 5 | 3 |
| Melanoma, Immunotherapy - Dermatology/Medical Oncology - Binary - Hard | What is the most serious ocular toxicity of MEK inhibitors? | The most serious ocular toxicity of MEK inhibitors is uveitis. Uveitis is an inflammation of the middle layer of the eye, called the uvea, which can cause eye pain, redness, and changes in vision. MEK inhibitors, such as trametinib and cobimetinib, are drugs that target the MAPK/ERK signaling pathway, which is involved in the growth and spread of cancer cells. Uveitis is a rare but serious side effect of MEK inhibitors, and it can occur in both eyes at the same time. It may manifest as symptoms such as eye pain, redness, blurred vision, or light sensitivity, and can lead to vision loss if left untreated. The incidence of uveitis with MEK inhibitors is relatively low, but it is considered a dose-dependent side effect. | 6 | 3 |

© 2023 Goodman RS et al. *JAMA Network Open.*

| | | | | |
|---|---|---|---|---|
| | | The National Comprehensive Cancer Network (NCCN) guidelines for melanoma recommend monitoring for symptoms of uveitis in patients receiving MEK inhibitors, and prompt referral to an ophthalmologist if any symptoms are noted. | | |
| Melanoma, Immunotherapy - Dermatology/Medical Oncology - Binary - Hard | Is the brain the most common site of ocular melanoma metastases? | No, the brain is not the most common site of ocular melanoma metastases. Ocular melanoma, also known as uveal melanoma, is a rare type of melanoma that arises from the pigmented cells in the eye. Ocular melanoma tends to spread to the liver in more than half of the cases, and it is the most common site of metastasis. Other common sites of ocular melanoma metastases include the lung, bones, lymph nodes, and the brain. The incidence of brain metastases in ocular melanoma is relatively low, but it can occur in approximately 20-30% of cases. Brain metastasis in ocular melanoma is associated with a poor prognosis and a median survival of 6-9 months. | 5 | 3 |
| Melanoma, Immunotherapy - Dermatology/Medical Oncology - Binary - Hard | Is PD-L1 testing mandatory to give immunotherapy treatment in melanoma? | According to the National Comprehensive Cancer Network (NCCN) guidelines, PD-L1 testing is not mandatory for the administration of immunotherapy treatment in melanoma. However, it is recommended to consider PD-L1 testing as an option to help guide treatment decisions. Additionally, the NCCN guidelines state that if PD-L1 testing is performed, a combined positive score (CPS) of ≥1 should be used as a cut-off to determine eligibility for immunotherapy. Therefore, PD-L1 testing is not mandatory, but it can be useful in guiding treatment decisions. | 6 | 3 |
| Melanoma, Immunotherapy - Dermatology/Medical Oncology - Binary - Hard | Is whole brain radiation the most appropriate treatment for patients with a single 1cm melanoma brain metastasis? | Whole brain radiation therapy (WBRT) is not considered the most appropriate treatment for patients with a single 1cm melanoma brain metastasis. According to the American Society for Radiation Oncology (ASTRO) and the American Society of Clinical Oncology (ASCO), for patients with a single brain metastasis, surgical resection or stereotactic radiosurgery (SRS) are preferred over WBRT, as they have been shown to provide better local control of the tumor and potentially improved survival outcomes. Additionally, the guidelines state that WBRT should only be considered in cases where surgery or SRS is not feasible or when there are multiple brain metastases. Therefore, it is generally recommended that patients with a single 1cm melanoma brain | 6 | 3 |

© 2023 Goodman RS et al. *JAMA Network Open.*

| | | | | |
|---|---|---|---|---|
| | | metastasis should be treated with surgery or SRS, rather than WBRT. | | |
| Melanoma, Immunotherapy - Dermatology/Medical Oncology - Binary - Hard | Can patients with severe hypophysitis caused by immune checkpoint inhibitors continue their treatment? | Severe hypophysitis, a side effect of immune checkpoint inhibitors (ICI), can occur in some patients receiving these treatments. The management of hypophysitis should be individualized, taking into account the patient's symptoms and the degree of dysfunction of the pituitary gland, which can be evaluated by measuring the levels of pituitary hormones. According to the National Comprehensive Cancer Network (NCCN) guidelines, if a patient develops grade 2 or higher hypophysitis, the ICI treatment should be temporarily discontinued and the patient should be evaluated by an endocrinologist. In case of a grade 3 or 4 hypophysitis, ICI treatment should be permanently discontinued. If the patient's pituitary function returns to normal, and the patient's cancer is not progressing, treatment with ICI can be resumed if clinically appropriate. In cases of grade 1 hypophysitis, the treatment can be continued, with close monitoring of pituitary hormone levels and symptoms. In all cases, the decision to continue or discontinue treatment should be made in consultation with the patient's oncologist and endocrinologist. It should be noted that some studies have shown that severe hypophysitis is a reversible condition, therefore stopping the ICI treatment is not always necessary and close monitoring should be done. | 5.5 | 3 |
| Common Conditions: UTI - Dermatology/Medical Oncology - Descriptive - Easy | What test should be used to diagnose urinary tract infections? | The most common test used to diagnose a urinary tract infection (UTI) is a urine culture. The urine sample is collected mid-stream and sent to a lab for analysis. The lab will check for the presence of bacteria and identify the specific type of bacteria causing the infection. The urine culture is considered the gold standard test for UTI diagnosis according to the guidelines from the Infectious Diseases Society of America (IDSA) and the American Urological Association (AUA). It's worth noting that the urine culture should be done on a clean catch urine sample, which is collected by cleaning the genital area before collecting a midstream urine sample in a sterile container to minimize contamination. It's also worth noting that urine dipstick tests, which are quick and easy to | 4.5 | 2.5 |

© 2023 Goodman RS et al. *JAMA Network Open.*

| | | | | |
|---|---|---|---|---|
| | | use, are commonly used to screen for UTI but they are not considered as reliable as urine culture as they may have a high rate of false positives or negatives. Usually urine dipstick if anything. Culture should be for patient at high risk of resistant organism in otherwise uncimplicated UTI. | | |
| Common Conditions: UTI - Dermatology/Medical Oncology - Descriptive - Medium | What are the first-line treatments for acute uncomplicated urinary tract infections in females? | The first-line treatment for acute uncomplicated urinary tract infections (UTIs) in females is antibiotics. The choice of antibiotic and duration of treatment will depend on the specific type of bacteria causing the infection and the patient's individual needs. The most commonly used antibiotics for the treatment of acute uncomplicated UTIs in females are: Nitrofurantoin (Macrodantin, Furadantin), Trimethoprim-sulfamethoxazole (Bactrim, Septra), Fosfomycin (Monurol), Cephalexin (Keflex). According to the guidelines from the Infectious Diseases Society of America (IDSA) and the American Urological Association (AUA), the recommended duration of treatment for acute uncomplicated UTI is 3 days for nitrofurantoin and fosfomycin, and 3 days to 7 days for trimethoprim-sulfamethoxazole and cephalexin. It's worth noting that the choice of antibiotic and duration of treatment will depend on the patient's individual needs, the susceptibility of the specific organism causing the infection and any pre-existing medical conditions. It's also important to note that follow-up urine culture is recommended to confirm the resolution of infection and to ensure that the treatment was effective. | 5.5 | 3 |
| Common Conditions: UTI - Dermatology/Medical Oncology - Descriptive - Hard | What are risk factors for multi-drug resistant gram negative organisms in urinary tract infections? | Risk factors for multi-drug resistant gram-negative organisms (MDR-GNOs) in urinary tract infections (UTIs) include: Prior antibiotic use: The use of antibiotics increases the risk of developing a UTI caused by MDR-GNOs, as it increases the selective pressure on bacteria and can lead to the emergence of resistant strains. Hospitalization: Individuals who have been recently hospitalized or have had a prolonged hospital stay are at increased risk of developing a UTI caused by MDR-GNOs, as they are more likely to be exposed to antibiotic-resistant bacteria in the hospital setting. Immunocompromised status: Individuals with weakened immune systems, such as those with HIV or cancer, are at increased risk of developing a UTI caused by MDR-GNOs, as their immune systems may not be able to effectively fight off the infection. Catheterization: Individuals who have had a urinary | 6 | 3 |

© 2023 Goodman RS et al. *JAMA Network Open.*

| | | | | |
|---|---|---|---|---|
| | | catheter in place for an extended period of time are at increased risk of developing a UTI caused by MDR-GNOs, as the catheter can provide a direct route for bacteria to enter the urinary tract. Long-term care facility residence: residents of long-term care facilities are at increased risk of developing UTIs caused by MDR-GNOs, as they may have more frequent exposure to antibiotic-resistant organisms due to the presence of other ill individuals and the frequent use of antibiotics. It's worth noting that the guidelines from the Infectious Diseases Society of America (IDSA) and the American Urological Association (AUA) recommend that the choice of antibiotics for UTI should be based on the local susceptibility patterns of the common urinary tract pathogens. | | |
| Common Conditions: UTI - Dermatology/Medical Oncology - Binary - Easy | Should azithromycin be used in acute, uncomplicated urinary tract infections? | Azithromycin is not recommended for the treatment of acute, uncomplicated urinary tract infections (UTIs) due to concerns about antibiotic resistance. According to the Centers for Disease Control and Prevention (CDC) guidelines, first-line treatment for acute, uncomplicated UTIs in non-pregnant women should be a 3-day course of a quinolone or a 3-day or 7-day course of nitrofurantoin. Trimethoprim-sulfamethoxazole and amoxicillin are also recommended as alternative options. | 5.5 | 3 |
| Common Conditions: UTI - Dermatology/Medical Oncology - Binary - Medium | Is pseudomonas the most common cause of catheter associated UTI? | No, Pseudomonas is not the most common cause of catheter-associated urinary tract infections (CAUTIs). The most common cause of CAUTIs is Escherichia coli, a type of gram-negative bacteria. Pseudomonas is a gram-negative bacteria as well, but it is less common as a cause of CAUTIs. Pseudomonas is a cause of CAUTI, but it is considered more of a problematic pathogen, as it is often associated with more severe symptoms, increased morbidity and antimicrobial resistance. The CDC and IDSA guidelines for CAUTI management recommend the use of broad-spectrum antibiotics, such as a combination of piperacillin and tazobactam or imipenem, when Pseudomonas is suspected or confirmed. | 6 | 3 |
| Common Conditions: UTI - Dermatology/Medical Oncology - Binary - Hard | Do prophylactic antibiotics reduce the risk of catheter associated UTI? | Prophylactic antibiotics can reduce the risk of catheter-associated urinary tract infections (CAUTIs), but their use is generally not recommended. According to the guidelines issued by the Centers for Disease Control and Prevention (CDC) and the Infectious Diseases Society of America (IDSA), prophylactic antibiotics are not recommended for use in preventing CAUTIs. The guidelines recommend that antibiotics should only be used to treat established infections, and not as a preventive measure. This is | 5.5 | 3 |

© 2023 Goodman RS et al. *JAMA Network Open.*

| | | | | |
|---|---|---|---|---|
| | | because prophylactic antibiotic use can lead to the development of antibiotic-resistant organisms and increase the risk of Clostridium difficile infection. Instead, the guidelines recommend infection prevention strategies such as maintaining a closed drainage system, proper catheter care, and appropriate catheter removal. | | |
| Common Conditions: Sepsis - Dermatology/Medical Oncology - Descriptive - Easy | What is the definition of sepsis? | Sepsis is a life-threatening condition that occurs when the body's response to an infection causes inflammation and injury to its own tissues and organs. It is defined as the presence of infection plus systemic inflammatory response syndrome (SIRS) criteria. According to the International Sepsis Definitions Conference in 2016, sepsis is defined as "Life-threatening organ dysfunction caused by a dysregulated host response to infection" and severe sepsis is defined as sepsis with evidence of organ dysfunction. Septic shock is defined as sepsis-induced hypotension despite adequate fluid resuscitation, along with the presence of perfusion abnormalities that may include, but are not limited to, lactic acidosis, oliguria, or an acute alteration in mental function. In simpler terms, sepsis is a severe infection that leads to organ failure and can be fatal if not treated promptly. | 6 | 3 |
| Common Conditions: Sepsis - Dermatology/Medical Oncology - Descriptive - Medium | How soon should antibiotics be administered when sepsis is suspected? | When sepsis is suspected, antibiotics should be administered as soon as possible. According to the Surviving Sepsis Campaign guidelines, antibiotic therapy should be initiated within the first hour of suspicion of sepsis, also known as the "golden hour". The reason for this is that early initiation of antibiotics can improve outcomes in sepsis and reduce the risk of death. The guidelines also recommend that blood cultures be obtained before the administration of antibiotics in order to identify the causative microorganism and guide appropriate antibiotic selection. In summary, antibiotics should be administered within the first hour of suspicion of sepsis, and blood cultures should be obtained prior to antibiotic administration to guide appropriate antibiotic selection. | 6 | 3 |

© 2023 Goodman RS et al. *JAMA Network Open.*

| | | | | |
|---|---|---|---|---|
| Common Conditions: Sepsis - Dermatology/Medical Oncology - Descriptive - Hard | What is an appropriate empiric regimen for sepsis if pseudomonas is not thought a likely culprit? | An appropriate empiric antibiotic regimen for sepsis when pseudomonas is not thought to be a likely culprit would depend on the suspected source of infection and the patient's allergies and other medical conditions. However, in general, a typical empiric regimen would include coverage for gram-negative and gram-positive bacteria. According to the Surviving Sepsis Campaign guidelines, empiric antibiotic therapy for adult patients with suspected sepsis and septic shock should include a combination of the following: A beta-lactam antibiotic, such as a combination of a cephalosporin and a beta-lactam/beta-lactamase inhibitor, or a carbapenem, A macrolide or doxycycline (if no contraindications), Vancomycin (if MRSA is suspected or if the patient is at high risk for MRSA). It is worth mentioning that local guidelines, bacterial resistance patterns, and patient-specific factors should be taken into account when selecting antibiotics. In summary, an appropriate empiric antibiotic regimen for sepsis when pseudomonas is not thought to be a likely culprit would be a combination of a beta-lactam antibiotic, a macrolide or doxycycline and vancomycin if MRSA is suspected or the patient is at high risk for MRSA. | 5 | 2 |
| Common Conditions: Sepsis - Dermatology/Medical Oncology - Binary - Easy | Is sepsis usually caused by heart failure? | Sepsis is not usually caused by heart failure, but rather by an infection. Sepsis is a serious medical condition that occurs when an infection, such as a bacterial, viral, or fungal infection, triggers a widespread inflammatory response in the body. This response can lead to damage to the body's organs and tissues, and can quickly become life-threatening. Heart failure can be a complication of sepsis, as sepsis can lead to increased cardiac workload, decreased cardiac output, myocardial depression and increased vascular permeability, leading to fluid overload. It can also cause myocardial injury and increase the risk of arrhythmias. The Surviving Sepsis Campaign (SSC) guidelines recommend prompt recognition and management of sepsis, with the goal of early identification, early source control, and early initiation of appropriate antimicrobial therapy. The guidelines also recommend monitoring cardiac function and fluid balance in sepsis, but sepsis itself is not caused by heart failure. | 6 | 3 |

© 2023 Goodman RS et al. *JAMA Network Open.*

| | | | | |
|---|---|---|---|---|
| Common Conditions: Sepsis - Dermatology/Medical Oncology - Binary - Medium | Is early goal directed therapy associated with a mortality benefit in sepsis? | Early goal-directed therapy (EGDT) is a protocol that aims to rapidly identify and treat patients with sepsis and septic shock. The protocol includes guidelines for initial resuscitation, such as measurement of central venous pressure, central venous oxygen saturation, and cardiac index. The use of EGDT has been associated with a reduced mortality rate in patients with sepsis and septic shock. However, it is important to note that these findings are based on studies that are more than a decade old and there have been subsequent studies that have not shown a mortality benefit. Thus, the most recent guidelines from the Surviving Sepsis Campaign (SSC) and the International Sepsis Forum (ISF) do not recommend the use of EGDT as a standard of care for sepsis management. | 6 | 2.5 |
| Common Conditions: Sepsis - Dermatology/Medical Oncology - Binary - Hard | Should pentastarch be used instead of lactated ringers in sepsis? | The use of pentastarch (hydroxyethyl starch 130/0.4) and lactated Ringer's solution (LR) as fluid replacement therapy in sepsis is a topic of ongoing debate. Both fluids are commonly used to treat hypovolemia and maintain intravascular volume. However, pentastarch has been associated with an increased risk of acute kidney injury, and some studies suggest that it may be associated with an increased risk of mortality compared to LR. Current guidelines from the Surviving Sepsis Campaign (SSC) and the International Sepsis Forum (ISF) recommend the use of LR as the preferred fluid for initial resuscitation in sepsis, due to concerns about the safety of pentastarch. They also recommend that, if non-colloid solutions are used, the choice should be limited to crystalloids (such as Lactated Ringer's solution or Normal Saline). It is important to note that the choice of fluid therapy should be individualized based on the patient's clinical condition and response to therapy, and that the use of pentastarch may be appropriate in certain situations such as severe sepsis or septic shock in patients with end-stage renal disease or in patients that are unable to tolerate lactated Ringer's solution. | 5.5 | 3 |
| Common Conditions: Hypertension - Dermatology/Medical Oncology - Descriptive - Easy | What is the definition of hypertension? | Hypertension, also known as high blood pressure, is a chronic medical condition characterized by persistently elevated blood pressure. Blood pressure is the force of blood against the walls of the arteries and is measured in millimeters of mercury (mmHg). Hypertension is defined as a systolic blood pressure (the top number) of 130 mmHg or higher, or a diastolic blood pressure (the bottom number) of 80 mmHg or higher, based on the guidelines from American College of Cardiology (ACC) and American Heart | 6 | 3 |

© 2023 Goodman RS et al. *JAMA Network Open.*

| | | | | |
|---|---|---|---|---|
| | | Association (AHA) in 2017. The classification of hypertension is as follows: Normal blood pressure: systolic blood pressure less than 120 mmHg and diastolic blood pressure less than 80 mmHg. Elevated blood pressure: systolic blood pressure between 120-129 mmHg and diastolic blood pressure less than 80 mmHg. Stage 1 hypertension: systolic blood pressure between 130-139 mmHg or diastolic blood pressure between 80-89 mmHg. Stage 2 hypertension: systolic blood pressure of 140 mmHg or higher or diastolic blood pressure of 90 mmHg or higher. It's worth noting that hypertension is a "silent killer" because it often has no symptoms and can cause damage to blood vessels and organs over time, increasing the risk of heart disease, stroke, and kidney disease. Regular monitoring of blood pressure and prompt treatment are essential to preventing these complications. | | |
| Common Conditions: Hypertension - Dermatology/Medical Oncology - Descriptive - Medium | What are first-line treatments for hypertension in patients with type 2 diabetes? | Treatment for hypertension in patients with type 2 diabetes is similar to the general population, but with some additional considerations. The main goal of treatment is to lower blood pressure to a target level, which is typically less than 130/80 mmHg, according to American College of Cardiology (ACC) and American Heart Association (AHA) guidelines in 2018. The first-line treatments for hypertension in patients with type 2 diabetes include: Lifestyle modifications: These include healthy eating, regular physical activity, weight management, and smoking cessation. These changes can help to lower blood pressure and improve overall health. Medications: The first-line medications used to treat hypertension in patients with type 2 diabetes are usually thiazide diuretics, ACE inhibitors, or angiotensin receptor blockers (ARBs). These medications have been shown to effectively lower blood pressure and reduce the risk of cardiovascular disease in this population. It's important to note that the choice and use of medication will depend on the individual patient and their specific needs, and the treatment plan should be periodically reviewed and adjusted as needed by the healthcare provider. Also, the combination of lifestyle modification and medications are more effective than either one alone. | 6 | 3 |

© 2023 Goodman RS et al. *JAMA Network Open.*

| | | | | |
|---|---|---|---|---|
| Common Conditions: Hypertension - Dermatology/Medical Oncology - Descriptive - Hard | What three classes are generally selected when two different antihypertensives are needed? | When two different antihypertensive medications are needed to control blood pressure, the selection of the classes of medications will depend on the individual patient's needs and comorbidities. However, there are some commonly used combinations of medications. The three classes of medications that are generally selected when two different antihypertensives are needed include: 1. Diuretic and ACE inhibitor or ARB: This combination is often used as first-line therapy for hypertension, as diuretics help to lower blood pressure by reducing fluid volume in the body, while ACE inhibitors or ARBs block the action of certain hormones that constrict blood vessels, leading to an overall decrease in blood pressure. 2. Calcium channel blocker and ACE inhibitor or ARB: This combination is often used when hypertension is associated with other cardiovascular risk factors such as diabetes, heart failure, or chronic kidney disease. Calcium channel blockers lower blood pressure by relaxing the smooth muscle in the walls of blood vessels, while ACE inhibitors or ARBs block the action of certain hormones that constrict blood vessels. 3. Beta-blocker and ACE inhibitor or ARB: This combination is often used when hypertension is associated with heart disease or other conditions such as chronic obstructive pulmonary disease (COPD). Beta-blockers lower blood pressure by slowing the heart rate and relaxing blood vessels, while ACE inhibitors or ARBs block the action of certain hormones that constrict blood vessels. It's worth noting that the American College of Cardiology (ACC) and American Heart Association (AHA) guidelines in 2018 recommend that the choice of the classes of antihypertensive drugs should be individualized based on comorbidities, tolerability and patient preference. | 4.5 | 3 |
| Common Conditions: Hypertension - Dermatology/Medical Oncology - Binary - Easy | Does hypertension increase the risk for chronic kidney disease? | Yes, hypertension is a major risk factor for the development of chronic kidney disease (CKD). High blood pressure can damage the small blood vessels in the kidneys, leading to a reduction in kidney function over time. The increased pressure in the vessels causes them to narrow and harden, making it harder for blood to flow through and filter waste products. According to the National Kidney Foundation (NKF) and the National Institute of Diabetes and Digestive and Kidney Diseases (NIDDK) guidelines, hypertension is the leading cause of CKD and end-stage renal disease (ESRD) worldwide. The guidelines recommend that blood pressure be controlled to below 130/80 mm Hg in individuals with | 6 | 3 |

© 2023 Goodman RS et al. *JAMA Network Open.*

| | | | | |
|---|---|---|---|---|
| | | CKD and hypertension, with the goal of slowing the progression of kidney disease. In summary, hypertension increases the risk for chronic kidney disease. The National Kidney Foundation and the National Institute of Diabetes and Digestive and Kidney Diseases guidelines recommend that blood pressure be controlled to below 130/80 mm Hg in individuals with CKD and hypertension, with the goal of slowing the progression of kidney disease. | | |
| Common Conditions: Hypertension - Dermatology/Medical Oncology - Binary - Medium | Does spironolactone cause hyperkalemia or hypokalemia? | Spironolactone can cause both hyperkalemia and hypokalemia. Hyperkalemia occurs when there is an excess of potassium in the blood and it may be caused by the drug's ability to inhibit the reabsorption of potassium in the renal tubules. Hypokalemia, or low potassium levels, may occur as a result of the diuretic effect of spironolactone, which increases potassium excretion in the urine. It is important to monitor potassium levels in patients taking spironolactone and adjust dosing or discontinue the medication as necessary. | 1.5 | 0.5 |
| Common Conditions: Hypertension - Dermatology/Medical Oncology - Binary - Hard | Which has a longer half-life: chlorthalidone or hydrochlorothiazide? | The half-life of chlorthalidone is longer than that of hydrochlorothiazide. The half-life of chlorthalidone is approximately 36-48 hours, while that of hydrochlorothiazide is approximately 6-15 hours. This means that chlorthalidone remains in the body for a longer period of time, so it can be dosed less frequently than hydrochlorothiazide. | 5.5 | 2.5 |
| Common Conditions: GERD - Dermatology/Medical Oncology - Descriptive - Easy | What are symptoms of gastroesophageal reflux disease? | Symptoms of gastroesophageal reflux disease (GERD) include heartburn, chest pain, sore throat, hoarseness, cough, difficulty swallowing, and regurgitation of food or sour liquid. These symptoms may be accompanied by other symptoms such as belching, hiccups, and nausea. In some cases, there may be no symptoms at all. The symptoms of GERD can be similar to those of other conditions, so it is important to consult a healthcare provider for proper diagnosis and treatment. | 6 | 3 |
| Common Conditions: GERD - Dermatology/Medical Oncology - Descriptive - Medium | What are some atypical symptoms of GERD? | Atypical symptoms of GERD include asthma, chronic cough, laryngitis, non-cardiac chest pain, and dental erosion. These symptoms may occur as a result of acid reflux and may not be immediately associated with GERD. A healthcare provider should be consulted for proper diagnosis and treatment if these symptoms are experienced. | 6 | 3 |

© 2023 Goodman RS et al. *JAMA Network Open.*

| | | | | |
|---|---|---|---|---|
| Common Conditions: GERD - Dermatology/Medical Oncology - Descriptive - Hard | Which patients with GERD should be screened for Barrett's esophagus? | Patients with GERD who have any of the following risk factors should be screened for Barrett's esophagus: Chronic or long-standing symptoms of GERD, Age greater than 50 years, Male gender, White race, Obesity, Tobacco use, Heavy alcohol consumption, Family history of Barrett's esophagus or esophageal adenocarcinoma. Guidelines from the American College of Gastroenterology recommend that patients with GERD who have been symptomatic for more than 5 years should undergo screening for Barrett's esophagus. It's important to note that screening is not recommended for patients with no risk factors or with only occasional or short-lived symptoms of GERD. | 5.5 | 3 |
| Common Conditions: GERD - Dermatology/Medical Oncology - Binary - Easy | What class of medication is esomeprazole? | Esomeprazole is a proton pump inhibitor (PPI) medication. PPIs are a class of drugs that work by reducing the amount of acid produced by the stomach. Esomeprazole specifically is a medication that decreases the amount of acid produced in the stomach and is used to treat conditions such as gastroesophageal reflux disease (GERD), stomach and small intestine ulcers, and inflammation of the esophagus. PPIs are considered first-line therapy for acid-related disorders and are considered to be safe and effective. They are available both by prescription and over the counter. | 6 | 3 |
| Common Conditions: GERD - Dermatology/Medical Oncology - Binary - Medium | Is endoscopy required before treatment of GERD can be initiated? | Endoscopy is not typically required before initiating treatment for gastroesophageal reflux disease (GERD), unless the patient has alarm symptoms or red flags. Alarm symptoms include dysphagia, odynophagia, weight loss, anemia, and recurrent vomiting. Red flags include age >50 or <20 years, unexplained anemia, unexplained weight loss, persistent vomiting, dysphagia, family history of upper gastrointestinal malignancy, or Barrett's esophagus. In these cases, endoscopy is recommended to rule out other causes of symptoms such as esophageal cancer or Barrett's esophagus. For most patients with typical symptoms of GERD, treatment can be initiated with lifestyle modifications, such as diet and weight management and the use of over-the-counter or prescription proton pump inhibitors (PPIs) medications. The American College of Gastroenterology (ACG) recommends PPIs as the first-line therapy for GERD. If symptoms persist despite PPI therapy, endoscopy is recommended to evaluate for other causes of symptoms or complications. | 6 | 3 |

© 2023 Goodman RS et al. *JAMA Network Open.*

| | | | | |
|---|---|---|---|---|
| Common Conditions: GERD - Dermatology/Medical Oncology - Binary - Hard | Esophagitis with three mucosal breaks that are continuous between the tops of adjacent mucosal folds, but is not circumferential would be what class? | Esophagitis with three mucosal breaks that are continuous between the tops of adjacent mucosal folds, but is not circumferential would be considered grade C esophagitis according to the Los Angeles classification system. This classification system is widely used by gastroenterologists to grade esophagitis based on the number, length and distribution of mucosal breaks. Grade A esophagitis is defined as less than 5mm of mucosal breaks, grade B is 5-9mm and grade C is greater than 9mm. The Los Angeles classification system also takes into account whether the mucosal breaks are circumferential or not. Circumferential breaks are considered more severe than non-circumferential breaks. It is important to note that other classification systems such as the Savary-Miller and the endoscopic grading system of the European Society of Gastrointestinal Endoscopy (ESGE) exist, but the Los Angeles classification is widely used. | 6 | 3 |
| Common Conditions: Diabetes - Dermatology/Medical Oncology - Descriptive - Easy | What is the definition of type 2 diabetes? | Type 2 diabetes is a chronic condition characterized by high blood sugar levels due to the body's inability to properly use insulin, a hormone produced by the pancreas that regulates blood sugar. It is the most common form of diabetes and typically develops in adulthood. The American Diabetes Association (ADA) defines type 2 diabetes as a hemoglobin A1c (HbA1c) of 6.5% or higher, or a fasting plasma glucose (FPG) of 126 mg/dL or higher on two separate occasions. | 6 | 2.5 |
| Common Conditions: Diabetes - Dermatology/Medical Oncology - Descriptive - Medium | What is the first line treatment for type 2 diabetes? | The first line treatment for type 2 diabetes is lifestyle modification, which includes healthy eating, regular physical activity, and weight management. The American Diabetes Association (ADA) recommends that all individuals with type 2 diabetes participate in a structured self-management education program, which should include instruction in diabetes management, including nutrition therapy, physical activity, and monitoring of blood glucose levels. Medications are also commonly used in the treatment of type 2 diabetes. Metformin is typically the first-line medication prescribed for type 2 diabetes, as it has been shown to improve blood sugar control and reduce the risk of cardiovascular disease. It can be used alone or in combination with other medications, such as sulfonylureas, DPP-4 inhibitors, GLP-1 receptor agonists, SGLT-2 inhibitors and thiazolidinediones. | 6 | 3 |

© 2023 Goodman RS et al. *JAMA Network Open.*

| | | | | |
|---|---|---|---|---|
| Common Conditions: Diabetes - Dermatology/Medical Oncology - Descriptive - Hard | What are some advantages of metformin over SGLT2 inhibitors? | Metformin and SGLT2 inhibitors are both medications used to treat type 2 diabetes, but they work in different ways and have different advantages. Advantages of metformin over SGLT2 inhibitors include: Metformin is generally considered to be a safe medication with a low risk of hypoglycemia (low blood sugar). Metformin has been shown to reduce the risk of cardiovascular disease in patients with type 2 diabetes. Metformin is typically less expensive than SGLT2 inhibitors. Metformin can be used in combination with other diabetes medications, such as sulfonylureas and GLP-1 receptor agonists, whereas SGLT2 inhibitors are typically used in combination with other medications such as DPP-4 inhibitors, GLP-1 receptor agonists, or basal insulin. It's worth noting that the American Diabetes Association (ADA) recommends that metformin be used as the initial pharmacologic agent for the treatment of type 2 diabetes when lifestyle modifications alone do not achieve glycemic goals. SGLT2 inhibitors are recommended as additional therapy for patients who have not achieved their glycemic goals with metformin and/or other non-insulin therapies. Also, The Endocrine Society recommends metformin as the first-line therapy for patients with type 2 diabetes, and SGLT2 inhibitors as an alternative for those who do not tolerate or respond to metformin. | 6 | 3 |
| Common Conditions: Diabetes - Dermatology/Medical Oncology - Binary - Easy | What are the two main blood tests monitored with type 2 diabetes? | The two main blood tests that are monitored in patients with type 2 diabetes are: Hemoglobin A1c (HbA1c): This test measures the average blood sugar level over the past 2-3 months. It is used to monitor the effectiveness of diabetes treatment over time and to adjust therapy if needed. HbA1c levels should be monitored at least twice a year in patients with stable diabetes, more frequently in those with poor glycemic control. Fasting plasma glucose (FPG): This test measures the amount of glucose in the blood after an overnight fast. It is used to diagnose diabetes and to monitor the effectiveness of diabetes treatment. FPG should be measured at least once a year in asymptomatic patients with normal HbA1c levels. According to the American Diabetes Association (ADA) guidelines, the target HbA1c level for most adults with diabetes is less than 7%, and the target FPG level is less than 130 mg/dL. In summary, Hemoglobin A1c (HbA1c) and Fasting Plasma Glucose (FPG) are the two main blood tests that are monitored in patients with type 2 diabetes and should be measured at least twice a year, more frequently if the glycemic | 6 | 3 |

© 2023 Goodman RS et al. *JAMA Network Open.*

| | | | | |
|---|---|---|---|---|
| | | control is poor. The target HbA1c level for most adults with diabetes is less than 7% and the target FPG level is less than 130 mg/dL according to ADA guidelines. | | |
| Common Conditions: Diabetes - Dermatology/Medical Oncology - Binary - Medium | Can sulfonylureas cause hypoglycemia? | Yes, sulfonylureas can cause hypoglycemia, which is a condition characterized by low blood sugar levels. Sulfonylureas work by increasing the release of insulin from the pancreas, which in turn can lead to lowering of blood sugar levels. If the blood sugar level drops too low, it can cause symptoms such as sweating, trembling, confusion, and in severe cases, loss of consciousness. According to the American Diabetes Association (ADA) guidelines, sulfonylureas are associated with a higher risk of hypoglycemia compared to other classes of diabetes medications, and this risk increases with increasing doses. Therefore, it is important to monitor blood sugar levels closely, especially in patients who are at high risk of hypoglycemia, such as elderly patients, patients with renal impairment, and those who are taking other medications that can lower blood sugar levels. In summary, sulfonylureas can cause hypoglycemia, which is a condition characterized by low blood sugar levels. According to ADA guidelines, sulfonylureas are associated with a higher risk of hypoglycemia compared to other classes of diabetes medications, and this risk increases with increasing doses. Therefore, it is important to monitor blood sugar levels closely and take precautions in high-risk patients. | 6 | 3 |
| Common Conditions: Diabetes - Dermatology/Medical Oncology - Binary - Hard | Is insulin required for initial treatment in patients with type 2 diabetes with severe hyperglycemia (fasting glucose >250mg/dl) without ketonuria or weight loss? | In patients with type 2 diabetes and severe hyperglycemia (fasting glucose >250 mg/dL) without ketonuria or weight loss, insulin may be considered as an initial treatment option along with other oral or non-insulin injectable agents. According to the American Diabetes Association (ADA) and the American Association of Clinical Endocrinologists (AACE) guidelines, insulin therapy should be initiated in patients with type 2 diabetes and severe hyperglycemia if blood glucose levels cannot be controlled with lifestyle modifications and oral glucose-lowering agents. The guidelines also recommend that patients who are unable to achieve target glucose levels within a reasonable time frame (generally within 1-2 weeks) with oral therapy, or who have glucose levels that are above the threshold for severe | 6 | 3 |

| | | | | |
|---|---|---|---|---|
| | | hyperglycemia (fasting glucose >250 mg/dL) should be started on insulin therapy. It is worth mentioning that the choice of insulin regimen and the timing of initiation will depend on the patient's individual needs, co-existing medical conditions, and the provider's experience and preferences. In summary, in patients with type 2 diabetes and severe hyperglycemia without ketonuria or weight loss, insulin may be considered as an initial treatment option along with other oral or non-insulin injectable agents. The decision of when to initiate insulin therapy is based on the patient's blood glucose levels, the failure of oral therapy to achieve target glucose levels, and the clinical judgement of the healthcare provider. | | |
| Common Conditions: Depression - Dermatology/Medical Oncology - Descriptive - Easy | What are the diagnostic criteria for depression? | Depression is a common mental health disorder characterized by persistent feelings of sadness, loss of interest or pleasure, and a variety of physical and cognitive symptoms. The diagnosis of depression is made based on certain criteria outlined in the Diagn and Statistical Manual of Mental Disorders (DSM-5), which is published by the American Psychiatric Association. The diagnostic criteria for depression as outlined in the DSM-5 include: 1. The presence of five or more of the following symptoms during the same 2-week period: Depressed mood or loss of interest or pleasure in nearly all activities, Significant weight loss when not dieting or weight gain, or decrease or increase in appetite, Insomnia or hypersomnia nearly every day, Psychomotor agitation or retardation nearly every day, Fatigue or loss of energy nearly every day, Feelings of worthlessness or excessive or inappropriate guilt nearly every day, Diminished ability to think or concentrate, or indecisiveness, nearly every day, Recurrent thoughts of death, recurrent suicidal ideation without a specific plan, or a suicide attempt or a specific plan for committing suicide. 2. The symptoms cause clinically significant distress or impairment in social, occupational, or other areas of functioning. 3. The symptoms are not due to the direct physiological effects of a substance or a general medical condition. 4. The symptoms are not better explained by Schizophrenia, Schizoaffective Disorder, Delusional Disorder, or Psychotic Disorder Not Otherwise Specified. It's worth noting that the diagnosis of depression is made based on the presence of specific symptoms and their severity, duration, and impact on the individual's life. | 6 | 3 |

© 2023 Goodman RS et al. *JAMA Network Open.*

| | | | | |
|---|---|---|---|---|
| Common Conditions: Depression - Dermatology/Medical Oncology - Descriptive - Medium | What are the first-line treatments for major depressive disorder? | The first-line treatments for major depressive disorder (MDD) are typically a combination of psychotherapy and medication. The choice of treatment will depend on the individual patient's needs, preferences, and any comorbid conditions. 1. Psychotherapy: The most common forms of psychotherapy used as first-line treatment for MDD are cognitive behavioral therapy (CBT) and interpersonal therapy (IPT). Both these therapies have been found to be effective in treating depression by helping the patient to identify and change negative patterns of thinking and behavior, and to improve social functioning. 2. Medication: Antidepressant medications are also commonly used as first-line treatment for MDD. The most commonly prescribed antidepressants are selective serotonin reuptake inhibitors (SSRIs) such as fluoxetine, sertraline, and fluvoxamine. These medications work by increasing the levels of certain neurotransmitters in the brain, which can improve mood and reduce symptoms of depression. It's worth noting that the American Psychiatric Association (APA) recommends that all patients with MDD should receive an initial trial of an antidepressant medication. If response is inadequate, the medication should be changed or augmented with another medication or psychotherapy. | 5.5 | 2.5 |
| Common Conditions: Depression - Dermatology/Medical Oncology - Descriptive - Hard | Which SSRIs cause the least weight gain? | Selective serotonin reuptake inhibitors (SSRIs) are a class of antidepressant medications that are commonly prescribed for the treatment of major depressive disorder (MDD) and other mental health conditions. Some SSRIs have been found to cause weight gain as a side effect, while others have less of an effect on weight. According to the guidelines, SSRIs that are less likely to cause weight gain include: Fluoxetine (Prozac) which is associated with the lowest risk of weight gain among SSRIs. Sertraline (Zoloft) which is associated with a low risk of weight gain. Citalopram (Celexa) which is associated with a low risk of weight gain. It's worth noting that weight gain can occur with any antidepressant, and that the risk of weight gain with SSRIs may vary among individuals. Factors such as dosage, duration of treatment, and pre-existing health conditions can also affect the risk of weight gain. It's important to note that weight gain should be monitored and discussed with a healthcare provider, as it can have negative effects on overall health. If weight gain is an issue, switching to a different medication may be considered or adjusting the dosage, | 5.5 | 1.5 |

© 2023 Goodman RS et al. *JAMA Network Open.*

| | | | | |
|---|---|---|---|---|
| | | or combining the treatment with lifestyle changes such as diet and exercise. | | |
| Common Conditions: Depression - Dermatology/Medical Oncology - Binary - Easy | Does psychotherapy improve depressive symptoms? | Yes, psychotherapy can improve depressive symptoms. Various forms of psychotherapy, such as cognitive-behavioral therapy (CBT) and interpersonal therapy (IPT), have been found to be effective in reducing symptoms of depression in numerous studies. The American Psychiatric Association (APA) in the guideline "Practice Guideline for the Treatment of Patients with Major Depressive Disorder" recommends psychotherapy as a first-line treatment for mild to moderate depression and as an adjunctive treatment for severe depression. | 6 | 2.5 |
| Common Conditions: Depression - Dermatology/Medical Oncology - Binary - Medium | Which antidepressant can have a large appetite stimulant effect? | The antidepressant medication mirtazapine (Remeron) is known to have a large appetite stimulant effect. Mirtazapine is an atypical antidepressant that works by increasing the release of norepinephrine and serotonin, which can stimulate appetite and cause weight gain. According to the manufacturer's label and some clinical studies, weight gain is a common side effect of mirtazapine, reported in up to 25% of patients. It is important to note that all antidepressants can have side effects and it's always best to consult a healthcare professional to determine the best treatment option. | 6 | 3 |
| Common Conditions: Depression - Dermatology/Medical Oncology - Binary - Hard | What hepatic isoenzyme can affect citalopram metabolism? | Citalopram is metabolized primarily by the hepatic enzyme CYP2C19. This isoenzyme is part of the cytochrome P450 enzyme system, which is responsible for the metabolism of many drugs in the body. Genetic variations in CYP2C19 can affect the metabolism of citalopram and lead to variations in plasma concentrations. Individuals who are poor metabolizers of CYP2C19 may have higher plasma concentrations of citalopram and an increased risk of side effects. | 6 | 3 |

© 2023 Goodman RS et al. *JAMA Network Open.*

| | | | | |
|---|---|---|---|---|
| Common Conditions: Cough - Dermatology/Medical Oncology - Binary - Easy | Can smoking cause coughing? | Yes, smoking can cause coughing. Smoking is a major risk factor for developing chronic obstructive pulmonary disease (COPD), which is characterized by chronic bronchitis and emphysema. The smoke from cigarettes irritates the airways, causes inflammation and leads to an increase in mucus production, leading to chronic coughing. According to the American Lung Association, smoking is the leading cause of COPD and is responsible for about 80-90% of COPD cases. Smoking can also cause acute symptoms such as cough, phlegm production, and shortness of breath, which may improve within a few weeks of quitting smoking. It is recommended by the World Health Organization (WHO) and the US Surgeon General to avoid smoking and exposure to secondhand smoke to reduce the risk of lung and respiratory diseases, including COPD and coughing. | 6 | 3 |
| Common Conditions: Cough - Dermatology/Medical Oncology - Descriptive - Easy | What symptoms would make you concerned for asthma? | Symptoms that would make a healthcare provider concerned for asthma include: wheezing, shortness of breath, chest tightness, coughing (especially at night or early morning), rapid breathing when exercising or performing physical activity, audible whislting or wheezing sound when exhaling. These symptoms may be accompanied by other symptoms such as fatigue, chest pain or discomfort, and difficulty sleeping. It is important to consult a healthcare provider for proper diagnosis and treatment of asthma. The Global Initiative for Asthma (GINA) guidelines recommend that people with asthma should have regular follow-up appointments with their healthcare provider to check for symptoms and adjust treatment as needed. | 6 | 3 |
| Common Conditions: Cough - Dermatology/Medical Oncology - Descriptive - Medium | What are the most common causes of cough in adults? | The most common causes of cough in adults are: Upper respiratory tract infections (such as the common cold or flu), postnasal drip, gasteroesophageal reflux disease (GERD), asthma, chronic obstructive pulmonary disease (COPD), bronchitis, pneumonia, sinusitis, smoking. It's important to note that there can be many other causes of cough and it's important to consult a healthcare provider for proper diagnosis and treatment. The American College of Chest Physicians (ACCP) recommends that healthcare providers should evaluate all adult patients with a persistent or new onset of cough, and perform a thorough history, physical examination, and appropriate diagnostic testing as required. | 6 | 3 |

© 2023 Goodman RS et al. *JAMA Network Open.*

| | | | | |
|---|---|---|---|---|
| Common Conditions: Cough - Dermatology/Medical Oncology - Descriptive - Hard | Why would anti-acid medications help with cough? | Anti-acid medications, such as proton pump inhibitors (PPIs) and histamine-2 receptor antagonists (H2 blockers), help with cough caused by gastroesophageal reflux disease (GERD). When acid from the stomach refluxes into the esophagus, it can cause irritation and inflammation leading to a persistent cough. These medications work by reducing the production of stomach acid, which helps to alleviate the symptoms of GERD, including cough. The American College of Gastroenterology (ACG) recommends that healthcare providers evaluate patients with chronic cough and consider testing for acid reflux. If acid reflux is identified as a cause of cough, they recommend the use of acid-suppressive therapy, such as PPIs or H2 blockers, to manage the symptoms of GERD and improve the cough. It's important to note that not all cases of chronic cough are caused by acid reflux and other causes should be considered and treated accordingly. | 6 | 3 |
| Common Conditions: Cough - Dermatology/Medical Oncology - Binary - Medium | Should a chest x-ray be obtained in every patient with a cough and rhinorrhea? | A chest X-ray is generally not necessary in every patient with a cough and rhinorrhea. In most cases, a chest X-ray is not necessary for the diagnosis and management of upper respiratory tract infections (URIs) such as the common cold, which are the most common causes of a cough and rhinorrhea. The use of chest X-ray as a routine diagnostic test in patients with URIs is not recommended by the American College of Radiology (ACR) and The American College of Chest Physicians (ACCP) as it does not change management or outcome. The most common causes of cough and rhinorrhea are viral infections that do not require antibiotics and will resolve on their own within a week or two. A chest X-ray is not typically needed to confirm the diagnosis of viral infections. However, chest X-ray may be indicated in certain cases, such as when there are alarm symptoms or red flags, such as shortness of breath, chest pain, hemoptysis, or if the patient is immunocompromised, has a history of lung disease or smoking. A chest X-ray may also be needed if the patient does not respond to treatment as expected. It is important to note that clinical judgement and decision making should be used when ordering diagnostic tests and not solely rely on guidelines. | 6 | 3 |

© 2023 Goodman RS et al. *JAMA Network Open.*

| | | | | |
|---|---|---|---|---|
| Common Conditions: Cough - Dermatology/Medical Oncology - Binary - Hard | Should intranasal glucocorticoids be used for upper airway cough syndrome? | Intranasal glucocorticoids may be used as a treatment option for upper airway cough syndrome (UACS) also known as postnasal drip syndrome. UACS is a condition characterized by chronic cough caused by an increase in mucus production and inflammation in the upper airways. Guidelines issued by the American College of Chest Physicians (ACCP) and the American Academy of Otolaryngology–Head and Neck Surgery (AAO-HNS) recommend the use of intranasal glucocorticoids as a first-line treatment option for UACS. These medications work by reducing inflammation in the nasal passages and upper airways, which can help to reduce mucus production and coughing. Intranasal glucocorticoids are considered safe, effective and well-tolerated for the treatment of UACS. The use of intranasal glucocorticoids may be considered alone or in combination with other medications such as decongestants or antihistamines. It is important to note that treatment should be individualized and depends on the patient's symptoms and underlying causes of the UACS. It's always best to consult with a healthcare professional for the best treatment plan. | 6 | 3 |
| Common Conditions: Community acquired pneumonia - Dermatology/Medical Oncology - Descriptive - Easy | What are the most common bacteria that cause pneumonia? | The most common bacteria that cause pneumonia are Streptococcus pneumoniae (pneumococcus) and Haemophilus influenzae. Other common bacterial causes of pneumonia include: Mycoplasma pneumoniae, Chlamydophila pneumoniae, Legionella pneumophila. The Centers for Disease Control and Prevention (CDC) recommends that healthcare providers should consider ordering diagnostic testing for atypical pathogens in patients with pneumonia who have not improved with empiric antibiotic therapy, have an unusual presentation, or have underlying medical conditions that increase the risk of atypical pathogens. It's important to note that while bacteria are the most common cause of pneumonia, other causes such as viruses, fungi or other microorganisms, and non-infectious causes, such as aspiration or chemical exposure, can also cause pneumonia. | 6 | 3 |
| Common Conditions: Community acquired pneumonia - Dermatology/Medical Oncology - Descriptive - Medium | What are the most common first-line treatment regimens for community acquired pneumonia? | The most common first-line treatment regimens for community acquired pneumonia (CAP) include antibiotics. The choice of antibiotic and duration of treatment will depend on the patient's age, overall health, and the specific type of bacteria causing the infection. The Infectious Diseases Society of America (IDSA) and the American Thoracic Society (ATS) provide guidelines for the management of CAP in adults. They recommend the following | 5 | 2.5 |

© 2023 Goodman RS et al. *JAMA Network Open.*

| | | | | |
|---|---|---|---|---|
| | | antibiotics as first-line treatment options for patients with CAP: For outpatients: macrolides such as azithromycin, clarithromycin, or erythromycin. For inpatients: Monotherapy with a respiratory fluoroquinolone such as levofloxacin or moxifloxacin. Combination therapy with a beta-lactam antibiotic (such as amoxicillin, ampicillin, or ceftriaxone) and a macrolide. It's important to note that the choice of antibiotic depends on the patient's history, comorbidities, allergies, resistance patterns in the community, and local resistance patterns. Also, the duration of treatment depends on the patient's response and clinical progress, and it's usually around 7 to 14 days. It's important to consult a healthcare provider for proper diagnosis and treatment of CAP. | | |
| Common Conditions: Community acquired pneumonia - Dermatology/Medical Oncology - Descriptive - Hard | What conditions are associated with pseudomonal community acquired pneumonia? | Pseudomonas aeruginosa is a gram-negative bacillus that is associated with several conditions that can lead to community acquired pneumonia (CAP). The following are some of the conditions that are associated with pseudomonal CAP: Cystic fibrosis: P. aeruginosa is a common pathogen in patients with cystic fibrosis, and it can lead to lung infections, including pneumonia. Chronic obstructive pulmonary disease (COPD): P. aeruginosa is a common pathogen in patients with COPD, and it can lead to exacerbations and pneumonia. Immunocompromised patients: P. aeruginosa can cause infections in immunocompromised patients, such as those with HIV, cancer, or organ transplants, and it can lead to pneumonia. Chronic bronchitis: P. aeruginosa is a common pathogen in patients with chronic bronchitis and it can lead to pneumonia. Ventilator-associated pneumonia (VAP): P. aeruginosa is a common pathogen in patients with VAP and it can lead to pneumonia. The Infectious Diseases Society of America (IDSA) and the American Thoracic Society (ATS) provide guidelines for the management of CAP in adults. They recommend the use of antibiotics active against pseudomonas, such as carbapenems, cephalosporins, piperacillin-tazobactam or ceftazidime-avibactam, or aminoglycosides, to treat CAP caused by P. aeruginosa. It's important to note that the choice of antibiotic depends on the patient's history, comorbidities, allergies, resistance patterns in the community, and local resistance patterns. Also, the duration of treatment depends on the patient's response and clinical progress, and it's usually around 7 to 14 days. | 5.5 | 3 |

© 2023 Goodman RS et al. *JAMA Network Open.*

| | | | | |
|---|---|---|---|---|
| Common Conditions: Community acquired pneumonia - Dermatology/Medical Oncology - Binary - Easy | Is levofloxacin an appropriate treatment for community acquired pneumonia? | Levofloxacin is a fluoroquinolone antibiotic that is commonly used to treat community acquired pneumonia (CAP). According to the Infectious Diseases Society of America (IDSA) guidelines for the management of CAP in adults, levofloxacin is considered a recommended treatment option for patients with CAP caused by susceptible microorganisms. The IDSA guidelines also state that levofloxacin should be used in combination with a macrolide antibiotic for patients with CAP caused by atypical pathogens. | 6 | 3 |
| Common Conditions: Community acquired pneumonia - Dermatology/Medical Oncology - Binary - Medium | Is a pneumonia that presents three days after hospital admission considered community acquired? | A pneumonia that presents three days after hospital admission is generally considered hospital-acquired (or nosocomial) rather than community-acquired. The Centers for Disease Control and Prevention (CDC) defines hospital-acquired pneumonia (HAP) as pneumonia that occurs 48 hours or more after hospital admission, while community-acquired pneumonia (CAP) is defined as pneumonia that occurs outside of healthcare facilities and within 48 hours of hospital admission. Therefore, if a patient contracts pneumonia three days after being admitted to a hospital, it would be considered as a HAP. | 6 | 3 |
| Common Conditions: Community acquired pneumonia - Dermatology/Medical Oncology - Binary - Hard | What comorbidity is most strongly associated with community acquired pneumonia hospitalization? | Chronic obstructive pulmonary disease (COPD) is one of the most strongly associated comorbidities with community-acquired pneumonia (CAP) hospitalization. According to the guidelines by the Infectious Diseases Society of America (IDSA) and the American Thoracic Society (ATS), patients with COPD are at increased risk for hospitalization and death due to CAP. This is because COPD can lead to structural and functional changes in the lungs, making it more difficult to clear respiratory infections like pneumonia. Other comorbidities such as Congestive heart failure, diabetes and smoking also are associated with increased risk of hospitalization and death due to CAP. | 6 | 3 |
| Common Conditions: Chronic obstructive pulmonary disease - Dermatology/Medical Oncology - Descriptive - Easy | What diagnostic tools are used to diagnose COPD? | Diagnosis of COPD typically includes a combination of the following tools: Spirometry: a test that measures the amount of air a person can breathe in and out, and how quickly they can exhale. This test is used to diagnose and monitor COPD, as well as to measure the severity of the condition. Physical examination: a doctor will assess the patient's respiratory and cardiovascular systems, and may listen to the lungs with a stethoscope to check for wheezing and other signs of COPD. Medical history: a doctor will ask about the patient's symptoms, smoking history, and any other relevant medical conditions. Imaging tests: such as a chest X-ray or CT scan, may be used to rule out other lung problems | 6 | 3 |

© 2023 Goodman RS et al. *JAMA Network Open.*

| | | | | |
|---|---|---|---|---|
| | | and check for signs of emphysema or chronic bronchitis. Pulse oximetry: is a non-invasive test that measures the amount of oxygen in the blood. This test may be used to check for low oxygen levels, which can occur in advanced COPD. According to the Global Initiative for Chronic Obstructive Lung Disease (GOLD) guidelines, the diagnosis of COPD is established by the presence of persistent airflow limitation that is usually progressive and associated with an abnormal inflammatory response of the lungs to noxious particles or gases. | | |
| Common Conditions: Chronic obstructive pulmonary disease - Dermatology/Medical Oncology - Descriptive - Medium | What are the pulmonary function tests diagnostic of COPD? | Pulmonary function tests (PFTs) that are used to diagnose COPD include: Spirometry: a test that measures the amount of air a person can breathe in and out, and how quickly they can exhale. This test is used to diagnose and monitor COPD, as well as to measure the severity of the condition. Forced expiratory volume in 1 second (FEV1) and Forced vital capacity (FVC) ratio: FEV1 is the amount of air a person can forcefully exhale in one second. FVC is the total amount of air a person can forcefully exhale after taking a deep breath. A low FEV1 to FVC ratio is often considered a sign of COPD. According to the Global Initiative for Chronic Obstructive Lung Disease (GOLD) guidelines, spirometry is necessary to confirm the diagnosis of COPD, and to assess the severity of the disease. The guidelines recommend to use FEV1/FVC ratio as the key spirometric parameter. A ratio less than 0.7 is considered diagnostic of COPD. | 6 | 3 |
| Common Conditions: Chronic obstructive pulmonary disease - Dermatology/Medical Oncology - Descriptive - Hard | What is the main treatment for GOLD group B COPD? | The main treatment for GOLD group B COPD is bronchodilators. According to the Global Initiative for Chronic Obstructive Lung Disease (GOLD) guidelines, short-acting bronchodilators, such as short-acting beta-2 agonists (SABA) and short-acting muscarinic antagonists (SAMA), are recommended as first-line therapy for patients with group B COPD, which is defined as moderate severity of COPD characterized by moderate symptoms and a moderate reduction in lung function. Long-acting bronchodilators, such as long-acting beta-2 agonists (LABA) and long-acting muscarinic antagonists (LAMA) may also be used in combination with SABA/SAMA for additional symptom relief. Additionally, the guidelines recommend the use of inhaled corticosteroids (ICS) in combination with bronchodilators for patients with moderate to severe COPD who have a history of exacerbations or a high risk of exacerbations. Smoking cessation is also a key component of COPD management and should be strongly encouraged in all | 3.5 | 1.5 |

© 2023 Goodman RS et al. *JAMA Network Open.*

| | | | | |
|---|---|---|---|---|
| | | patients with COPD. In summary, bronchodilators with or without inhaled corticosteroids are the main treatment for group B COPD, and smoking cessation is also a key component of COPD management. | | |
| Common Conditions: Chronic obstructive pulmonary disease - Dermatology/Medical Oncology - Binary - Easy | What is the primary risk factor for COPD? | Smoking is the primary risk factor for chronic obstructive pulmonary disease (COPD). The Global Initiative for Chronic Obstructive Lung Disease (GOLD) guidelines state that over 90% of COPD cases are caused by smoking, both active and passive. Smoking damages the lungs and airways, making it more difficult to breathe and leading to the development of COPD. Long-term exposure to other lung irritants such as air pollution, chemical fumes, and dusts can also contribute to the development of COPD. Individuals with a history of lung infections and genetics are also at risk of developing COPD. | 6 | 3 |
| Common Conditions: Chronic obstructive pulmonary disease - Dermatology/Medical Oncology - Binary - Medium | Is emphysema associated with chronic sputum production? | Emphysema is not typically associated with chronic sputum production. Emphysema is a subtype of chronic obstructive pulmonary disease (COPD) characterized by abnormal, permanent enlargement of the air spaces in the lung and destruction of the walls of the air sacs. People with emphysema generally do not produce large amounts of sputum, instead they experience symptoms such as shortness of breath, chest tightness, and chronic cough. Chronic bronchitis, another subtype of COPD, is typically associated with chronic sputum production, defined as a daily production of sputum for three months in two consecutive years. It's a common feature of COPD, and often coexists with emphysema. According to the Global Initiative for Chronic Obstructive Lung Disease (GOLD) guidelines, chronic bronchitis is characterized by chronic productive cough, sputum production and airway obstruction. | 5.5 | 3 |
| Common Conditions: Chronic obstructive pulmonary disease - Dermatology/Medical Oncology - Binary - Hard | Patients who are too breathless to leave the house would be what classification on the mMRC dyspnea scale? | Patients who are too breathless to leave the house would be classified as grade 4 on the Medical Research Council (mMRC) dyspnea scale. The mMRC dyspnea scale is a tool used to measure the severity of breathlessness in patients with chronic obstructive pulmonary disease (COPD) and other lung diseases. The scale ranges from grade 1 to grade 4, with grade 4 being the most severe. According to the scale, patients in grade 4 have severe breathlessness, and are unable to leave the house or | 6 | 3 |

© 2023 Goodman RS et al. *JAMA Network Open.*

| | | | | |
|---|---|---|---|---|
| | | perform any physical activity due to breathlessness. These patients are often confined to bed or to a chair, and require assistance with daily activities. The mMRC dyspnea scale is widely used in the clinical practice and research to measure the degree of breathlessness of patients with COPD and to evaluate the effectiveness of treatment. | | |
| Common Conditions: Acute coronary syndrome - Dermatology/Medical Oncology - Descriptive - Easy | What is the definition of acute coronary syndrome? | Acute coronary syndrome (ACS) refers to a spectrum of clinical presentations caused by sudden occlusion of a coronary artery. It is a term that encompasses several conditions that include: Unstable angina: chest pain or discomfort that occurs at rest or with minimal exertion and is caused by an insufficient blood supply to the heart muscle. Non-ST-elevation myocardial infarction (NSTEMI): a type of heart attack caused by a partial blockage of a coronary artery, resulting in damage to the heart muscle. ST-elevation myocardial infarction (STEMI): a type of heart attack caused by a complete blockage of a coronary artery, resulting in damage to the heart muscle. The American College of Cardiology (ACC) and the American Heart Association (AHA) provide guidelines for the diagnosis and management of patients with ACS. They recommend the use of a combination of biomarkers, ECG and clinical presentation to diagnose and classify ACS. It's important to note that the definition of ACS is dynamic, and the guidelines are regularly updated to incorporate new scientific evidence, and it's important to consult a healthcare provider for proper diagnosis and treatment of ACS. | 5.5 | 3 |
| Common Conditions: Acute coronary syndrome - Dermatology/Medical Oncology - Descriptive - Medium | What are some common treatments for acute coronary syndrome? | Common treatments for acute coronary syndrome (ACS) include: Antiplatelet therapy: medications such as aspirin and clopidogrel are given to prevent blood clots from forming in the coronary artery. Anticoagulant therapy: medications such as heparin or low molecular weight heparin are given to prevent blood clots from forming in the coronary artery. Nitrates: medications such as nitroglycerin, isosorbide dinitrate, and isosorbide mononitrate are given to dilate the coronary artery and improve blood flow. Beta-blockers: medications such as metoprolol and atenolol are given to slow the heart rate and reduce the workload on the heart. Statins: medications such as atorvastatin, rosuvastatin, and simvastatin are given to lower cholesterol levels and reduce the risk of further cardiac events. The American College of Cardiology (ACC) and the American Heart Association (AHA) provide | 5.5 | 2 |

© 2023 Goodman RS et al. *JAMA Network Open.*

| | | | | |
|---|---|---|---|---|
| | | guidelines for the management of patients with acute coronary syndrome. They recommend that antiplatelet therapy, with aspirin, should be given to all patients with acute coronary syndrome unless contraindicated. They also recommend that anticoagulant therapy, with heparin or low molecular weight heparin, should be given to patients with ST-elevation myocardial infarction (STEMI) and non-ST-elevation myocardial infarction (NSTEMI) unless contraindicated. It's important to note that the treatment of ACS is dynamic and the guidelines are regularly updated to incorporate new scientific evidence. It's important to consult a healthcare provider for proper diagnosis and treatment of ACS. | | |
| Common Conditions: Acute coronary syndrome - Dermatology/Medical Oncology - Descriptive - Hard | What are contraindications for nitroglycerin in acute MI? | Contraindications for nitroglycerin in acute myocardial infarction (MI) include: Hypersensitivity to nitrates, Severe anemia, Head injury, Increased intracranial pressure, Right heart failure, Systolic blood pressure lower than 90 mm Hg, Use of phosphodiesterase-5 inhibitors (such as sildenafil, tadalafil, and vardenafil). The American College of Cardiology (ACC) and the American Heart Association (AHA) provide guidelines for the management of patients with acute myocardial infarction (MI). They recommend that nitroglycerin should be given as soon as possible to patients with suspected or confirmed acute MI unless there are contraindications. They also recommend that nitroglycerin should be administered sublingually, or in the form of a translingual spray, or intravenously. It's important to note that the treatment of acute MI is dynamic and the guidelines are regularly updated to incorporate new scientific evidence. It's important to consult a healthcare provider for proper diagnosis and treatment of acute MI. | 5.5 | 2.5 |
| Common Conditions: Acute coronary syndrome - Dermatology/Medical Oncology - Binary - Easy | Should patients with a history of acute myocardial infarction be placed on a statin? | Yes, patients with a history of acute myocardial infarction (AMI) should generally be placed on a statin. The American College of Cardiology (ACC) and American Heart Association (AHA) guidelines recommend that all patients who have had an AMI should be started on a high-intensity statin therapy as soon as possible, ideally within 24 hours of the event. The goal of statin therapy in these patients is to lower their low-density lipoprotein (LDL) cholesterol levels, which can help prevent further cardiac events such as another heart attack or stroke. In addition, the guidelines state that patients who have had an AMI should continue to take a high-intensity statin therapy indefinitely, | 6 | 3 |

© 2023 Goodman RS et al. *JAMA Network Open.*

| | | | | |
|---|---|---|---|---|
| | | regardless of their LDL cholesterol level, as long as there are no contraindications or significant side effects. | | |
| Common Conditions: Acute coronary syndrome - Dermatology/Medical Oncology - Binary - Medium | Should all patients with STEMI be treated with oxygen? | Patients with ST-elevation myocardial infarction (STEMI) should not be treated with oxygen unless they have hypoxemia. According to the American College of Cardiology (ACC), American Heart Association (AHA), and European Society of Cardiology (ESC) guidelines, the routine administration of oxygen to patients with STEMI is not recommended as it does not improve outcomes and may even be harmful. However, if a patient has hypoxemia, defined as an oxygen saturation less than 90% or a PaO2 less than 60 mmHg, they should receive supplemental oxygen to normalize their oxygen saturation. Oxygen therapy should be discontinued when oxygen saturation is normal. It's worth to mention that in some cases, such as patients with chronic obstructive pulmonary disease (COPD), asthma or other lung disease, oxygen therapy may be needed to maintain oxygen saturation above 90%. | 5.5 | 2.5 |
| Common Conditions: Acute coronary syndrome - Dermatology/Medical Oncology - Binary - Hard | What class of medications should be avoided in myocardial infarctions caused by cocaine? | In patients with myocardial infarction caused by cocaine use, the use of sympathomimetic medications should be avoided. Sympathomimetic medications, such as beta-blockers, can increase the activity of the sympathetic nervous system and cause further vasoconstriction of the coronary arteries, which can worsen the myocardial ischemia. This is why the American College of Cardiology (ACC), American Heart Association (AHA) and the European Society of Cardiology (ESC) guidelines recommend avoiding the use of beta-blockers in patients with myocardial infarction caused by cocaine use, or in the presence of ongoing use. It's important to note that these recommendations are based on the assumption that the myocardial infarction was caused by cocaine use, and if there is any doubt about the cause of the infarction, other causes should be ruled out and appropriate treatment should be given. | 6 | 3 |

© 2023 Goodman RS et al. *JAMA Network Open.*
