# Supplementary material for: Accuracy and Reliability of Chatbot Responses to Physician Questions
Source: JAMA Netw Open. 2023 Oct 2;6(10):e2336483. doi: 10.1001/jamanetworkopen.2023.36483 (PMC10546234; doi:10.1001/jamanetworkopen.2023.36483)
Supplement: Supplement 2. — Data Sharing Statement [file jamanetwopen-e2336483-s002.pdf]

Data Sharing Statement

Goodman. Accuracy and Reliability of Chatbot Responses to Physician Questions. *JAMA Netw Open*. Published October 02, 2023. doi:10.1001/jamanetworkopen.2023.36483

Data
Data available: No
